# Combination of arsenic trioxide and apatinib synergistically inhibits small cell lung cancer by down-regulating VEGFR2/mTOR and Akt/c-Myc signaling pathway via GRB10

**DOI:** 10.1186/s41065-024-00330-2

**Published:** 2024-09-02

**Authors:** Yao Yu, Yu Shang, Si Shi, Yaowu He, Wenchao Shi, Menghan Wang, Qi Wang, Dandan Xu, Ce Shi, Hong Chen

**Affiliations:** 1 Harbin Medical University, Harbin, Heilongjiang Province China; 2 Department of Pulmonary and Critical Care Medicine, The Second Affiliated Hospital of Harbin Medical University, Harbin, Heilongjiang Province China; 3 Department of Respiration, The First Hospital of Harbin, Harbin, Heilongjiang Province China; 4 Department of Pulmonary and Critical Care Medicine, The Fourth Affiliated Hospital of Harbin Medical University, Harbin, Heilongjiang Province China; 5 Department of Geriatric Respiratory Medicine, Heilongjiang Provincial Hospital, Harbin, China; 6 NHC Key Laboratory of Cell Transplantation, The First Affiliated Hospital of Harbin Medical University (HMU), Harbin, Heilongjiang Province China

**Keywords:** Small cell lung cancer, Apatinib, Arsenic trioxide, GRB10, Synergistic effect

## Abstract

**Background:**

Small cell lung carcinoma (SCLC) is characterized by -poor prognosis, -high predilection for -metastasis, -proliferation, and -absence of newer therapeutic options. Elucidation of newer pathways characterizing the disease may allow for development of targeted therapies and consequently favorable outcomes.

**Methods:**

The current study explored the combinatorial action of arsenic trioxide (ATO) and apatinib (APA) in vitro and in vivo. In vitro models were tested using -H446 and -H196 SCLC cell lines. The ability of drugs to reduce -metastasis, -cell proliferation, and -migration were assessed. Using bioinformatic analysis, differentially expressed genes were determined. Gene regulation was assessed using gene knock down models and confirmed using Western blots. The in vivo models were used to confirm the resolution of pathognomic features in the presence of the drugs. Growth factor receptor bound protein (GRB) 10 expression levels of human small cell lung cancer tissues and adjacent tissues were detected by IHC.

**Results:**

In combination, ATO and APA were found to significantly reduce -cell proliferation, -migration, and -metastasis in both the cell lines. Cell proliferation was found to be inhibited by activation of Caspase-3, -7 pathway. In the presence of drugs, it was found that expression of GRB10 was stabilized. The silencing of GRB10 was found to negatively regulate the *VEGFR2/Akt/mTOR and Akt/GSK-3β/c-Myc* signaling pathway. Concurrently, absence of metastasis and reduction of tumor volume were confirmed in vivo. The immunohistochemical results confirmed that the expression level of GRB10 in adjacent tissues was significantly higher than that in human small cell lung cancer tissues.

**Conclusions:**

Synergistically, ATO and APA have a more significant impact on inhibiting cell proliferation than each drug independently. ATO and APA may be mediating its action through the stabilization of GRB10 thus acting as a tumor suppressor. We thus, preliminarily report the impact of GRB10 stability as a target for SCLC treatment.

**Supplementary Information:**

The online version contains supplementary material available at 10.1186/s41065-024-00330-2.

## Background

Lung cancers account for nearly 12.2% of all cancers in the World. Histologically, there are two known sub types of lung cancer, namely small cell lung carcinoma (SCLC) and non-small cell lung carcinoma (NSCLC). SCLC contributes to about 15% of all lung cancers globally with data being unavailable on country specific incidence [1]. SCLC has a strong epidemiological link to smoking. This would also explain the decreasing trends in the incidence of SCLC, worldwide with associated lifestyle changes [2]. Nevertheless, SCLCs are characterized by poor prognosis arising due to a strong predilection for metastasis, high rate of proliferation and consequently an increased rate of mortality. It is worth noting that the number of microvessel count (MVC) of SCLC is higher than that of NSCLC, and the protein expression levels of MVC and vascular endothelial growth factor (VEGF) are also correlated with the prognosis of SCLC [3].

Whilst it is known that recurrent mutations may be associated with tumor development, a comprehensive characterization of these genome wide alterations is lacking for SCLC [4]. This lack of information is often attributed to the absence of relevant patient material for in-depth assessment and analyses. While cell lines derived from primary tumors do not adequately represent the tumor microenvironment, mouse models of SCLC are poorly reflective of human biology as the condition is most often associated with smoking [5].

Despite experimental limitations, both genetic and epigenetic modifications have been associated with the development and progression of SCLC. Significant associations have been found between genetic alterations in *TP53, RB1, PI3K* pathway along with members of *MYC* and *NOTCH* family as drivers of SCLC. Despite this elucidation, advances in SCLC treatment have been limited [4, 6]. In the past three decades, chemotherapy has been empowered with immune checkpoint inhibitors (ICIs) for higher efficacy. It has also been shown that patient response to ICIs in SCLC is relatively low [7, 8]. Further, it was found to be driven by low expression of PD-L1, major histocompatibility complex (MHC)-1 and over expression of Tregulatory (Treg) cells [7]. Treatment options in second-line extensive stage SCLC (ED-SCLC) are further limited. Thus, to improve the prognosis of SCLC, the combination therapies may be a promising strategy.

Drugs targeting angiogenesis also seem to be ideal for SCLC therapy. VEGF-A is one of the targets for inhibiting angiogenesis. VEGF-A binds to VEGFR2 (a transmembrane tyrosine kinase receptor, TKI), subsequently inducing endothelial cell proliferation and survival. Other commonly targeted pathways include DNA damage repair (PARP), cell cycle checkpoints (AURKA and CDK4/6), apoptosis (Bcl-2), neuroendocrine differentiation (Dll3/CD3), epigenetics (EZH2 and BET), DNA transcriptional inhibition, and anti-TROP-2 [9]. Nevertheless, antiangiogenic agents were also found to enhance PD-L1/ PD-1 expression along with infiltration of Tregs and myeloid derived suppressor cells (MDSCs) in tumor microenvironment [10]. Although various studies have made some progress, patients with the same tumor stage still have different treatment responses [11]. Tumor escape from immune surveillance in the presence of single agents has also been observed [10]. This prompted us to find more comprehensive treatment options, such as combination therapy with nonspecific targeted agents.

We investigated the role of arsenic trioxide (ATO) and apatinib (APA) as a safe treatment approach in SCLC. As a traditional Chinese medicine, ATO has been a commonly used drug for the treatment of many diseases such as syphilis, psoriasis, and rheumatism for thousands of years. Currently, the FDA has approved its use for the treatment of acute promyelocytic leukemia [12]. While the role ATO in SCLC is less reported, APA (TKI) was found to retard the growth of SCLC cells by downregulating the expression of VEGF, P-VEGFR2, P-PI3K, P‐Akt, P‐ERK1/2, Ki‐67 and CD31 [13]. It was also found that the drugs were effective even in patients with extensive stage of the disease. Our current study aims to explore the combinatorial effects of these two agents, viz., ATO and APA and determine the molecular mechanisms of their mode of action in SCLC through in vitro and in vivo assay.

## Methods

### Cell lines and culture conditions

The human SCLC cell lines NCI-H446 and NCI-H196 were obtained from the Cell Bank of Chinese Academy of Sciences (Shanghai, China). The H446 and H196 cells belonged to the adherent cell of SCLC, which were characterized by high expression of c-Myc and high malignant degree. The human bronchial epithelial (HBE) cell line BEAS-2B were from Blood Cancer Center Laboratory of the First Affiliated Hospital of Harbin Medical University. All cell lines were cultured in complete medium (CM) consisting of RPMI-1640 (Gibco, Beijing, China) supplemented with 10% fetal bovine serum (FBS) (Sigma, Carlsbad, USA) at 37 °C with 5% CO_2_ in a humidified incubator.

### Reagents and chemicals

Arsenic trioxide (ATO) solution was obtained from YiDa Pharmacy (Harbin, China). The stock solution of ATO was 5 mM and stored at 4 ^ο^C away from light. Apatinib (APA) was obtained from Hengrui Medicine Co. Ltd (Jiangsu, China). A 20 mM stock was prepared by solubilizing APA in di methyl sulfoxide (DMSO). The stock was preserved at -20 °C and diluted with RPMI-1640 to the final desired concentration. For in vitro assays care was taken to not exceed DMSO concentration over 0.16%.

### Clinical samples

The studies involving human participants were reviewed and approved by the Medical Ethics Committee of The Second Affiliated Hospital of Harbin Medical University (YJSKY2023-361). The patients who underwent pulmonary surgery in The Second Affiliated Hospital of Harbin Medical University from March 2018 to March 2023 were included. The histopathology of cancer met the diagnostic criteria of small cell lung cancer, and had no other tumors, and neither received chemoradiotherapy, immunotherapy, and targeted therapy before surgery, with a total of 15 cases. Obtain cancer tissue and corresponding adjacent tissue slices from the Pathology Department of the Second Affiliated Hospital of Harbin Medical Universit. All pathological diagnoses were confirmed by two experienced pathologists. The patients provided their written informed consent to participate in this study.

### Short hairpin RNA transfection

To generate a stable GRB10 knockdown cell line, the lentiviral vector VP013-U6-MCS-PGK-PURO (General Biol, Xuzhou, China) was constructed and used. A short hairpin RNA (shRNA) against GRB10 and the corresponding control were cloned into packaging plasmids (General Biol, Xuzhou, China). Lentiviruses were generated using the packaging cell line HEK293T purchased from the American Type Culture Collection (ATCC). The viral supernatant was harvested at 48 h post-transfection, filtered, and stored at -80 °C. For the construction of sh-GRB10-H446 and sh-GRB10-H196 cells, cells of 40% confluency were incubated in a lentivirus-containing medium. The medium was supplemented with 8 µg/mL polybrene for 20 h. Subsequently the cells were resuspended in CM for 48 h, with 2 µg/ml puromycin added to screen out stably transduced cells. The sequence of sh-GRB10 was 5′-GCAGTCAAATGGCAGTCAAAC-3′. Transfection efficiency and knockdown efficiency were assessed by qPCR and Western blot.

### CCK-8 assay

Commercially available CCK-8 assay kit (Dojindo Laboratories, Kumamoto, Japan) was used to test the proliferative inhibition of ATO, APA, and their combination on SCLC cells. H446 and H196 cells were plated into 96-well plates (5 × 10^3^ cells/100 µL/well) and cultured for 24 h. cells were then treated with different concentrations of ATO, APA alone, and the combination of both for 48 h. After an additional incubation for 90 min with CCK-8 solution (10 µL/well) at 37℃, the optical density (OD) was read at 450 nm using a microplate spectrophotometer (Thermo Fisher Scientific, Waltham, MA, USA). Each experiment was performed at least three times. Finally, the GraphPad Prism Version 8 (Insightful Science, LLC, San Diego, California, USA) was applied to calculate the IC50 (50% inhibitory concentration) value.

### Synergy evaluation

We analyzed the combination index (CI) through the isobologram analysis based on medium-drug effect analysis. The statistics from the CCK-8 assay were showed as percent viability and translated to fraction affected (Fa). Fa ranges from 0 to 1, where Fa = 0 means 100% viability, Fa = 1 means 0% viability. The data was then analyzed by CompuSyn program (Biosoft, Cambridge, United Kingdom). The CI values exhibit the interaction modes between ATO and APA. CI ranges from 0 to +∞, when CI < 1, it indicates a synergistic effect between the two drugs, when CI = 1, it indicates an additive effect, and when CI > 1, it indicates antagonism.

### Colony formation assay

H446 and H196 cells were made into single cell suspension and seeded into 6-well plates (500 cells/2 mL/well), with gentle rotation to evenly distribute cells. After maintaining at 37 ℃ and 5% CO_2_ for 24 h, cells were incubated in CM supplemented with respective drugs for 3 days. Upon treatment medium was replaced with CM 10 days. When clones were visible in the Petri dish with naked eyes, the cells were softly washed with PBS twice, and fixed with 4% paraformaldehyde for 25 min. Finally, we stained the cells with 0.5% crystal violet solution for 25 min at room temperature in dark. Imaging was undertaken using a digital camera. The number of visible cell colonies were recorded by the Image J software. Each experiment was performed in triplicates.

### Wound healing assay

H446 and H196 cells were plated into 6-well plates (1 × 10^6^ cells/2 mL/well). When the cell density was about 90% after 24 h. Confluent monolayer cells were scratched in a straight line using a 200 µL pipette tip. The exfoliated cells were softly washed with PBS twice. Then the serum-free RPMI-1640 containing various drugs was used to culture the cells and the cells were allowed to heal the wounds at 37 ℃ and 5% CO_2_ for 24 h. At the same place where cells were scratched, pictures (magnification, 10×) were taken at 0 and 24 h. Finally, the Image J was used to determine the migration area of cells according to the change of wound size.

### Transwell migration and invasion assay

H446 and H196 cells (2.5 × 10^5^) in 200 µL serum-free medium containing various drugs were plated on the transwell upper chamber, with 600 µL medium containing 10% FBS in the transwell lower chamber. The transwell chambers were cultured 37 ℃ and 5% CO_2_ for 48 h. After that, the cells adhering to the bottom membrane were fixed in 4% paraformaldehyde for 30 min, and subsequently dyed with 0.5% crystal violet solution for 30 min at room temperature in dark. The upper chamber was gently cleaned with a cotton swab. Finally, the transwell chambers were inverted and observed under a microscope for photographic recording and the number of cells on the bottom surface was counted. Five random fields were counted per filter in all groups. The invasion assay was performed in the upper layer of transwell chambers covered with Matrigel (Corning, USA).

### Apoptosis assay

H446 and H196 were incubated with different groups of treatment for 48 h. The cells (5 × 10^5^ /mL) were harvested and washed with PBS twice. As per the manufacturer’s (Beyotime, Shanghai, China) instructions, Annexin V-Fluorescein Isothiocyanate (FITC) and propidium iodide (PI) were applied to stain the cells for 15 min at room temperature in dark. Finally, the apoptosis rate of cells was detected using a BD FACSCalibur flow cytometry system (Becton-Dickinson, NJ, USA) within 1 h after staining. The PI staining levels were analyzed using FlowJo software. Each experiment was performed thrice.

### Cell cycle assay

H446 and H196 were incubated with different groups of treatment for 48 h, the cells (5 × 10^5^ /mL) were then harvested and washed with cold PBS twice. The cells were resuspended in cold 70% ethanol at -4 ℃ and away from light for 16 h. Next, cells were washed with 1 mL cold PBS. As per manufacturer’s (Beyotime, Shanghai, China) instructions, propidium iodide (PI) staining solution were applied to stain the cells at 37 ℃ and away from light for 30 min. Finally, the number of cells in each stage of cell cycle was detected using a BD FACSCalibur flow cytometry system (Becton-Dickinson, NJ, USA) within 1 h after staining. The PI staining levels were analyzed using FlowJo software. Each experiment was performed at least thrice.

### Western blot analysis

H446 and H196 were incubated with different groups of treatment for 48 h, the cells were then harvested and split in RIPA lysis buffer with proteinase inhibitors (Beyotime, Shanghai, China). The protein concentration of extracts was estimated by BCA qualitative method (Beyotime, Shanghai, China). A total of 30 µg protein was loaded on a 7.5% or 10% SDS-gel, resolved using SDS-PAGE (EpiZyme, Shanghai, China) and transferred to polyvinylidene fluoride (PVDF) membranes (Millipore, Bedford, MA, USA). After maintaining in 5% nonfat milk in Tris-buffered saline and 0.1% Tween 20 (TBST) for 2 h, the PVDF membranes were washed for 10 min with TBST thrice, and incubated in TBST containing various primary antibodies (VEGFR2, FN1, Akt, P-Akt, mTOR, P-mTOR, β-catenin, c-Myc, PARP, Cleaved PARP, Caspase-3, Cleaved Caspase-3, Cleaved Caspase-7, Cleaved Caspase-9, Bak, Bid, GRB10, P-GSK-3β, MMP9 1:1000, Bcl-2 1:2000, and ACTIN, GAPDH 1:5000) at 4 ℃ overnight. The following day, the PVDF membranes were washed 10 min with TBST thrice and incubated in HRP-conjugated secondary antibodies for 1 h. The membranes were washed again with TBST and visualized with ECL regents (EpiZyme, Shanghai, China). Image J software was used to measure the densitometry of the protein bands. The primary antibodies of VEGFR2, Akt, P-Akt, mTOR, P-mTOR, β-catenin, c-Myc, PARP, Cleaved PARP, Caspase-3, Cleaved Caspase-3, Cleaved Caspase-7, Cleaved Caspase-9, Bak, Bid, P-GSK-3β, MMP9 were purchased from Cell Signaling Technology (Shanghai, China) and the primary antibodies of Bcl-2 were purchased from Abcam (Shanghai, China). The primary antibodies of FN1, GRB10, ACTIN, GAPDH and HRP-conjugated goat anti-mouse and goat anti-rabbit secondary antibodies were purchased from ProteinTech (Wuhan, China). Each experiment was performed at least thrice.

### Real-time quantitative polymerase chain reaction (qPCR)

H446 and H196 were incubated with different groups of treatment for 48 h, the cells were then harvested. The total RNA was extracted from cells using SevenFast Total RNA Extraction Kit (Seven, Beijing, China) and reverse-transcribed into cDNA using a PerfectStart Uni RT&qPCR Kit (TransGen, Beijing, China). qPCR was performed using specific primers and Illumina HiSeq 6000 platform (Illumina, Inc. San Diego, USA). The following primers were used:

VEGFR-2 forward 5’-AAAAGGACCTCGGACTGCAG-3’ and.

reverse 5’-CAGAAGTGTCAGCTCCCCAG-3’;

AGRN forward 5’-CTCAACTCCAGCCTCATGC-3’ and.

reverse 5’-GAAGCCGCACAGCATTC-3’;

FN1 forward 5’-GTGCAGCTGTTTACCAACCG-3’ and.

reverse 5’-ACTTGCCACCGTAAGTCTGG-3’;

GRB10 forward 5’-ACCACGGGCTCTGCATAAAG-3’ and.

reverse 5’-ACGTCCTGGTTTGCTCGTC-3’;

GAPDH forward 5’-GGAGCGAGATCCCTCCAAAAT-3’ and.

reverse 5’-GGCTGTTGTCATACTTCTCATGG-3’.

The relative expression level of target mRNA was calculated after normalization with the expression of GAPDH based on the ΔCt method.

### RNA sequencing

All the RNA-sequencing procedures were performed by the Novogene (Beijing, China). H446 cells were treated with ATO (1 µM), APA (16 µM) alone, and the combination of both for 48 h. The total RNA was extracted from cells using SevenFast Total RNA Extraction Kit (Seven, Beijing, China). Total RNA was used as input material for the RNA sample preparations. Subsequently, these samples were sequenced on the Illumina NovaSeq 6000 platform (Illumina, Inc. San Diego, USA). Differential expression analysis of two groups (three biological replicates per condition) was performed using the DESeq2 R package (1.20.0). DESeq2 provides statistical routines for determining differential expression in digital gene expression data using a model based on the negative binomial distribution. The resulting *P*-values were adjusted using Benjamini and Hochberg’s approach for controlling the false discovery rate. *P-*adj < = 0.05 and |log2(foldchange)| >= 1 were set as the threshold for significantly differential expression. Differentially expressed genes were visualized using Venn and Volcano plots. Gene Ontology (GO) enrichment analysis of differentially expressed genes was implemented by the clusterProfiler R package (3.8.1), which was used to identify the statistical enrichment of differential expression genes in Kyoto Encyclopedia of Genes and Genomes (KEGG) pathways. Using the local version of the GSEA analysis tool (http://www.broadinstitute.org/gsea/index.jsp), GO, KEGG, Reactome, DO and DisGeNET data sets were used for GSEA independently. PPI analysis of differentially expressed genes was based on the STRING database, which is available to known and predicted Protein-Protein Interactions.

### Xenograft mouse model

Five-week-old male BALB/c nude mice were purchased from and raised in the Experimental Animal Center of the Second Affiliated Hospital of Harbin Medical University (Harbin, China). The animal study was reviewed and approved by the Medical Ethics Committee of the Second Affiliated Hospital of Harbin Medical University (YJSDW2022-089). NCI-H446 cells (2 × 10^7^) were subcutaneously injected with 200 µL PBS into the right flanks of mice. After developing tumors 10 days from cell injection, mice were randomly divided into four groups (4 mice per group) and treated with normal saline containing 2% DMSO (control, intragastric administration), 5.0 mg/kg of ATO (intraperitoneal injection), 100 mg/kg of APA (intragastric administration), and combination of ATO and APA, respectively. All agents were administered once every day for 15 days. The long diameter (a) and short diameter (b) of tumors were measured every 3 days, mice were sacrificed, and tumors were extracted. Tumor volume was calculated as (a × b^2^)/2.

### Immunohistochemistry assay

The collected tissue samples were fixed, transparent, embedded and sectioned in sequence. The sections were then dewaxed and hydrated, endogenous peroxidase inactivated, antigen retrieved, blocked, incubated with primary antibody, incubated with secondary antibody, developed with DAB, stained with hematoxylin, reversed with hematoxylin, dehydrated, and mounted. An inverted microscope was used to record immunohistochemical images. Stained sections were examined by a double blind-based procedure. The reagents and antibodies used were as follows, immunohistochemistry kit (ZSGB-BIO, Beijing, China), Anti-GRB10 antibody (1:100) (ProteinTech, Wuhan, China).

### Statistical analysis

GraphPad Prism Version 8 (Insightful Science, LLC, San Diego, California, USA) was used to analyze the data. Data are presented as the mean ± standard deviation (SD). A one-way ANOVA followed by a Tukey’s post hoc test was used to assess the difference between multiple groups. Differences between the two groups were analyzed using a student’s *t*-test. *P* < 0.05 was considered to indicate a statistically significant difference (*P < 0.05; **P < 0.01; ***P < 0.001; ****P < 0.0001).

## Results

### Combination of arsenic trioxide (ATO) and apatinib (APA) synergistically inhibited proliferation, migration, and invasion of SCLC cells

**Fig. 1:**
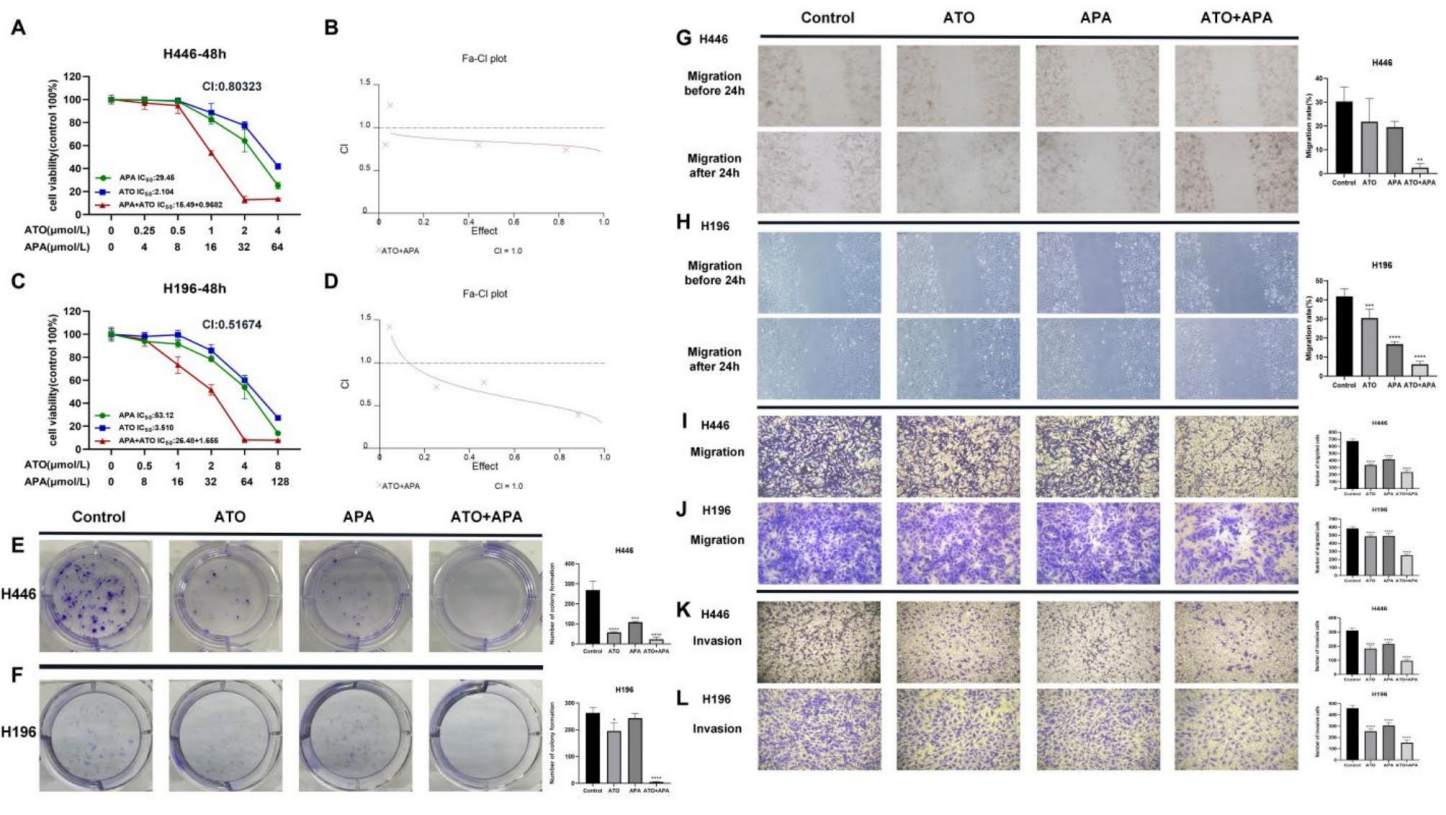
The proliferation inhibitory effect of ATO, apatinib alone, and their combination in the SCLC cells. Drug concentration-cell viability curves were drawn as the percentage of viable cell based on the cell viability assay (**a, c**). The synergistic effects between ATO and apatinib were exhibited as Fa-CI plots (**b, d**). ATO and apatinib suppressed colony formation of H446 (**e**) and H196 (**f**) cells. ATO and apatinib suppressed migration and invasion ability of H446 and H196 cells. Typical images of wound healing showed that, after 24 h treatment with 1 µM of ATO, 16 µM of apatinib or 1 µM of ATO and 16 µM of apatinib in combination of H446 cells (**g**); H196 cells were treated with 2 µM of ATO, 32 µM of apatinib or 2 µM of ATO and 32 µM of apatinib in combination for 24 h (**h**); Transwell assay showed that, after 48 h treatment with 1 µM of ATO, 16 µM of apatinib or 1 µM of ATO and 16 µM of apatinib in combination of H446 cells (**i, k**); H196 cells were treated with 2 µM of ATO, 32 µM of apatinib or 2 µM of ATO and 32 µM of apatinib in combination for 48 h (**j, l**) (magnification, ×100; scale bars, 200 μm); All data are shown as the mean ± SD of three independent experiments. (****, *P* < 0.0001, ***, *P* < 0.001, **, *P* < 0.01, *, *P* < 0.05 versus the control group)

It was found that both ATO and APA inhibited the growth of SCLC cells in a dosage-dependent manner. Compared with the single drug group, the combination group had a stronger inhibitory effect on cell proliferation in SCLC, indicating that the combination of ATO and APA could synergistically inhibit the proliferation of SCLC cells, while reducing the dose of the combination drug (Fig. 1a, c). The synergy evaluation showed the CI values of H446 and H196 cells were less than 1, indicating a synergistic effect between ATO and APA (Fig. 1b, d). Based on the IC50 calculation values of ATO and APA, the H446 cell in the combination group used fixed doses of ATO (1 µmol/L) and APA (16 µmol/L) as the drug dosage, while the H196 cell in the combination group used fixed doses of ATO (2 µmol/L) and APA (32 µmol/L) as the drug dosage. Compared with the single drug group, the number of cell colony formation in the combination group was significantly reduced (Fig. 1e, f). Subsequently, to investigate whether ATO and APA affected the migration and invasion ability of SCLC cells, wound healing (Fig. 1g, h), and transwell assays (Fig. 1i, j, k, l) were performed on H446 and H196 cells. It was showed that, compared with the single drug group, both cells migrated the least areas in the combination group after 24 h, and the combination group had a significant decrease in the number of migrated and invasive cells after 48 h. Collectively, the data demonstrated that the combination of ATO and APA could synergistically inhibit the migration and invasion ability of SCLC cells, and was superior to using ATO or APA alone.

Futher, we found that in BEAS-2B cells, when the concentration of ATO was less than 0.5 µmol/L, it had little effect on cell viability, but when the concentration was more than 2 µmol/L, cell viability significantly decreased. When the concentration of APA exceeded 64 µmol/L, cell viability significantly decreased. However, the effect of the combination of two drugs (1 µmol/L of ATO and 16 µmol/L of APA, 2 µmol/L of ATO and 32 µmol/L of APA) on cell viability was not significantly higher than that of the single drug group of ATO or APA. These results indicated that the drug combinations selected in the experiment did not significantly increase dose-related adverse reactions. Subsequently, there was no significant difference in apoptosis between the combination group and the single drug group, as shown in Supplement 1.

### Combination of ATO and APA synergistically induced apoptosis in SCLC cells by altering cell cycle

**Fig. 2:**
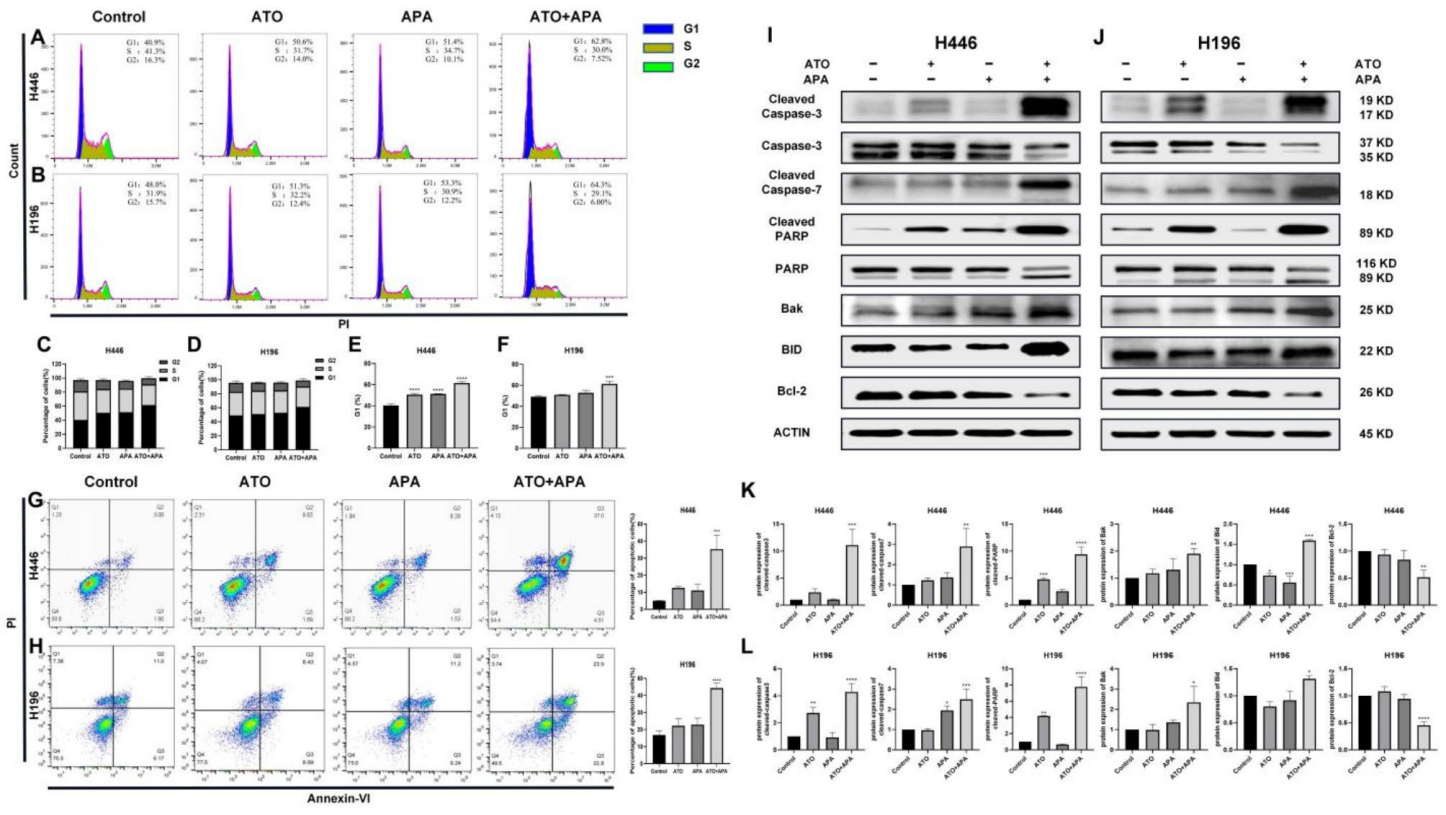
Effect of ATO, apatinib alone and in combination on cell cycle and apoptosis. Representative profiles showing the percentage of H446 cells in G1, S, or G2/M phase after 48 h treatment with 1 µM of ATO, 16 µM of apatinib or 1 µM of ATO and 16 µM of apatinib in combination (**a**); H196 cells were treated with 2 µM of ATO, 32 µM of apatinib or 2 µM of ATO and 32 µM of apatinib in combination (**b**); Histograms represent the cell population in each phase of H446 (**c**) and H196 (**d**); Histograms represent the cell population in G1 phase of H446 (**e**) and H196 (**f**). Representative profiles showing apoptosis in H446 cells after 48 h treatment with 1 µM of ATO, 16 µM of apatinib or 1 µM of ATO and 16 µM of apatinib in combination (**g**); H196 cells were treated with 2 µM of ATO, 32 µM of apatinib or 2 µM of ATO and 32 µM of apatinib in combination (**h**); Then, western blot was used to evaluate the levels of apoptosis-related proteins in H446 (**i**) and H196 (**j**) cells; Relative intensity of protein on apoptosis signaling pathway in H446 (**k**) and H196 (**l**) cells. All data are shown as the mean ± SD of three independent experiments. (****, *P* < 0.0001, ***, *P* < 0.001, **, *P* < 0.01, *, *P* < 0.05 versus the control group)

Due to the increased mortality of SCLC cells caused by the combination of ATO and APA treatment, we examined the change in cell cycle and apoptosis in H446 and H196 cells using Flow cytometry. As shown in Fig. 2a and b, compared with the control group and the single drug ATO or APA group, the combination group of ATO and APA remarkedly enhanced the G1 phase cell population. Subsequently, the combination group of ATO and APA significantly increased the apoptosis of cells (Fig. 2g, h). Meanwhile, Western blot results confirmed that compared with the control group and the single drug ATO or APA group, the combination of the two drugs increased the expression levels of pro-apoptotic proteins Cleaved Caspase-3, Cleaved Caspase-7, Cleaved PARP, Bid, Bak, and decreased the expression levels of anti-apoptotic proteins Bcl-2 (Fig. 2i, j). The above results demonstrated that the combination of ATO and APA could synergistically enhance the cycle arrest, and induce apoptosis of SCLC by activating the Caspase/PARP signaling pathway.

### Combination of ATO and APA synergistically inhibited SCLC growth in vivo

**Fig. 3:**
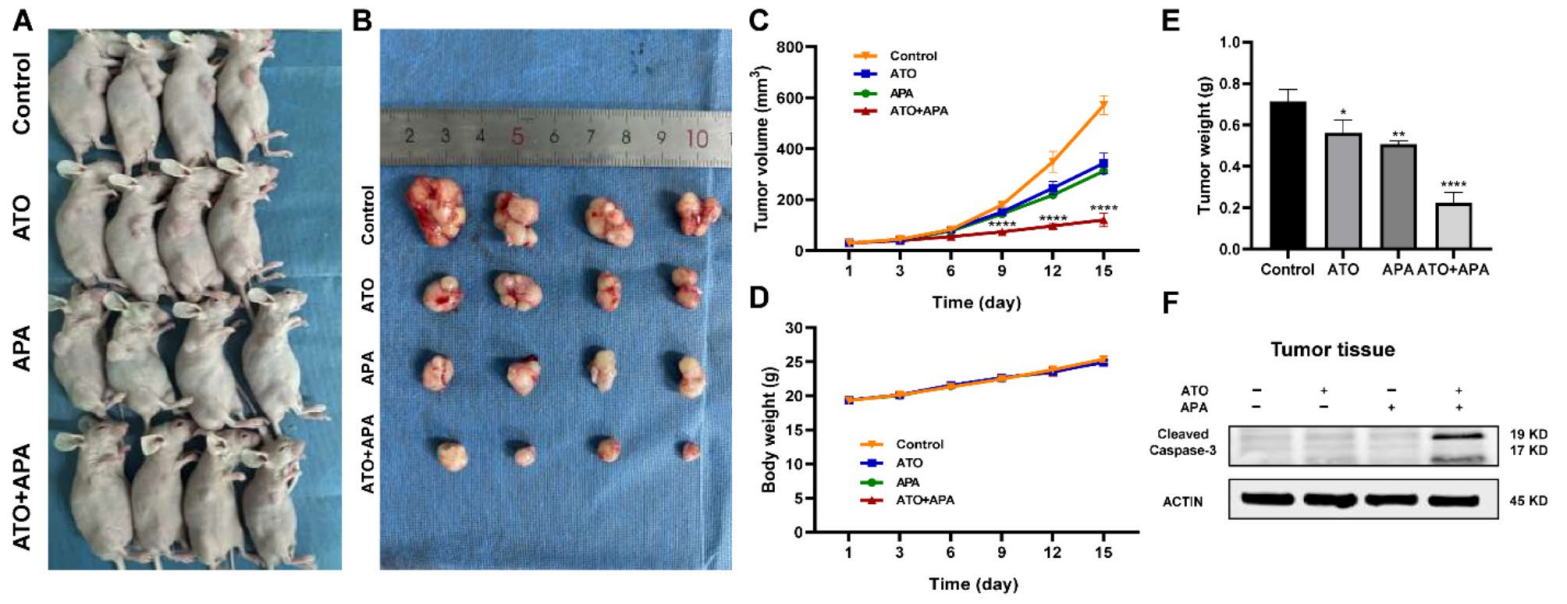
Effect of ATO, apatinib alone and in combination inhibit SCLC growth in vivo. NCI-H446 cells were subcutaneously injected with 200 µL PBS into the right flanks of mice. After 10 days, mice were randomly divided into four groups (4 mice per group) and treated normal saline containing 2% DMSO (control, intragastric administration), 5.0 mg/kg of ATO (intraperitoneal injection), 100 mg/kg of apatinib (intragastric administration), and combination of ATO (5.0 mg/kg)/apatinib (100 mg/kg) once daily, respectively. Images of the mice presented at the end of the treatment (**a**); Images of xenograft tumors dissected from each mouse (**b**); Tumor volumes were indicated by the curves representing the trend in the increase in tumor size (**c**); Body weights of mice were indicated by the curves representing the trend in the increase (**d**); Histograms represent the average weight of tumors (**e**); Then, western blot was used to evaluate the levels of Cleaved Caspase-3 protein in tumor tissue (**f**) (****, *P* < 0.0001, **, *P* < 0.01, *, *P* < 0.05 versus the control group)

A xenograft model experiment was used to further investigate the effects of ATO and APA on SCLC. As shown in Fig. 3c, from day 9, tumor volume in combination group of ATO and APA was significantly lower compared with the control group, furthermore, xenograft tumor volume (Fig. 3c) and weight (Fig. 3e) in the combination group with ATO and APA were lower compared with the single drug ATO or APA group at the end of treatment. Besides, body weights of mice were not significantly different between the groups (Fig. 3d). Further, we detected protein expression levels of Cleaved Caspase-3 of excised tumor tissues. Expression levels of the Cleaved Caspase-3 protein in the combination group were significantly higher than that in the single drug group, as shown in Fig. 3f. HE staining was done on the liver, kidney and heart of the mice after administration, and the results showed that there was no significant difference between the two drug combination group and the single drug group, confirming that the drug dose of the combination group had no significant damage to the liver, kidney and heart of the mice (Supplement 2). Accordingly, these results indicated the combination of ATO and APA synergistically and safely suppressed SCLC growth in vivo.

### Combination of ATO and APA increased expression of GRB10

**Fig. 4:**
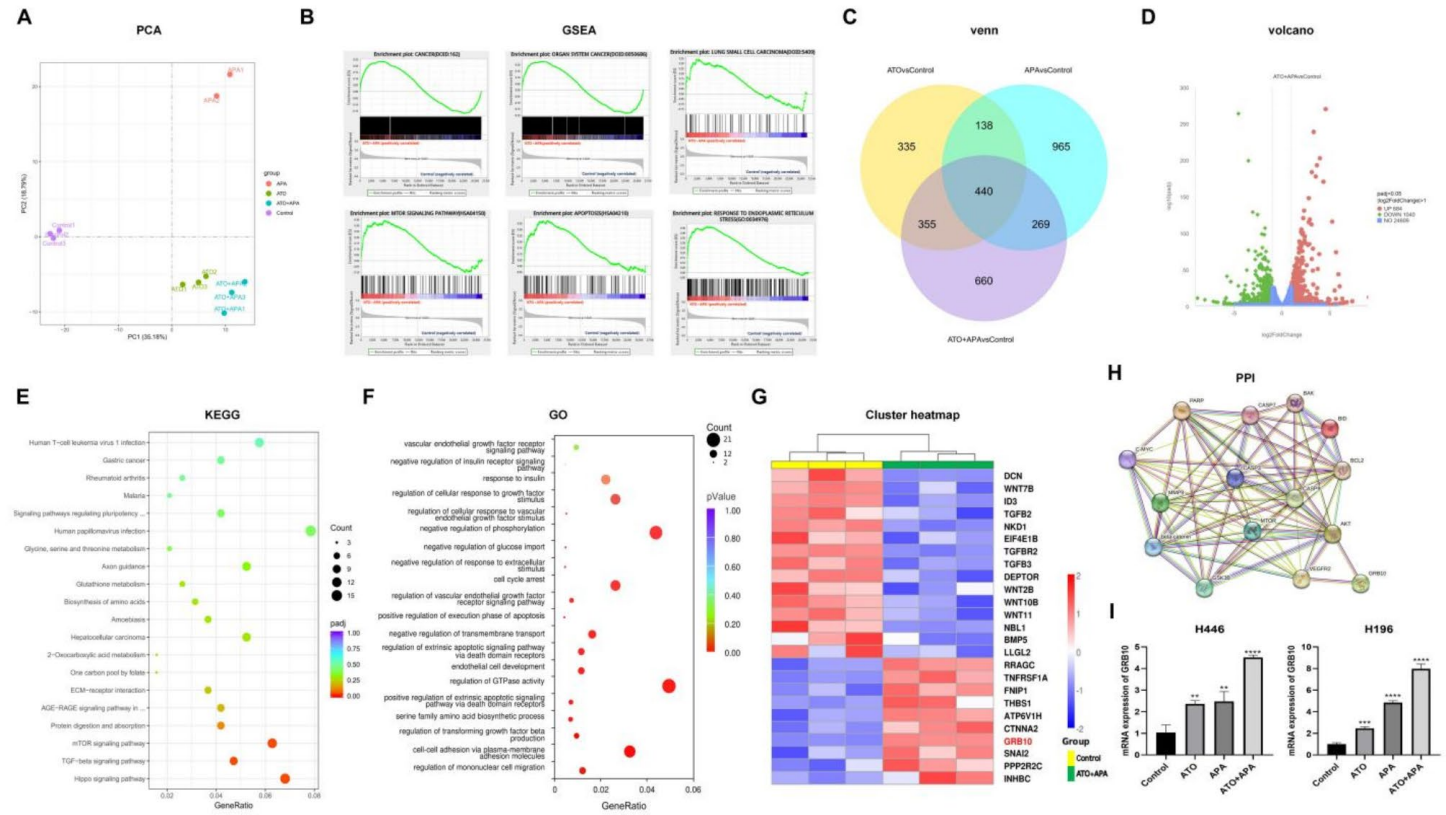
Combination of ATO and apatinib increased expression of GRB10. Principal Component Analysis (PCA) illustrated the distribution of samples that underwent RNA sequencing (**a**). The genes that differed between Control and ATO + APA groups were analysed through GESA (**b**). Differentially expressed genes (|log2FC|>=1 and *P*-adjust <=0.05) were screened using Venn (**c**) and Volcano plots (**d**). KEGG enrichment analyses were conducted on the 660 genes that were differentially expressed exclusively in the ATO + APA group and the Control group (**e**). GO analysis showed that 660 genes were enriched in anti-tumor-related biological processes (**f**). Cluster heatmap illustrated the expression of twelve differential genes that were enriched in the mTOR signaling pathway (**g**). PPI showed that GRB10 was associated with the *VEGFR2/Akt/mTOR* and *Akt/GSK-3β/c-Myc* singling pathways (**h**). Histograms represent the mRNA level of GRB10 after the single drug or combination of ATO and apatinib in H446 and H196 cells for 48 h (**i**) (****, *P* < 0.0001, **, *P* < 0.01 versus the control group)

Principal Component Analysis (PCA) illustrated the distribution of samples that underwent RNA sequencing (Fig. 4a). The genes that differed between Control and ATO + APA groups were analyzed through GESA (Fig. 4b). The findings revealed a positive correlation with the ATO + APA groups and signals associated with cancer, organ system cancer, lung small cell carcinoma, the mTOR signaling pathway, apoptosis, and response to endoplasmic reticulum stress. Differentially expressed genes (|log2FC|>=1 and *P*-adj<=0.05) were screened using the R package “DESeq2” and visualized using Venn and Volcano plots (Fig. 4c, d). Functional enrichment analyses were conducted on the 660 DEGs that were differentially expressed exclusively in the APA + ATO group and the Control group. The top three KEGG pathway analysis showed that 660 DEGs were related to Hippo signaling pathway, TGF-β signaling pathway and mTOR signaling pathway (*P-*adj <=0.05) (Fig. 4e). GO analysis showed that 660 DEGs were enriched in anti-tumor-related biological processes, such as regulation of mononuclear cell migration, cell cycle arrest and positive regulation of extrinsic apoptotic signaling pathway via death domain receptors, and so on (Fig. 4f). Figure 4g illustrated the expression of twelve DEGs that were enriched in the mTOR signaling pathway. It was noteworthy that GRB10 had the smallest adjusted *P* value. The protein-protein interaction networks showed that GRB10 interacted with PTK2B (Supplement 3), which is also known as PKB/Akt, and Akt was activated by PI3K or phosphatidylinositol-dependent kinase (PDK), as well as growth factors, inflammation, and DNA damage, then, the signaling occurred through downstream effectors, such as mTOR, glycogen synthase kinase 3β (GSK-3β), among others. Overexpression or activation of Akt had been observed in many cancers, including ovarian, lung, and pancreatic cancers, and had been associated with increased cancer cell proliferation and survival [14]. Then, we designed an interaction network graph that GRB10 was associated with the VEGFR2/Akt/mTOR and Akt/GSK-3β/c-Myc pathways (Fig. 4h). Our experiment showed that the mRNA level of GRB10 increased significantly after the combination of ATO and apatinib treated in H446 and H196 cells for 48 h, compared with the control group and the single drug group (Fig. 4i).

**Fig. 5:**
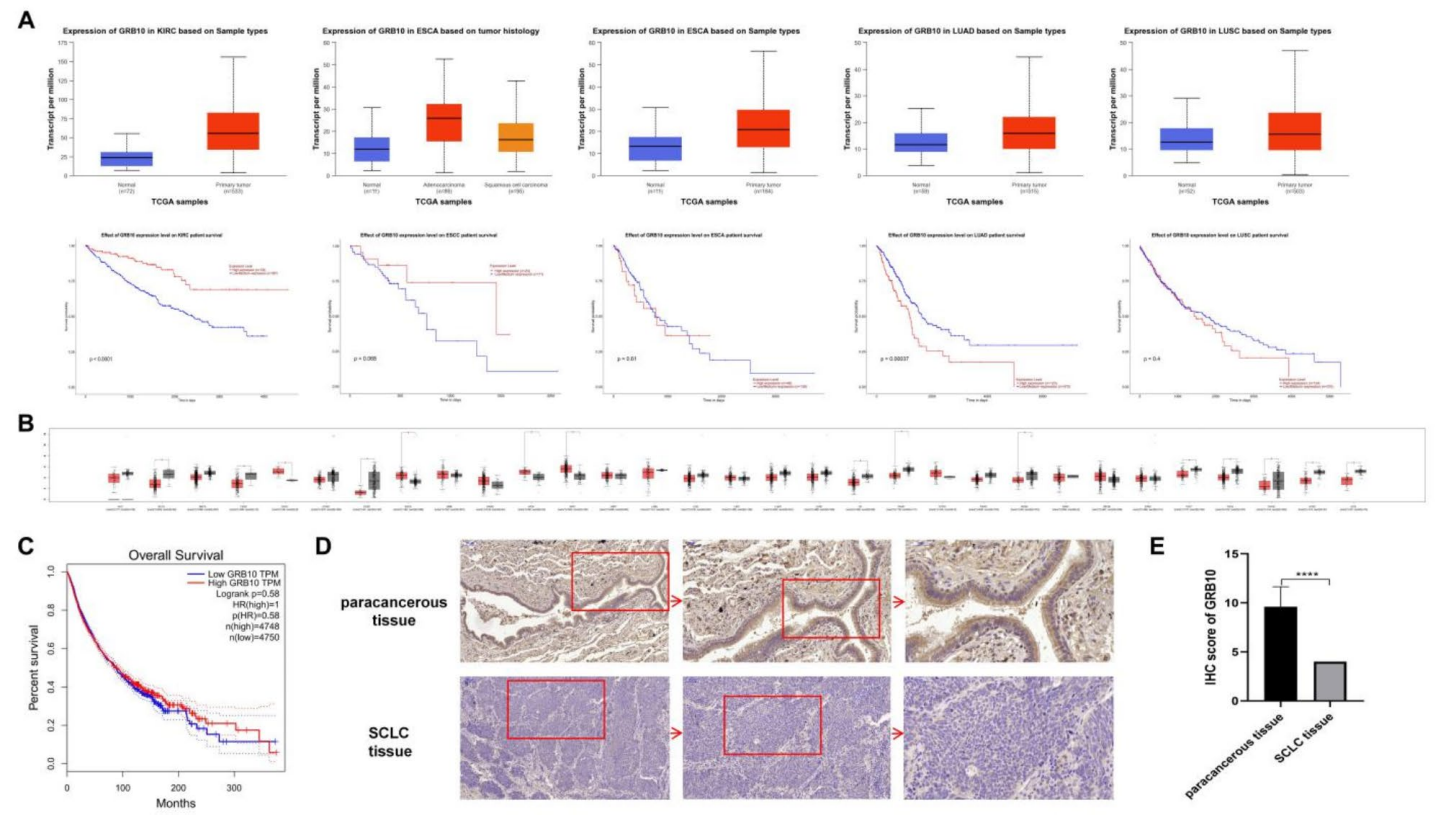
Expression of GRB10 in KIRC, ESCC, LUCA, LUSC and ESCA based on sample types, and effect of GRB10 expression level on KIRC, ESCC, LUCA, LUSC and ESCA patient survival (**a**). Pan-cancer analysis showed that the expression level of GRB10 (**b**) and the Overall Survival (**c**) in different cancer types (**d**) Typical immunohistochemistry of GRB10 in SCLC tissue and adjacent tissue (**e**) IHC score of GRB10 in SCLC tissues and adjacent tissue (****, *P* < 0.0001). KIRC: Kidney renal clear cell carcinoma; ESCC: Esophageal squamous cell carcinoma; LUCA: Lung adenocarcinoma; LUSC: Lung squamous cell carcinoma; ESCA: Esophageal carcinoma

Nevertheless, the mRNA levels of VEGFR2, FN1, AGRN were significantly downregulated compared with the control group and single drug group (Supplement 4). In Fig. 5a, GRB10 was highly expressed in KIRC and ESCC, with a good prognosis, but highly expressed in LUCA, LUSC, and ESCA, with a poor prognosis. The summary results of the TCGA database showed that the expression level of GRB10 varied in different cancer types, and the prognosis was also different (|log2FC|>=1 and *P*-value<=0.01) from GEPIA (http://gepia.cancer-pku.cn/index.html) (Fig. 5b, c). We used immunohistochemistry to evaluate the expression of GRB10 in collected 15 SCLC patients and adjacent tissue specimens. The results showed that the expression of GRB10 in SCLC tissues was significantly lower than that in adjacent tissues (Fig. 5d). Currently, there is no report on the correlation between the expression level of GRB10 and prognosis in SCLC.

### Combination of ATO and APA down-regulated protein molecules in the VEGFR2/Akt/mTOR and Akt/GSK-3β/c-Myc signaling pathway in SCLC cells

**Fig. 6:**
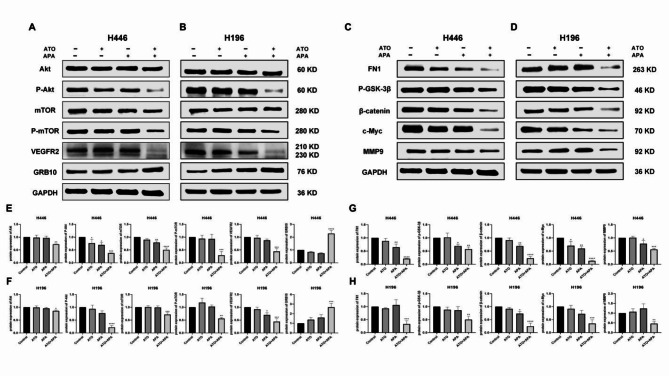
Effect of ATO, apatinib alone and in combination on Akt related pathways after 48 h treatment. H446 cells were treated with 1 µM of ATO, 16 µM of apatinib or 1 µM of ATO and 16 µM of apatinib in combination; H196 cells were treated with 2 µM of ATO, 32 µM of apatinib or 2 µM of ATO and 32 µM of apatinib in combination. Figures are the protein expression of VEGFR2, Akt, P-Akt, mTOR, P-mTOR, GRB10 and GAPDH of H446 (**a**) and H196 (**b**) cells; Figures are the protein expression of FN1, P-GSK-3β, β-catenin, c-Myc, MMP9 and GAPDH of H446 (**c**) and H196 (**d**) cells; Histograms represent the relative intensity of protein of H446 (**e**,** g**) and H196 (**f**,** h**); All data are shown as the mean ± SD of three independent experiments (****, *P* < 0.0001, ***, *P* < 0.001, **, *P* < 0.01, *, *P* < 0.05 versus the control group)

Based on the RNA-sequencing results, the main pathways were screened through KEGG enrichment analysis. Therefore, we validated the Akt related pathways of the combination of ATO and APA in inhibiting SCLC cells (Fig. 6). Western blot showed that, compared with the control group and single drug ATO or APA group, the combination of ATO and APA could down-regulate the protein levels of VEGFR2, P-Akt, P-mTOR, FN1, P-GSK-3β, β-catenin, c-Myc and MMP9 (Fig. 6a, b, c, d). Previous literature reports that GRB10 seems to play a leading role in development by negatively affecting cell proliferation, which overexpression negatively regulated activation of PI3K/Akt signaling through its association with a broad range of signaling molecules [15]. GSK-3β, as a main intracellular serine/threonine family kinase, is an important substrate of Akt [16], which is essential for the stability of β-catenin in the cytoplasm [17]. Importantly, in our research, we found that combination of ATO and APA could up-regulate protein levels of GRB10 (Fig. 6a, b).

### Combination of ATO and APA synergistically inhibited proliferation, migration, and invasion of SCLC Cells by targeting GRB10

**Fig. 7:**
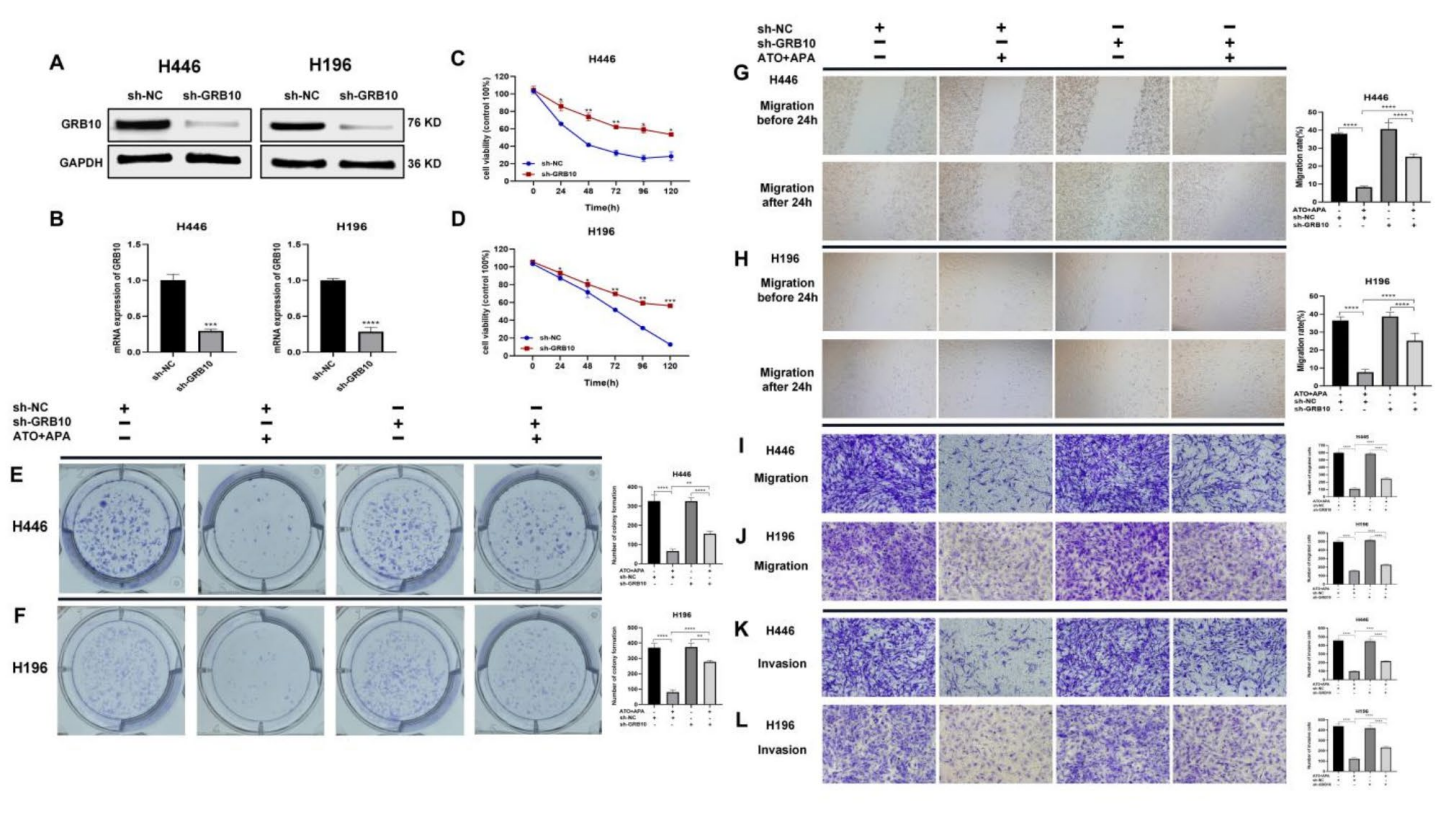
GRB10 knockdown partially reversed the inhibitory effects of the combination of ATO and apatinib on SCLC cell proliferation, migrated and invasive effects. Transfection efficiency and knockdown efficiency of GRB10 were assessed by Western blot (**a**) and qPCR (**b).** CCK-8 assays were used to assess cell proliferation. The proliferation inhibitory effect of combination of ATO (1 µM) and apatinib (16 µM) on the sh-NC-H446 and sh-GRB10-H446 cells for 24, 48, 72, 96, 120 h, respectively (**c**); The proliferation inhibitory effect of combination of ATO (1 µM) and apatinib (16 µM) on the sh-NC-H196 and sh-GRB10-H196 cells for 24, 48, 72, 96, 120 h, respectively (**d**). Colony formation assays were performed to investigate the colony formation ability of SCLC cells. The sh-NC-H446 and sh-GRB10-H446 cells were treated with combination of ATO (1 µM) and apatinib (16 µM) (**e**); The sh-NC-H196 and sh-GRB10-H196 cells were treated with combination of ATO (2 µM) and apatinib (32 µM) (**f**); Typical images of wound healing showed that sh-NC-H446 and sh-GRB10-H446 cells were treated with the combination of ATO (1 µM) and apatinib (16 µM) for 24 h (**g**); Sh-NC-H196 and sh-GRB10-H196 cells were treated with the combination ATO (2 µM) and apatinib (32 µM) for 24 h (**h**); Transwell assay showed that sh-NC-H446 and sh-GRB10-H446 cells were treated with the combination of ATO (1 µM) and apatinib (16 µM) for 48 h (**i**,** k**), Sh-NC-H196 and sh-GRB10-H196 cells were treated with the combination ATO (2 µM) and apatinib (32 µM) for 48 h (**j**,** l**) (magnification, ×100; scale bars, 200 μm); Histograms showed the amount of colony formation, migrated and invasive cells. All data are shown as the mean ± SD of three independent experiments (****, *P* < 0.0001, ***, *P* < 0.001, **, *P* < 0.01, *, *P* < 0.05)

To determine whether GRB10 was involved in the cytotoxicity of the combination of ATO and APA, we constructed a stable sh-GRB10-H446 and sh-GRB10-H196 cell line, respectively. Transfection efficiency and knockdown efficiency were assessed by Western blot (Fig. 7a) and qPCR (Fig. 7b). Subsequently, the effect of combination of ATO and APA on cell proliferation was assessed using CCK-8 and colony formation assays in sh-GRB10-H446 and sh-GRB10-H196 cells. The CCK-8 assay showed that combination of ATO and APA reduced viability of cells, while GRB10 knockdown partially reversed the toxic effects of combination of ATO and APA, indicating that GRB10 was involved in the toxic effects of combination of ATO and APA on cells (Fig. 7c, d). Consistent with the results from the CCK-8 assays, the colony formation assay also showed the same conclusion (Fig. 7e, f). Wound healing assays showed that the migration area on sh-GRB10-H446 cells was significantly larger than that on sh-NC-H446 cells in combination group after 24 h (Fig. 7g). The result was also consistent on the sh-GRB10-H196 cells (Fig. 7h). Then, transwell assays showed that the combination group on sh-NC-H446 or sh-NC-H196 cells had the least number of migrated and invasive cells compared with the combination group on sh-GRB10-H446 (Fig. 7i, k) or sh-GRB10-H196 cells (Fig. 7j, l). Collectively, the data demonstrated that combination of ATO and APA exerted a growth inhibitory and invasive effect on SCLC *via* upregulation of GRB10.

### Combination of ATO and APA synergistically induced apoptosis of SCLC cells by targeting GRB10

**Fig. 8:**
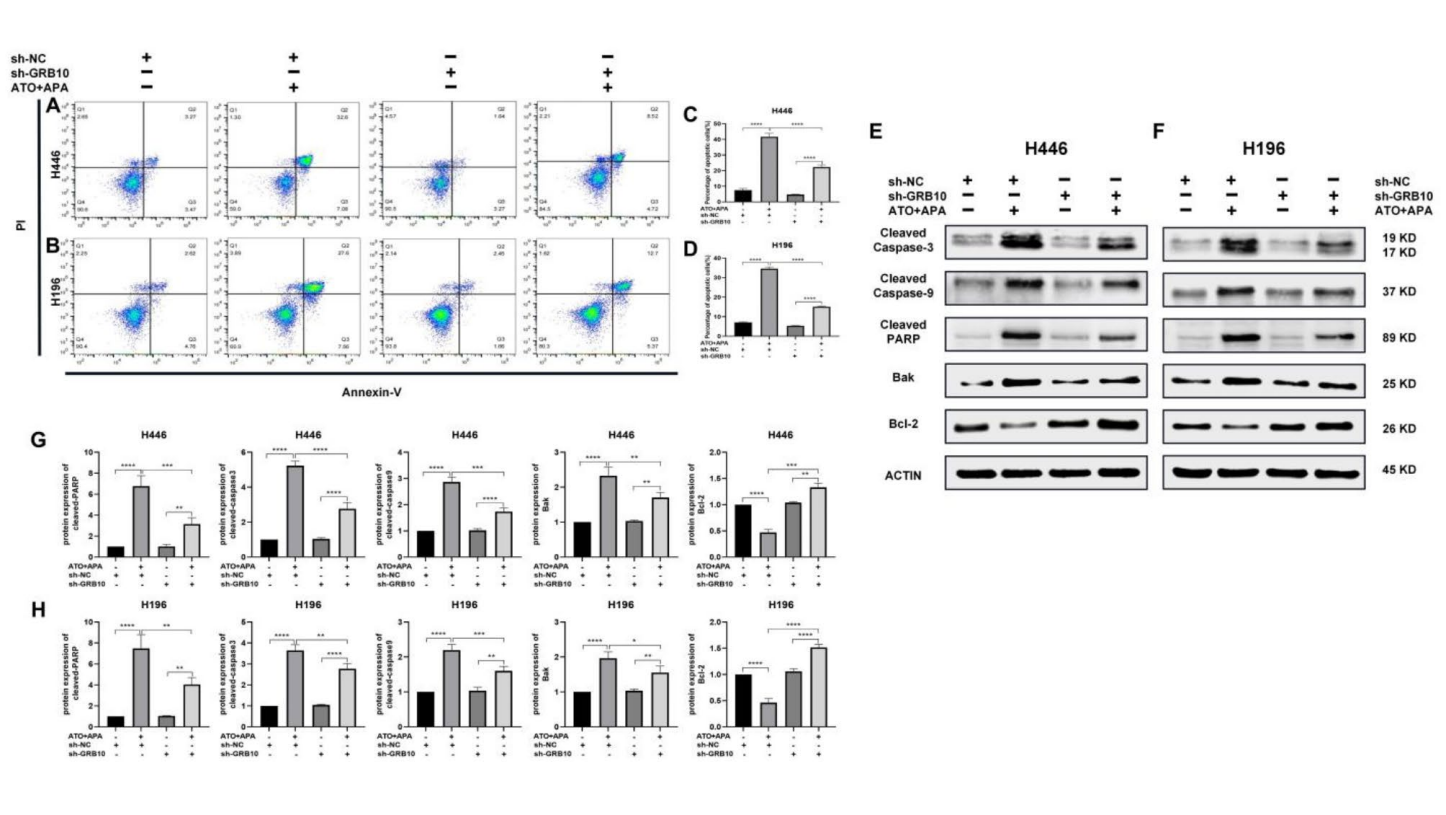
GRB10 knockdown reduced the appotosis effects of the combination of ATO and apatinib on SCLC cells. Representative profiles showing apoptosis in sh-NC-H446 and sh-GRB10-H446 cells were treated with the combination of ATO (1 µM) and apatinib (16 µM) for 48 h (**a**); Sh-NC-H196 and sh-GRB10-H196 cells were treated with the combination ATO (2 µM) and apatinib (32 µM) for 48 h (**b**); Histograms show the average apoptosis rate (**c**,** d**). Then, western blot was used to evaluate the levels of apoptosis-related proteins in sh-H446 (**e**) and sh-H196 (**f**) cells; Relative intensity of protein on apoptosis signaling pathway in sh-H446 (**g**) and sh-H196 (**h**) cells. All data are shown as the mean ± SD of three independent experiments (****, *P* < 0.0001, ***, *P* < 0.001, **, *P* < 0.01,, *P < 0.05)

To investigate whether ATO and APA synergistically induced the apoptosis of SCLC cells by targeting GRB10, flow cytometry was used to measure cell apoptotic rate. Western blot was used to measure pro- and anti-apoptosis-related proteins, respectively. As shown in Fig. 8a and b, treatment with combination of ATO and APA for 48 h significantly increased the apoptotic rate of sh-NC-H446 and sh-NC-H196 cells, whereas GRB10 knockdown significantly reduced pro-apoptotic effect of the combination of ATO and APA on SCLC cells. Furthermore, Western blot showed that the combination of ATO and APA treatment notably increased the levels of Cleaved Caspase-3, Cleaved Caspase-9, Cleaved PARP and Bax in the cytoplasm, and decreased Bcl-2 expression in sh-NC cells, but these effects were attenuated by sh-GRB10 (Fig. 8e, f). These results suggested that knockdown of GRB10 attenuated the pro-apoptotic effects of combination of ATO and APA by maintaining mitochondrial function and inhibiting the Caspase cascade. Together, these data supported the notion that the combination of ATO and APA induced apoptosis of SCLC cells *via* upregulation of GRB10.

### Combination of ATO and APA down-regulated protein molecules in the VEGFR2/Akt/mTOR and Akt/GSK-3β/c-Myc signaling pathway in SCLC Cells by targeting GRB10

**Fig. 9:**
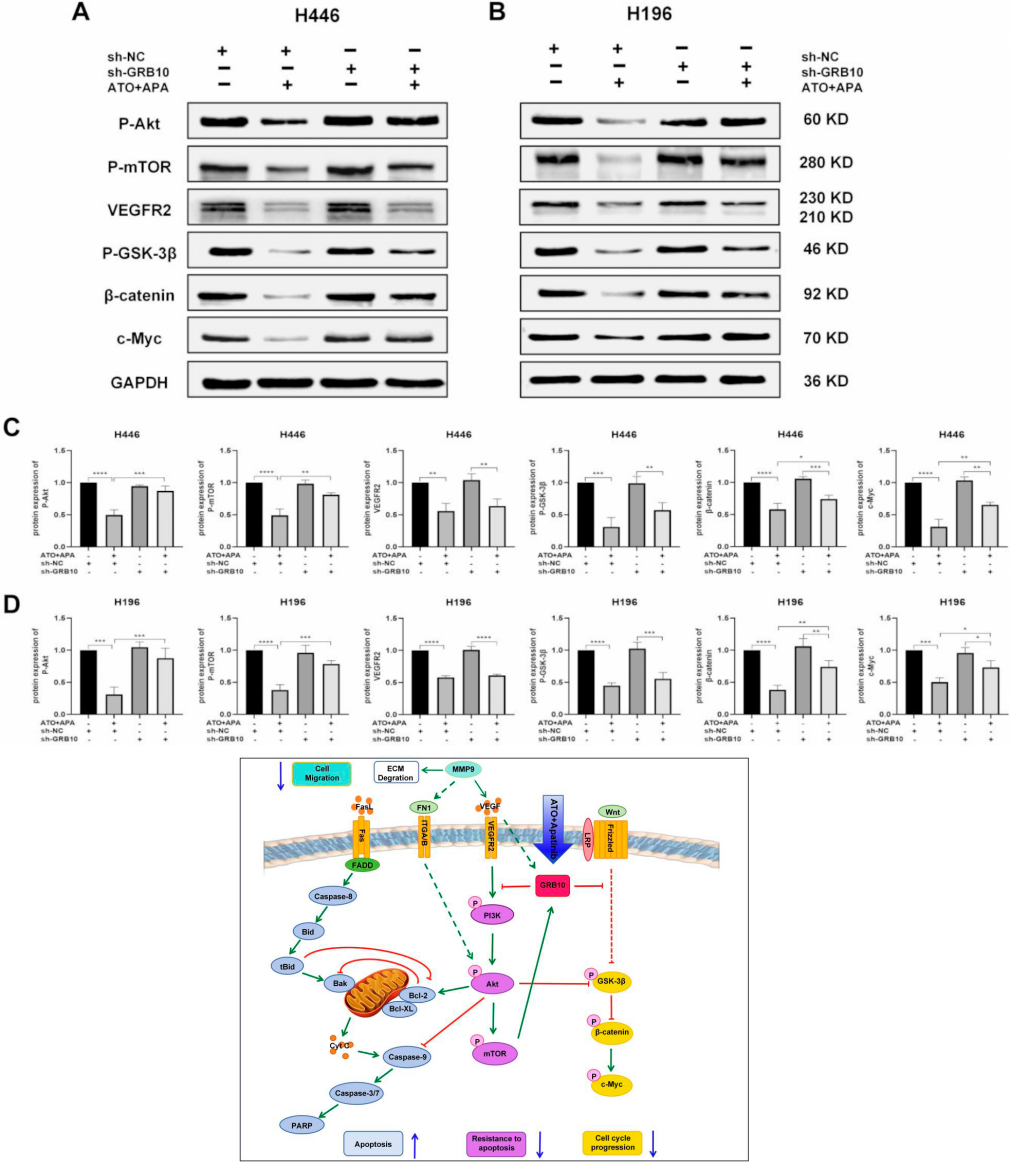
GRB10 knockdown partially reversed the expression of Akt related pathway proteins of the combination of ATO and apatinib on SCLC cells. Sh-H446 cells were treated with the combination of ATO (1 µM) and apatinib (16 µM) for 48 h; Sh-H196 cells were treated with the combination ATO (2 µM) and apatinib (32 µM) for 48 h; Figures are the protein expression of VEGFR2, P-Akt, P-mTOR, P-GSK-3β, β-catenin and MMP9 of sh-H446 (**a**) and sh-H196 (**b**) cells; Histograms represent the relative intensity of protein of sh-H446 (**c**) and sh-H196 (**d**); All data are shown as the mean ± SD of three independent experiments (****, *P* < 0.0001, ***, *P* < 0.001, **, *P* < 0.01, *, *P* < 0.05). GRB10 mainly exerts its anti-tumor effects through inhibiting the *VEGFR2/Akt/mTOR* and *Akt/GSK-3β/c-Myc* signaling pathway in SCLC. Obviously, akt is a key signal pathway in the mechanism, which can regulate the Caspase/PARP cascade to resist apoptosis, and also regulate the *GSK-3β/β-catenin/c-Myc* signaling pathway to affect cell cycle progression. When the combination of ATO and apatinib in SCLC, which could enhance GRB10 expression and then exert anti-tumor effects by inducing apoptosis, inhibiting invasion and metastasis, and interfering with cell cycle progression

To elucidate the mechanism underlying the effects of GRB10 in the combination of ATO and APA in SCLC cells, we investigated the protein expression of VEGFR2, P-Akt, P-mTOR, P-GSK-3β, β-catenin and c-Myc in the sh-GRB10 cells. As expected, the protein expression levels of P-Akt, P-mTOR, P-GSK-3β, β-catenin and c-Myc were partially reversed by knocking down of GRB10 (Fig. 9a, b). Since APA was a specific TKI for VEGFR2, sh-GRB10 had no significant effect on the protein expression level of VEGFR2, suggesting that GRB10 might be a downstream target of VEGFR2. Collectively, Western blot data demonstrated that the combination of ATO and APA down-regulated protein molecules in the VEGFR2/Akt/mTOR and Akt/GSK-3β/c-Myc signaling pathway in SCLC cells by targeting GRB10. See proposed schematic in Fig. 9 for function of GRB10 in regulating signaling pathways of SCLC.

## Discussion

We have made our assessments using in vitro and in vivo models. The in vitro assessment has been made using two cell lines, namely H446 and H196. Both are patient derived cell lines which are the most predominant methods for studying cancer biology. The in vivo assessments have been made using xenograft mouse models with acknowledgement of the limitations of mouse models in SCLC. It has previously been shown that ATO [18–20] and APA [21, 22], independently, inhibit angiogenic activity in cancer stem cells (CSCs) of SCLC. While ATO mediates its action by down regulating stem-cell maintenance factors [18]; APA inhibits vascular endothelial growth factor receptor-2 (VEGFR2) [13].

In the current study we found that the combination of ATO and APA, significantly inhibited cell viability, proliferation, and metastasis as compared to control or the drugs independently. Further we found that in combination, the drugs induced significantly higher percentage of G1 phase cell cycle arrest. The above results demonstrated that the combination of ATO and APA could synergistically enhance the cycle arrest. Similar results have been observed with the drugs in other cancers and in SCLC.

It is well known that the crucial G1 checkpoint is controlled by the retinoblastoma tumor suppressor gene product (*Rb*), which is deficient in SCLC [4]. This de-regulation of *Rb* in SCLC is known to be mediated by Cyclin dependent kinases and Cyclin proteins. In pro myelocytic leukemia, it has been shown that ATO may be mediating demethylation of cyclin proteins accelerating G1 and S transition into the G2/M cell cycle arrest, our results do not indicate the acceleration into G2/M phase [23]. Thus, indicating that arrested cells may be undergoing programmed cell death.

Western blot analysis upregulated the expression of Cleaved Caspase-3, -7, Bak and decreased expression of Bcl-2. Activity of caspase-3 was confirmed by demonstrating the cleavage of its downstream target poly (ADP)-ribose polymerase (PARP) concurrent with findings in other cancers namely, NSCLC, colonic-, breast-, and pancreatic- cancer cells [24–26].

The use of bioinformatic tools, further increased the understanding of the molecular mechanisms driving the cell death pathways mediated by ATO and APA. Amongst the differentially expressed genes, significant upregulation was associated with GRB10. GRB10 is a member of the adaptor protein superfamily, which lacks intrinsic enzyme activity. GRB10 has most often been studied for its pro-proliferative role in different cancers; increased expression of GRB10 being associated with tumorigenesis [27–29]. However, the tumor suppressive role of GRB10 has been elucidated in human acute myeloid leukemia [30]. The study showed that loss of GRB10 resulted in activation of the PI3K/Akt pathway [31]. A comprehensive meta-analysis of published microarray data revealed that the abundance of GRB10 was decreased in many tumor types compared to that in normal tissue counterparts. This was consistent with our immunohistochemistry results performed in the surgical pathology section of the patient, where the expression level of GRB10 was lower in SCLC tissues than in adjacent tissues. GRB10 is well known as a negative regulator of insulin signaling. Our study is the preliminary report demonstrating the role of GRB10 as a tumor suppressor in SCLC.

Consistent with our current findings, a proteomic analysis undertaken by Hsu et al., indicates that GRB10 was stabilized by mTOR mediated phosphorylation consequently leading to a feedback inhibition of PI3K/Akt signaling [32]. In macrophages, it has been shown that ATO induces reactive oxygen species (ROS) causing inhibition of PI3K/Akt and mTOR pathways [32]. It is likely that in SCLC, similar mechanism may lead to inactivation of mTOR pathway eventually leading to stabilization of GRB10. It has also been shown previously that when GRB10 knock down cells were treated with staurosporine and etoposide, it resulted in reduced Caspase-3 [31]. Thus, indicating that the stabilization of GRB10 may take away from the cell, a survival advantage.

Our results thus suggest that in SCLC, GRB10 may thus act as a tumor suppressor and one of the ways it may be acting is through the regulation of *Wnt* signaling pathway. Degradation of the transcriptional co-activator β-catenin is an important regulatory step of the pathway. It has been suggested that binding of *Wnt* to the LRP5/6 family transmembrane protein stabilizes β-catenin [33]. This is achieved by phosphorylation of LRP6 by *Wnt*. This phosphorylation promotes formation of Axin-LRP6 complex. Unavailability of free Axin prevents degradation of β-catenin [34]. Stabilized β-catenin thus drives transcriptional activity *via* complex formation with TCF/Lef family. Consistent with the above findings our results indicate that stabilization of GRB10 enhances degradation of β-catenin. This downregulates its transcriptional activity affecting important cellular processes. It has also been shown elsewhere that GRB10 regulates β-catenin *via* binding to intracellular portion of LRP6 thereby reducing TCF dependent reporter activities [35]. The data also points to the utility of stabilizing GRB10 as a prospective target in SCLC. Since access to patient specimens in SCLC is a challenge, the current study could not confirm the postulated hypothesis with patient data. However, a real time analysis of the same would help establish the conjecture.

## Conclusions

In conclusion, The major findings of the current study are as follows (a) Arsenic trioxide (ATO) and Apatinib (APA) synergistically inhibit cell proliferation, invasion, and migration in SCLC. (b) ATO and APA inhibit cancer progression *via* modulation of *VEGFR2/Akt/mTOR* and *Akt/GSK-3β/c-Myc* signaling pathway. (c) Synergistic action of APA and APA significantly stabilize the expression of GRB10 to play an anti-tumor effect. This study provides a rational of using the combination of arsenic trioxide and apatinib as a promising strategy for the treatment of SCLC.

### Electronic supplementary material

Below is the link to the electronic supplementary material.

Supplementary Material 1

Supplementary Material 2

Supplementary Material 3

Supplementary Material 4

## Data Availability

No datasets were generated or analysed during the current study.
