# Supplementary material for: Combination of arsenic trioxide and apatinib synergistically inhibits small cell lung cancer by down-regulating VEGFR2/mTOR and Akt/c-Myc signaling pathway via GRB10
Source: Hereditas. 2024 Sep 2;161:29. doi: 10.1186/s41065-024-00330-2 (PMC11367874; doi:10.1186/s41065-024-00330-2)
Supplement: Supplementary file 1 — Supplementary Material 1 [file 41065_2024_330_MOESM1_ESM.pdf]

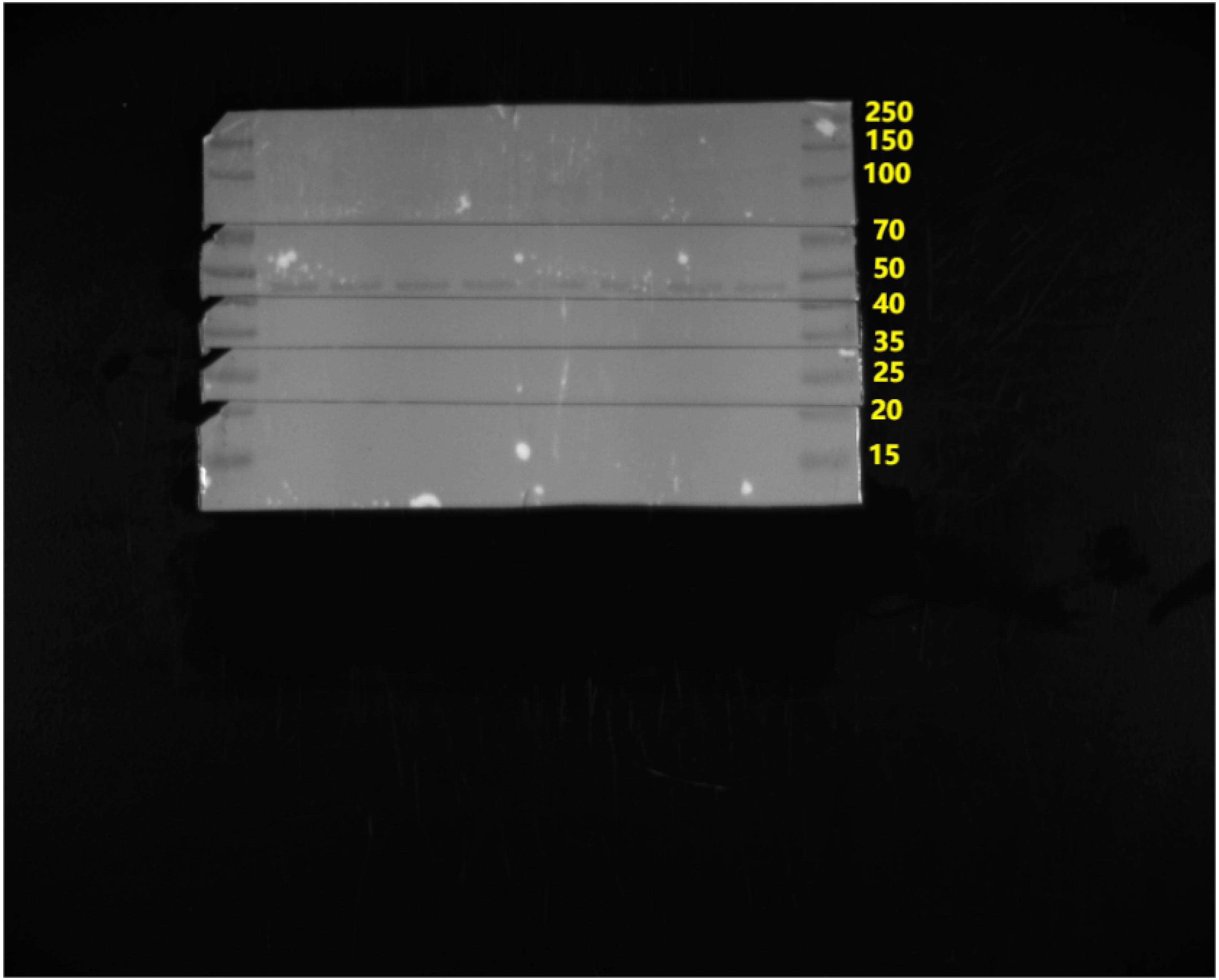
250
150
100
70
50
40
35
25
20
15

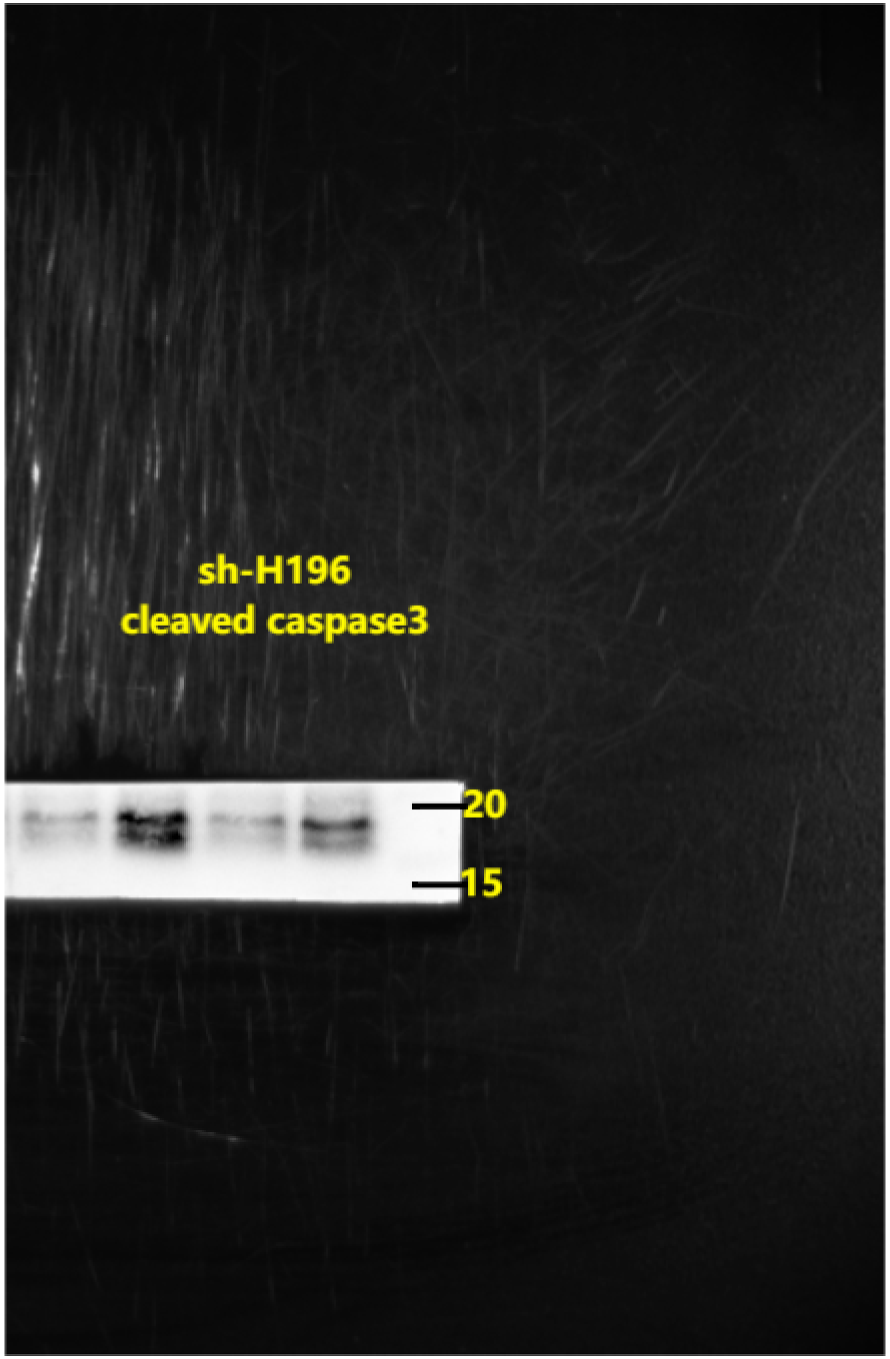
sh-H196
cleaved caspase3
20
15

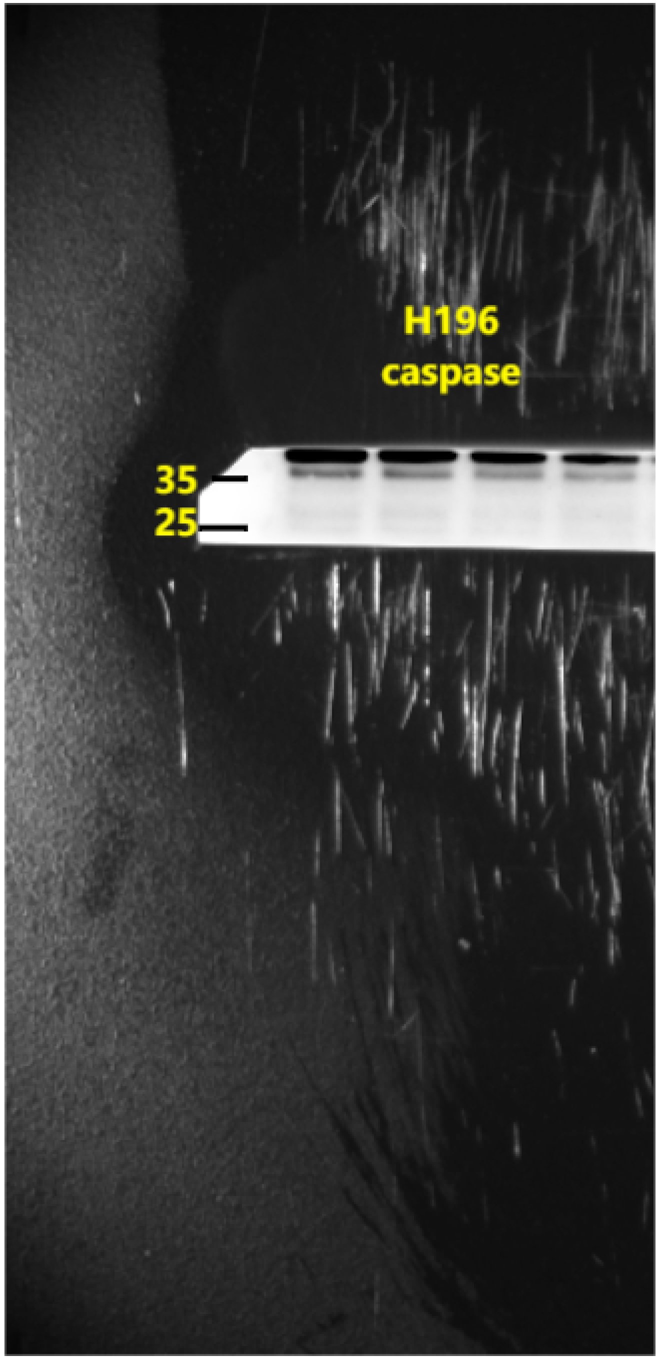
H196
caspase
35
25

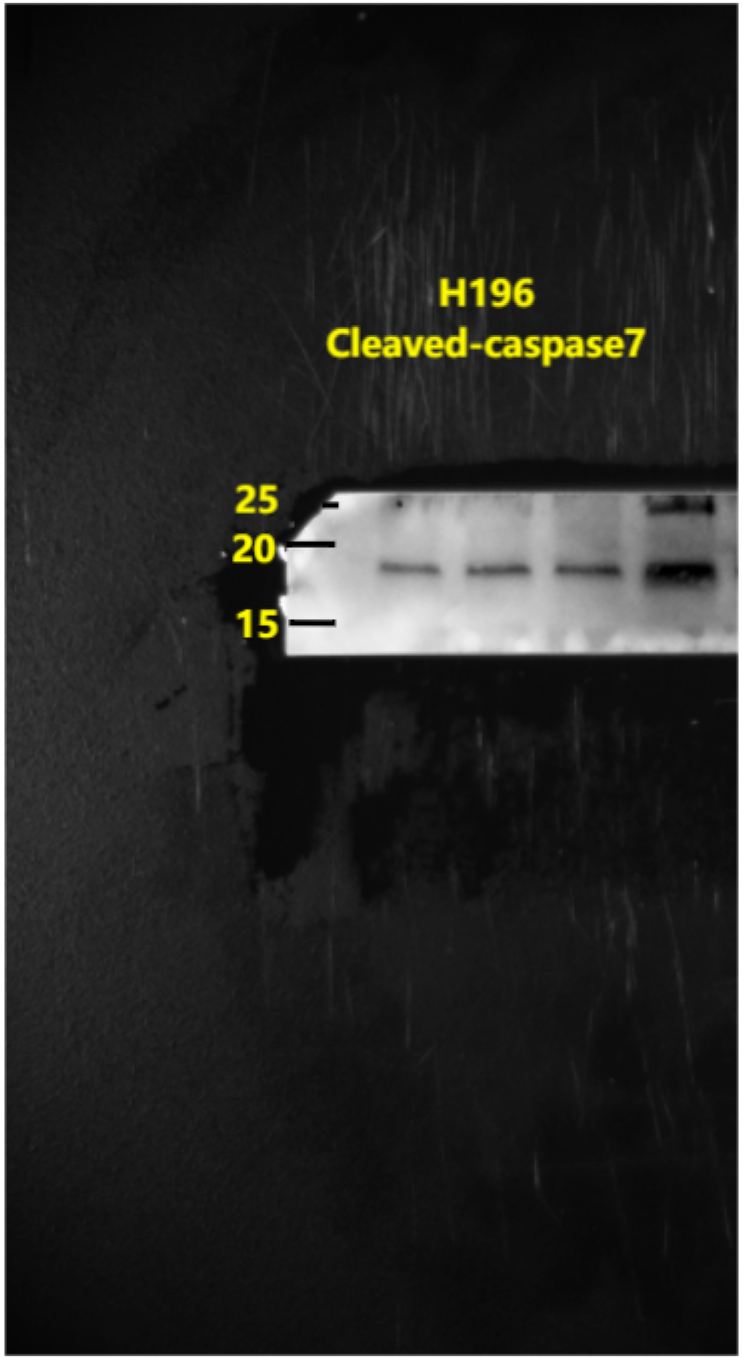
H196
Cleaved-caspase7
25
20
15

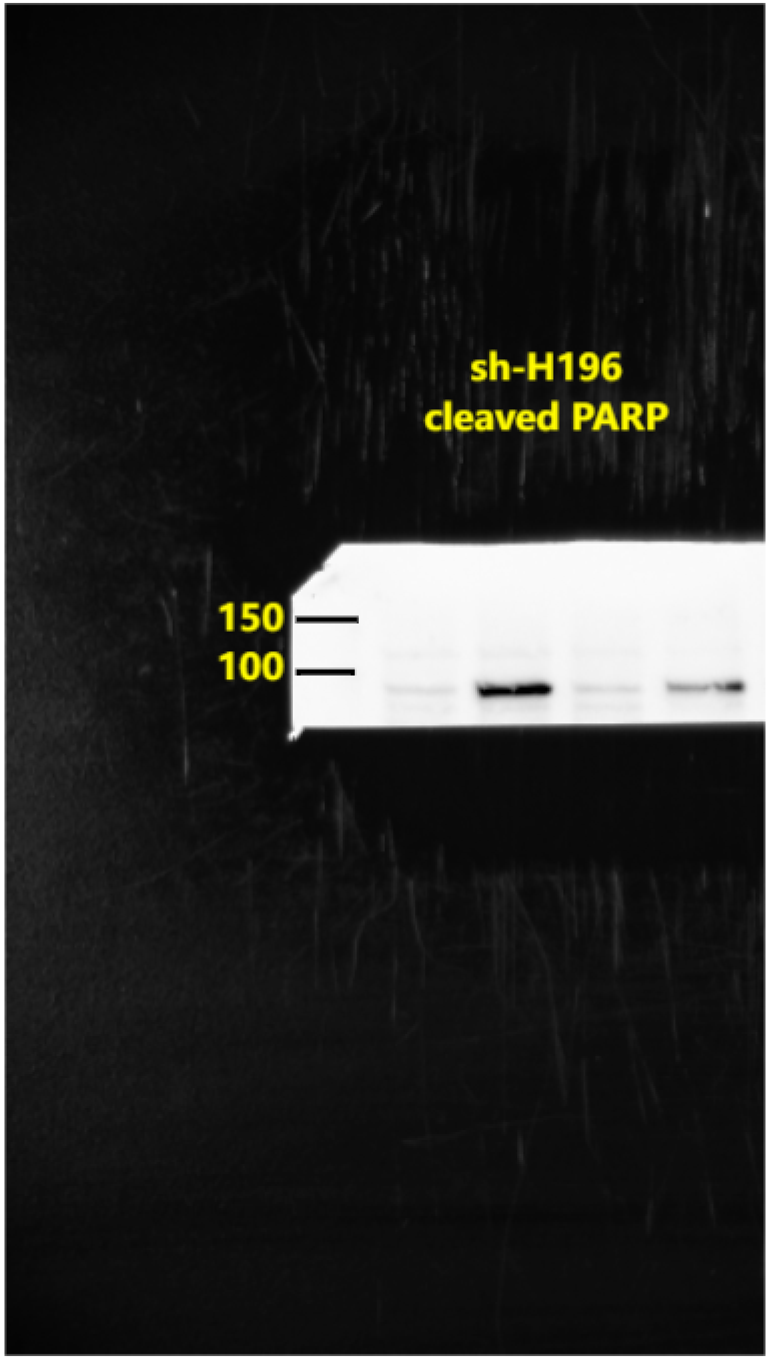
sh-H196
cleaved PARP
150
100

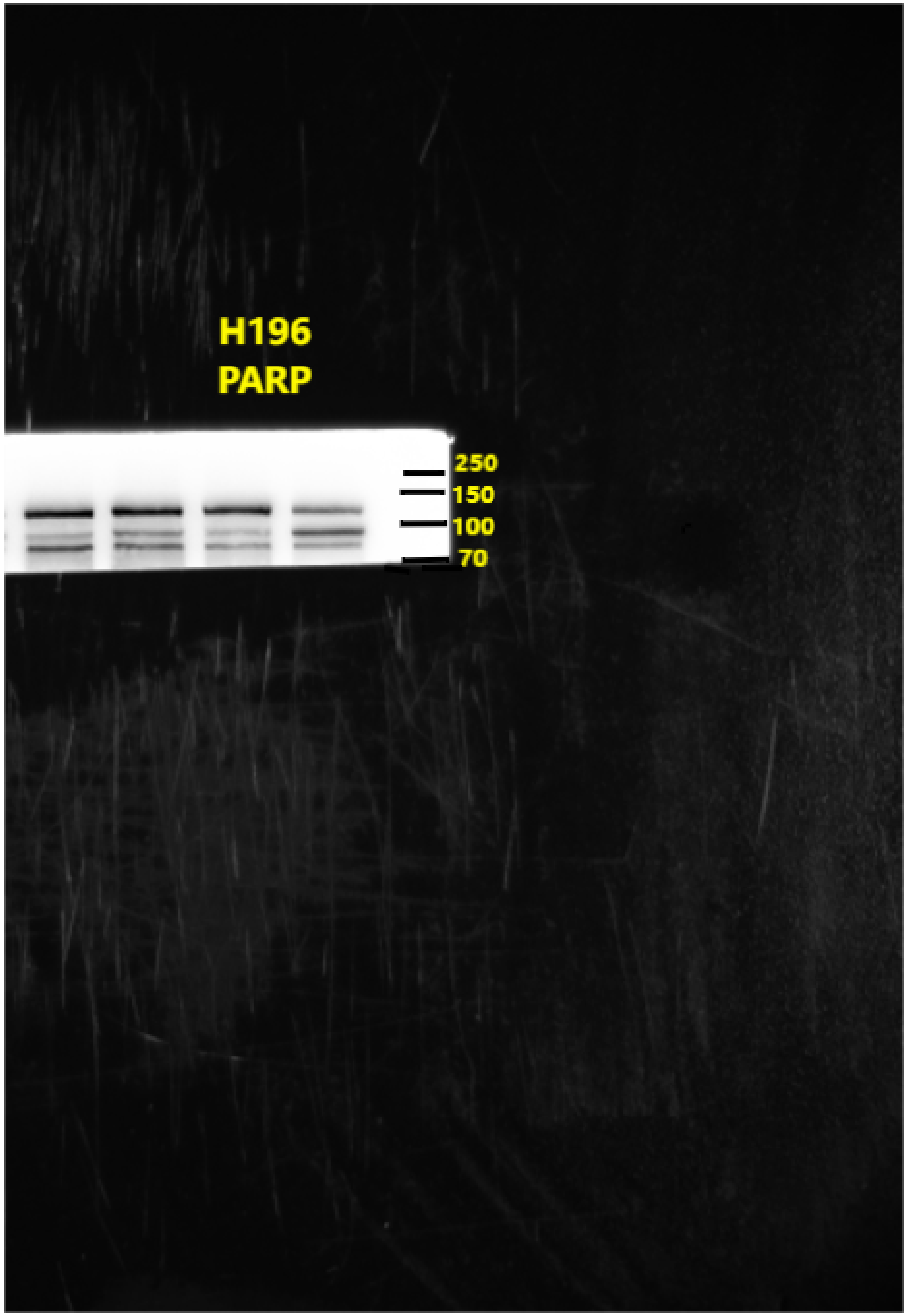
H196
PARP
250
150
100
70

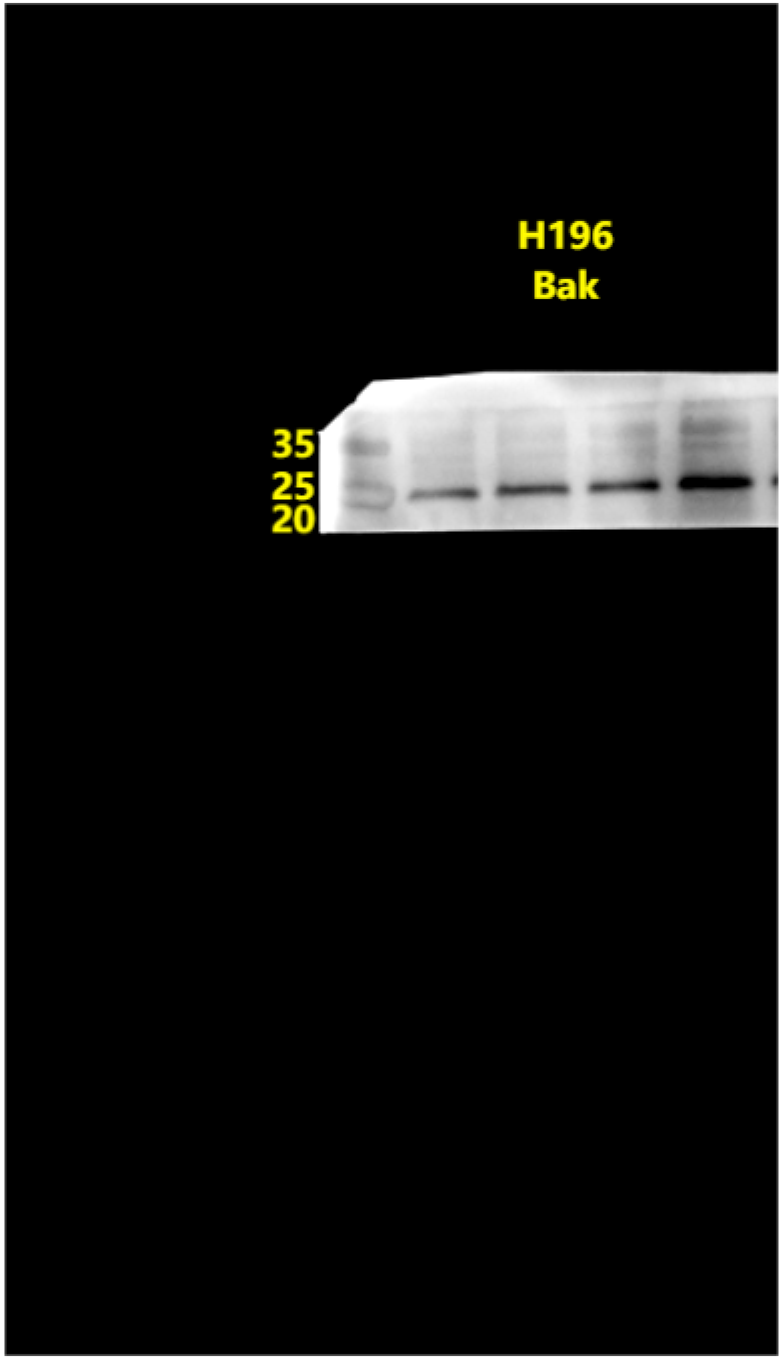
H196
Bak
35
25
20

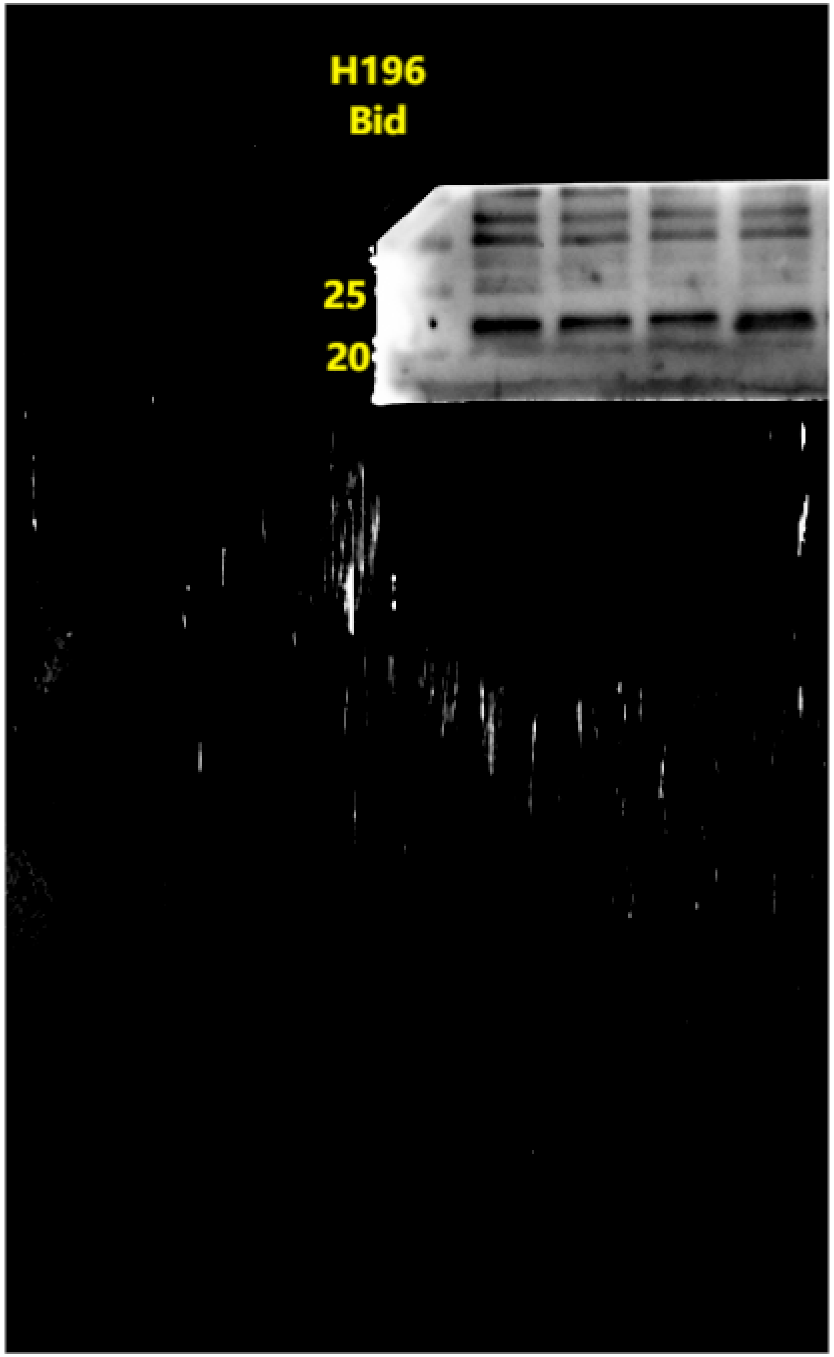
H196
Bid
25
20

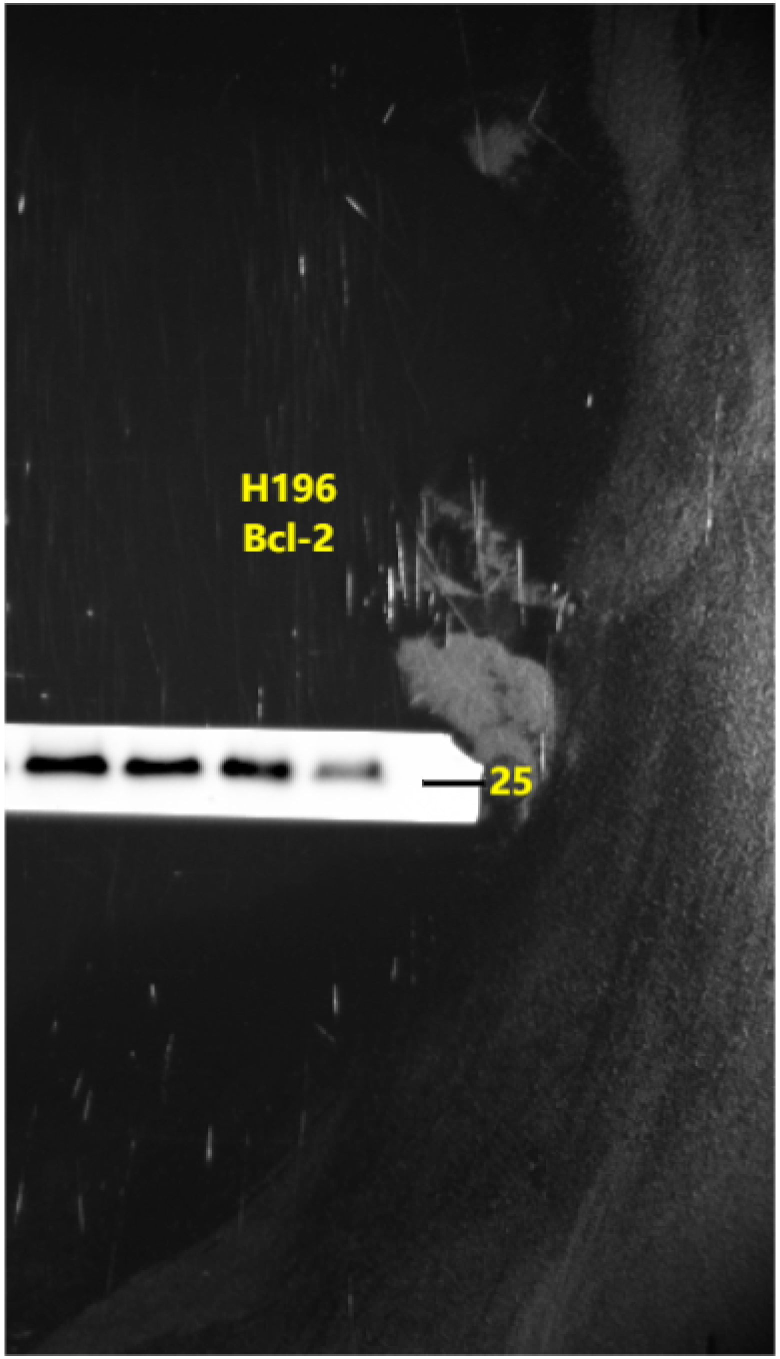
H196
Bcl-2
25

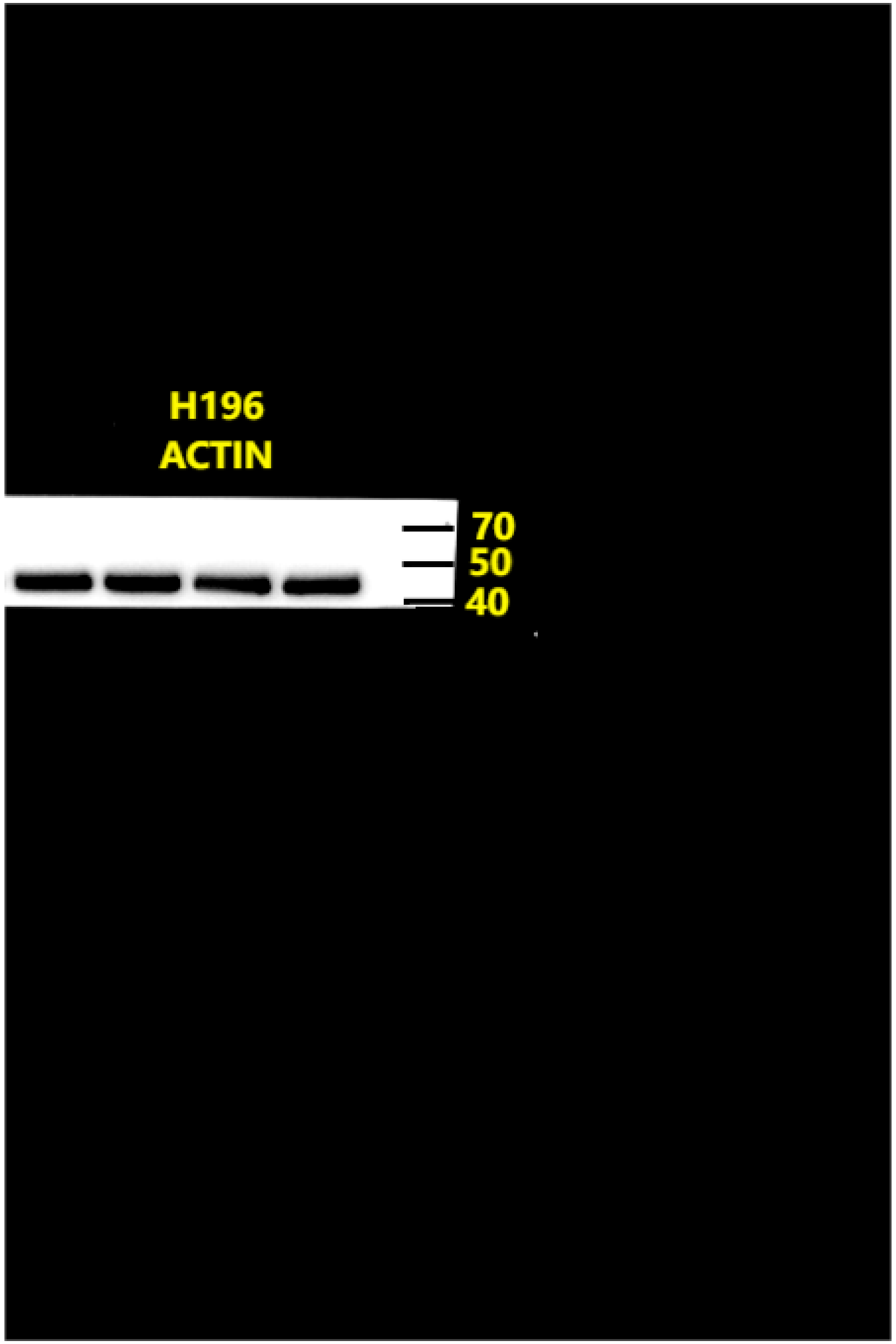
H196
ACTIN
70
50
40

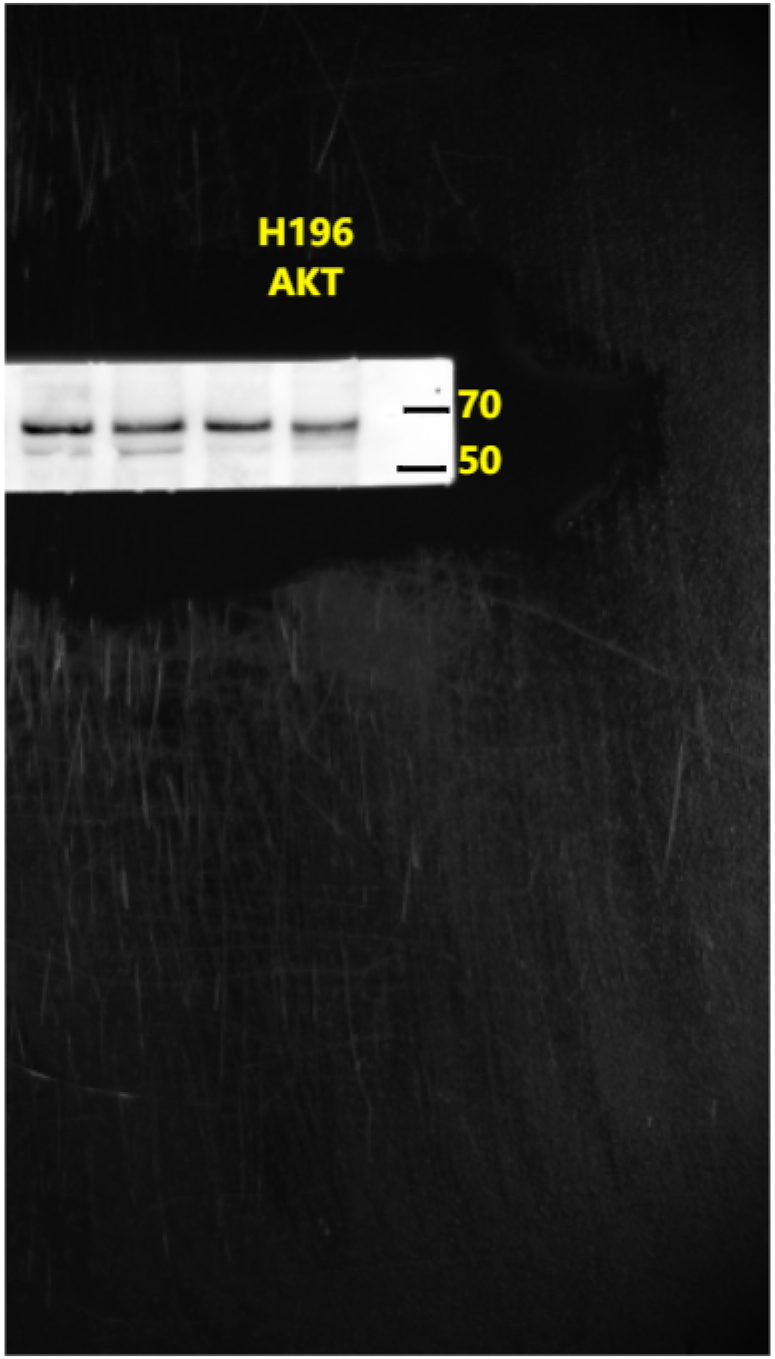
H196
AKT
70
50

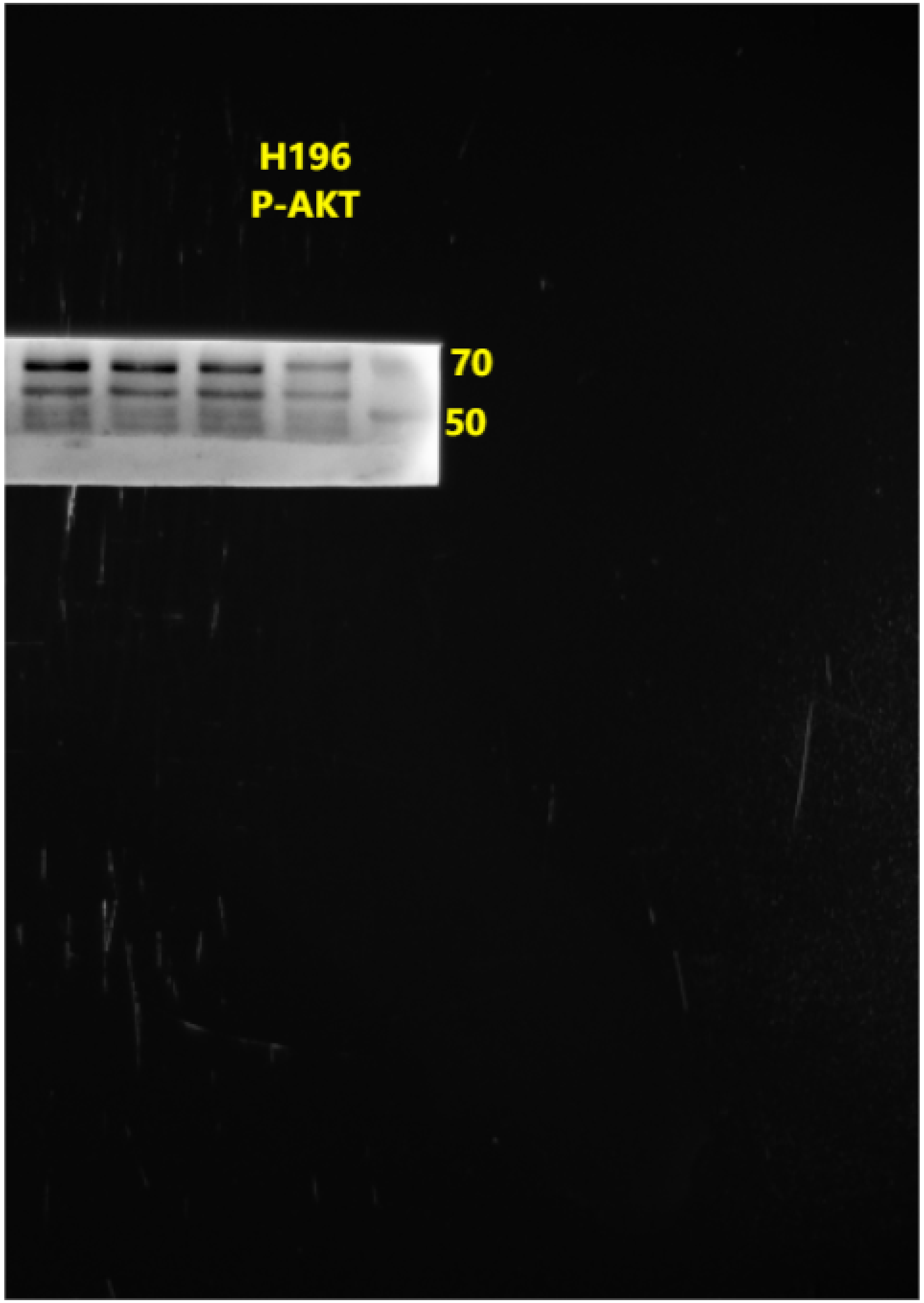
H196
P-AKT
70
50

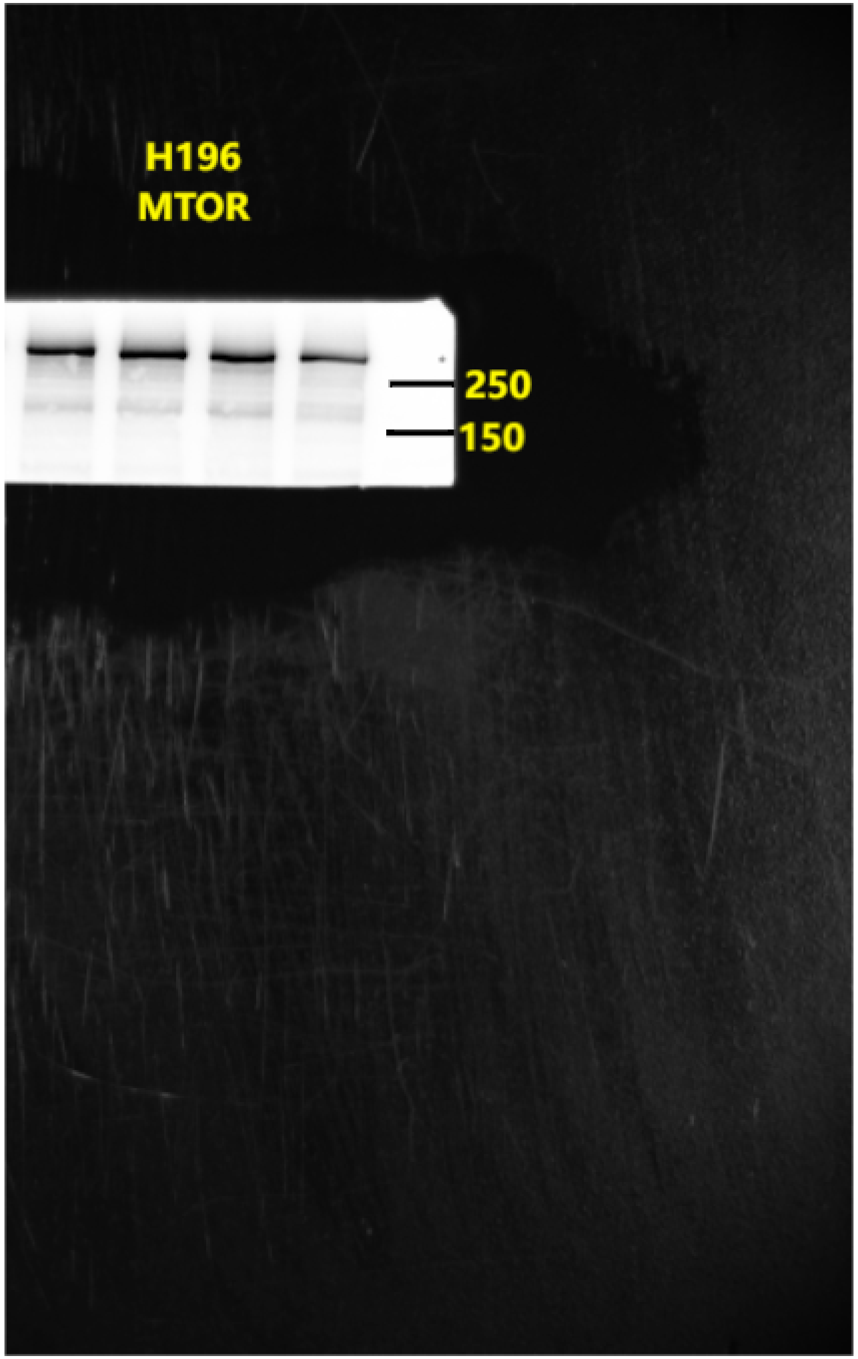
H196
MTOR
250
150

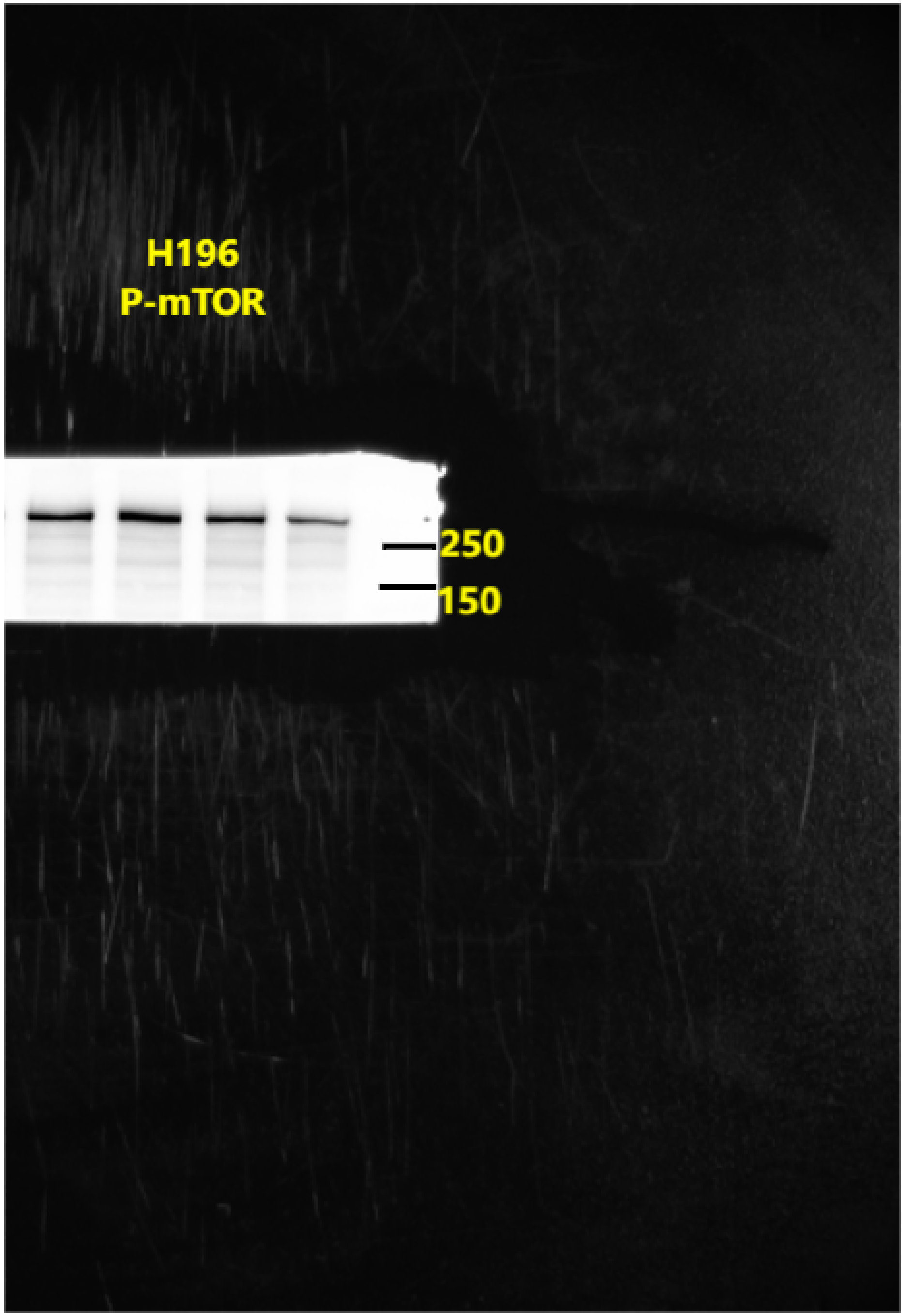
H196
P-mTOR
250
150

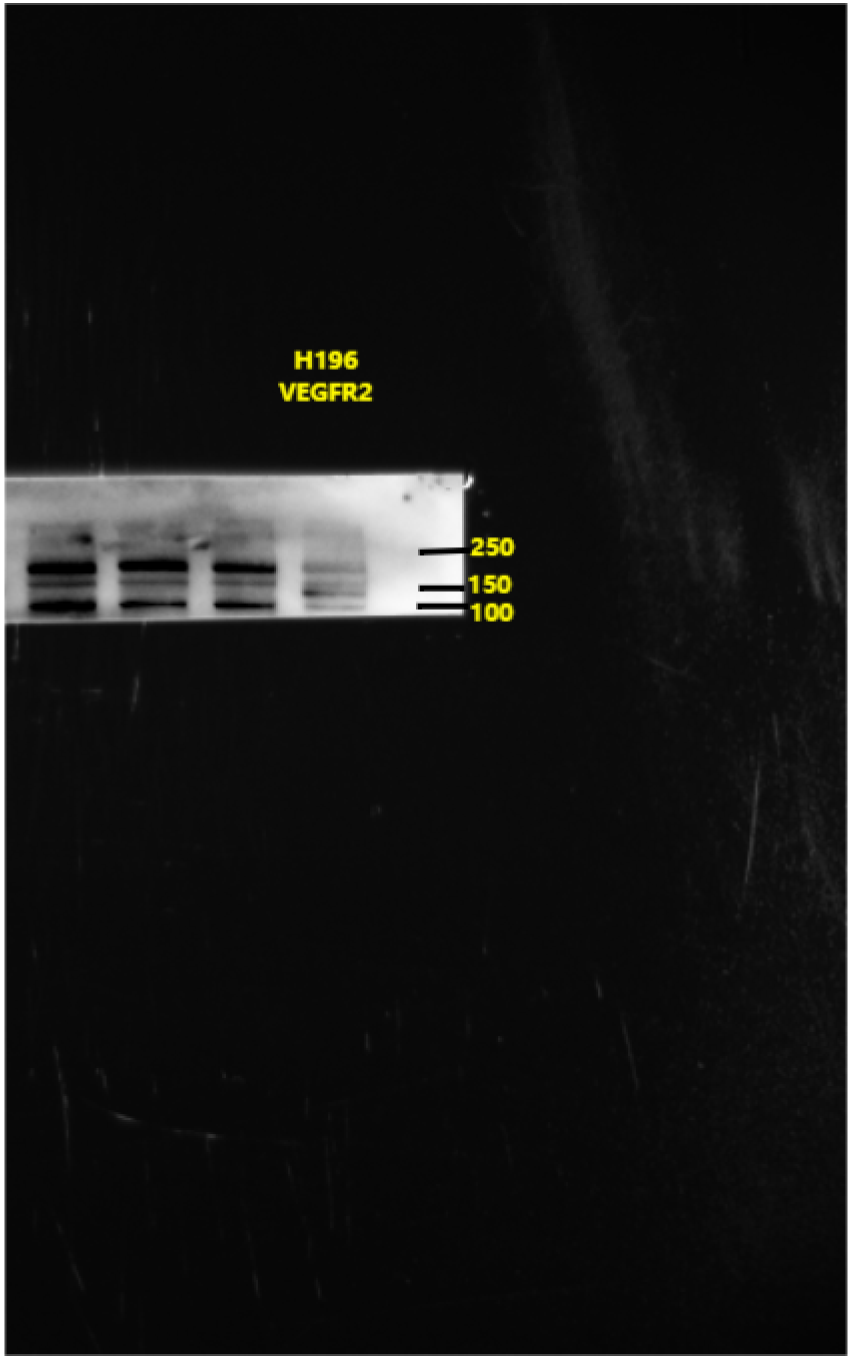
H196
VEGFR2
250
150
100

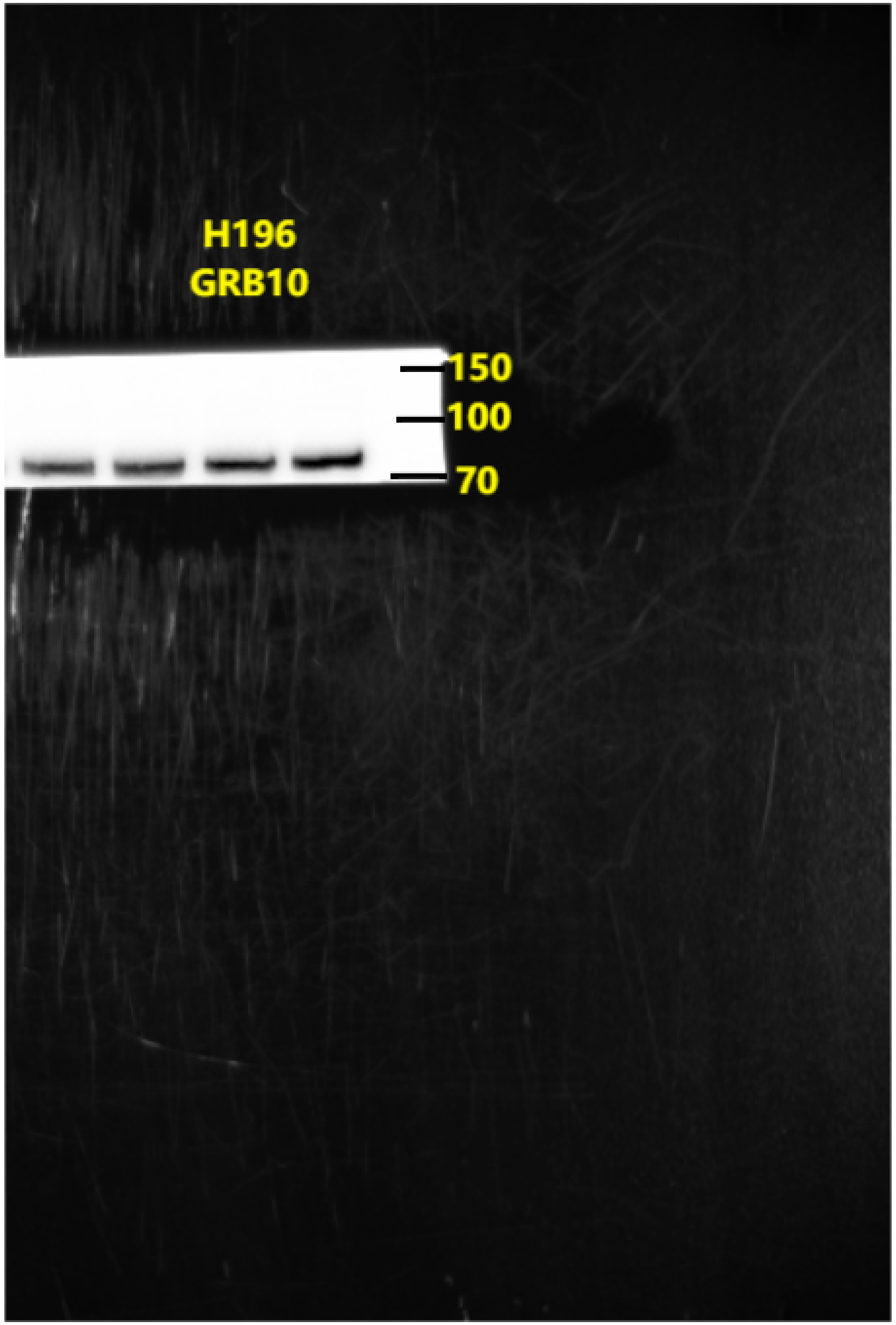
H196
GRB10
150
100
70

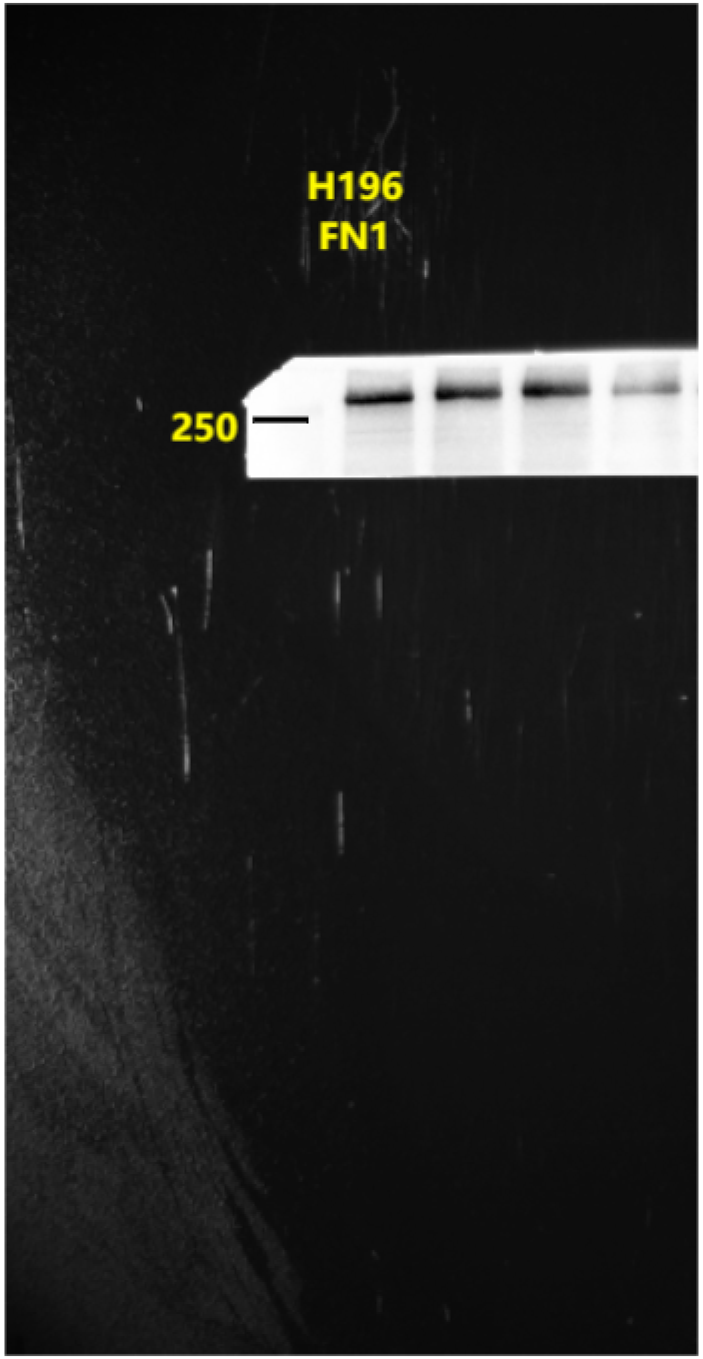
H196
FN1
250

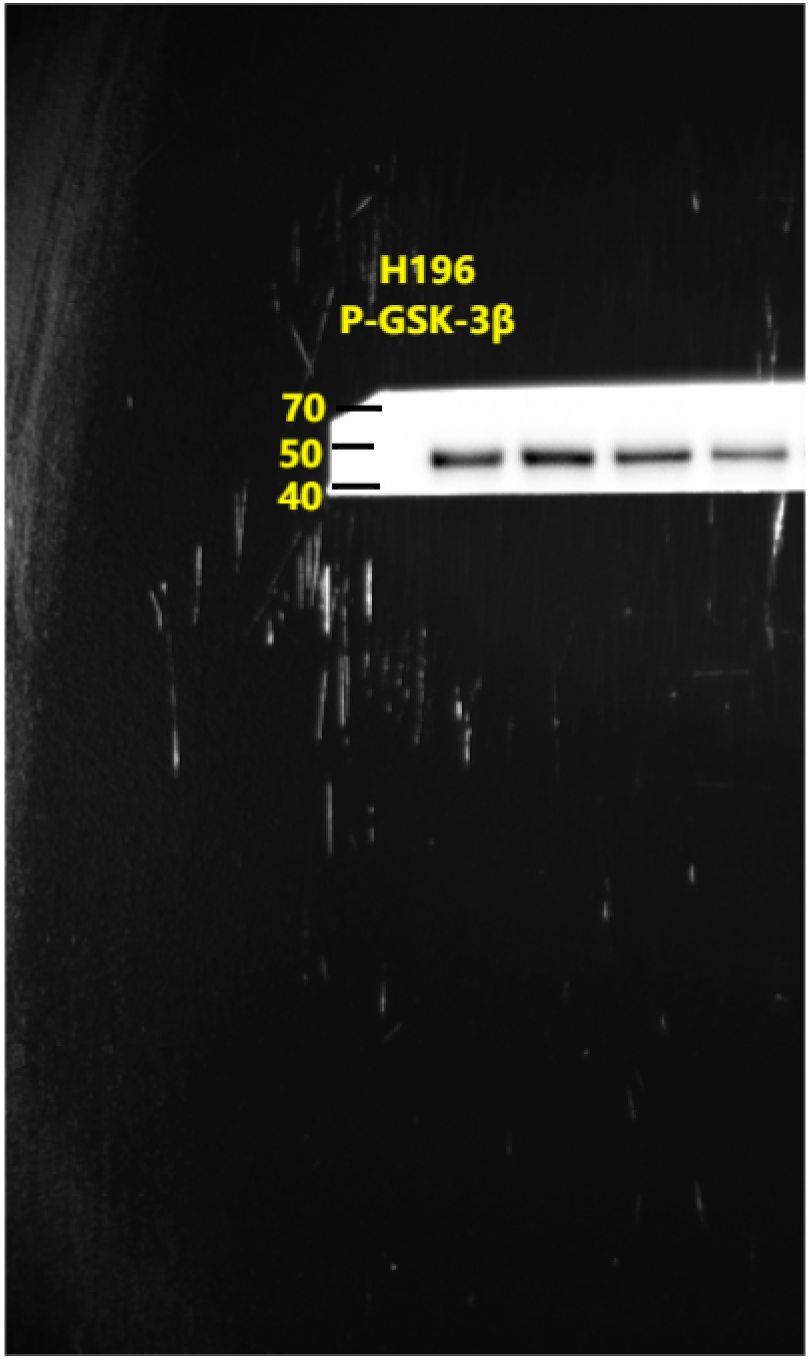
H196
P-GSK-3β
70
50
40

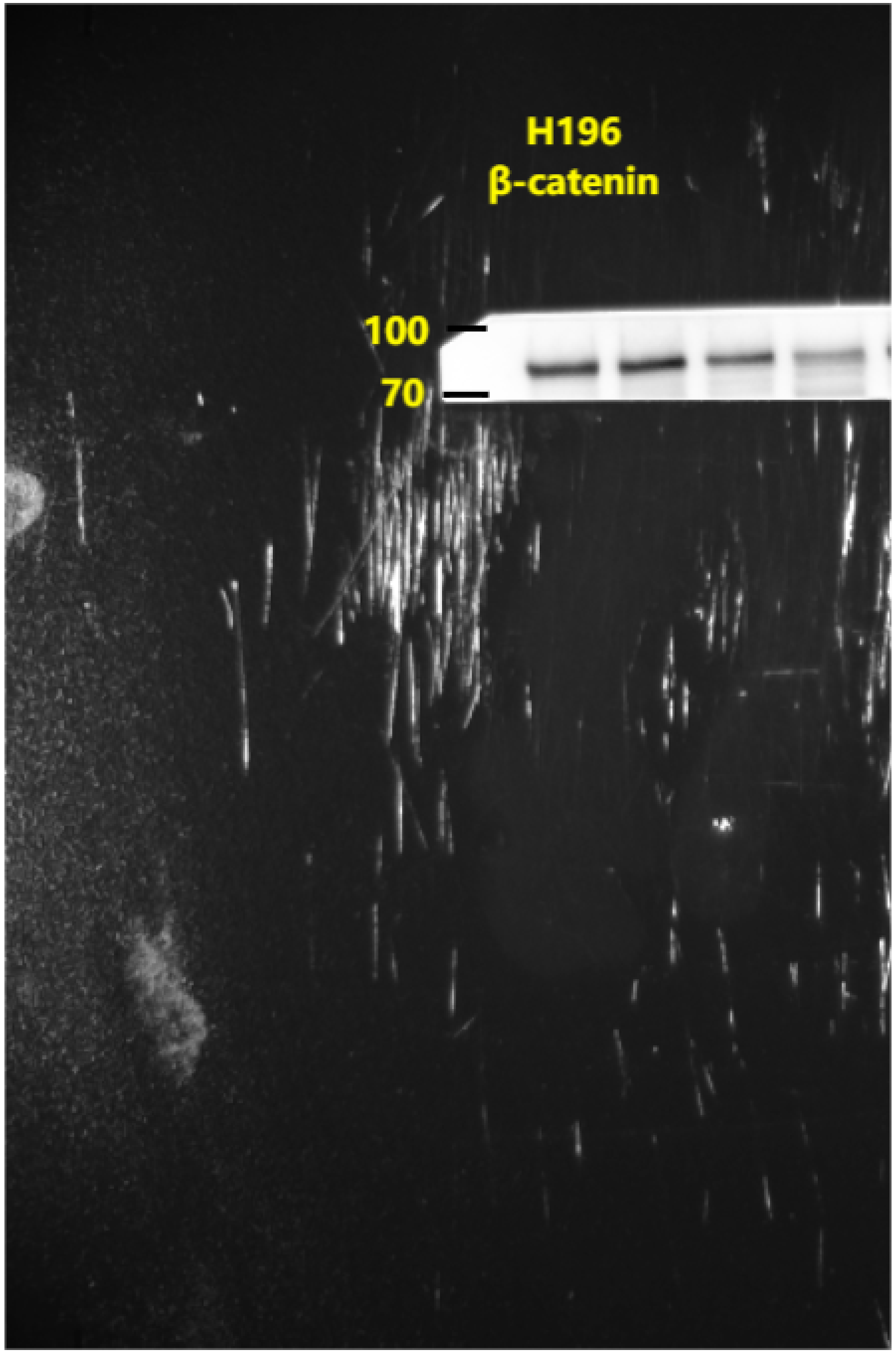
H196
β-catenin
100
70

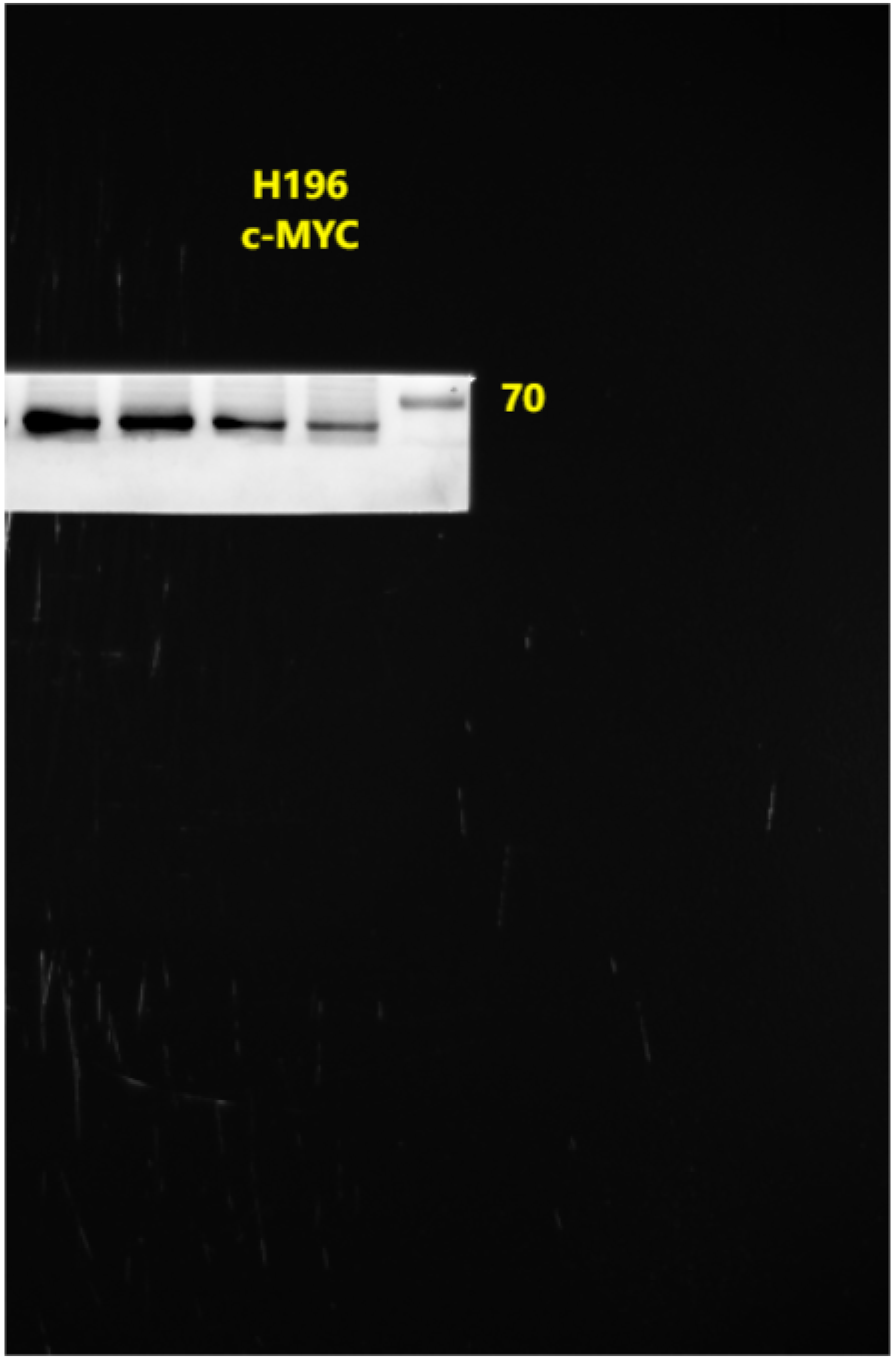
H196
c-MYC
70

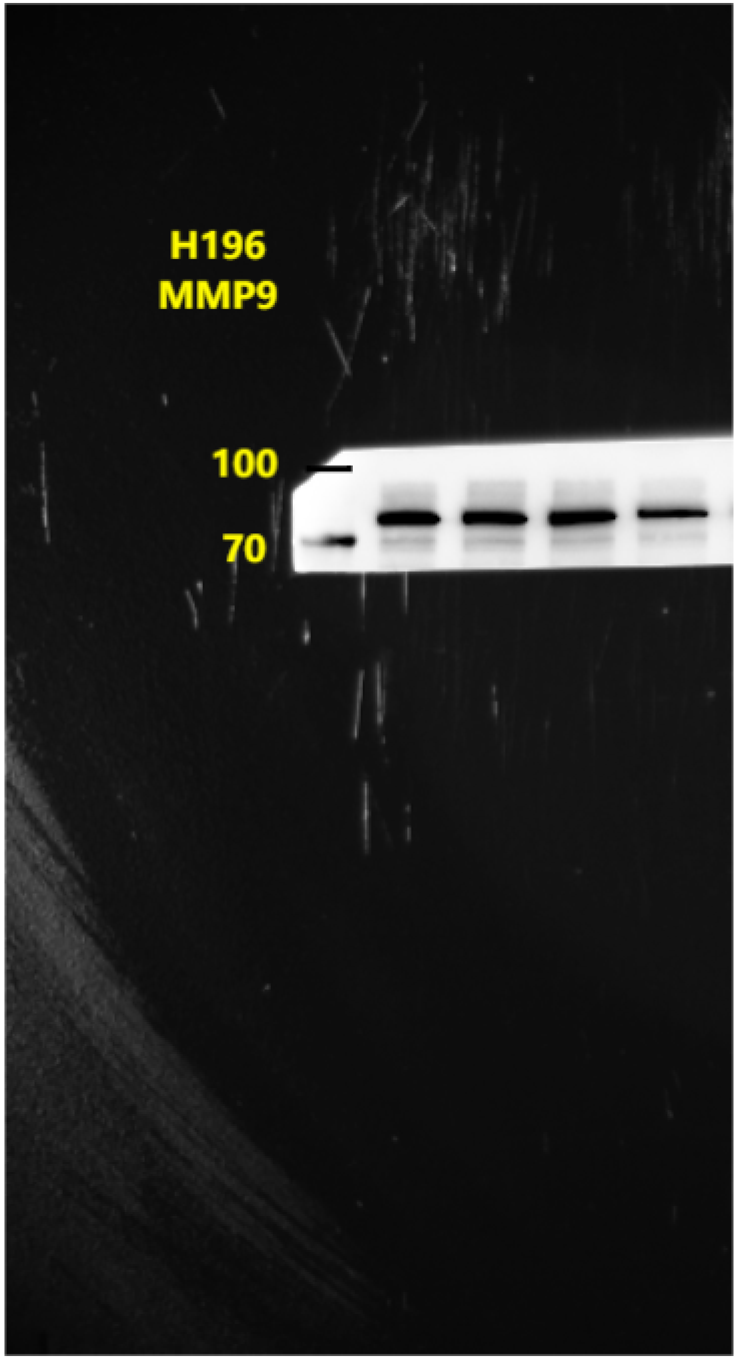
H196
MMP9
100
70

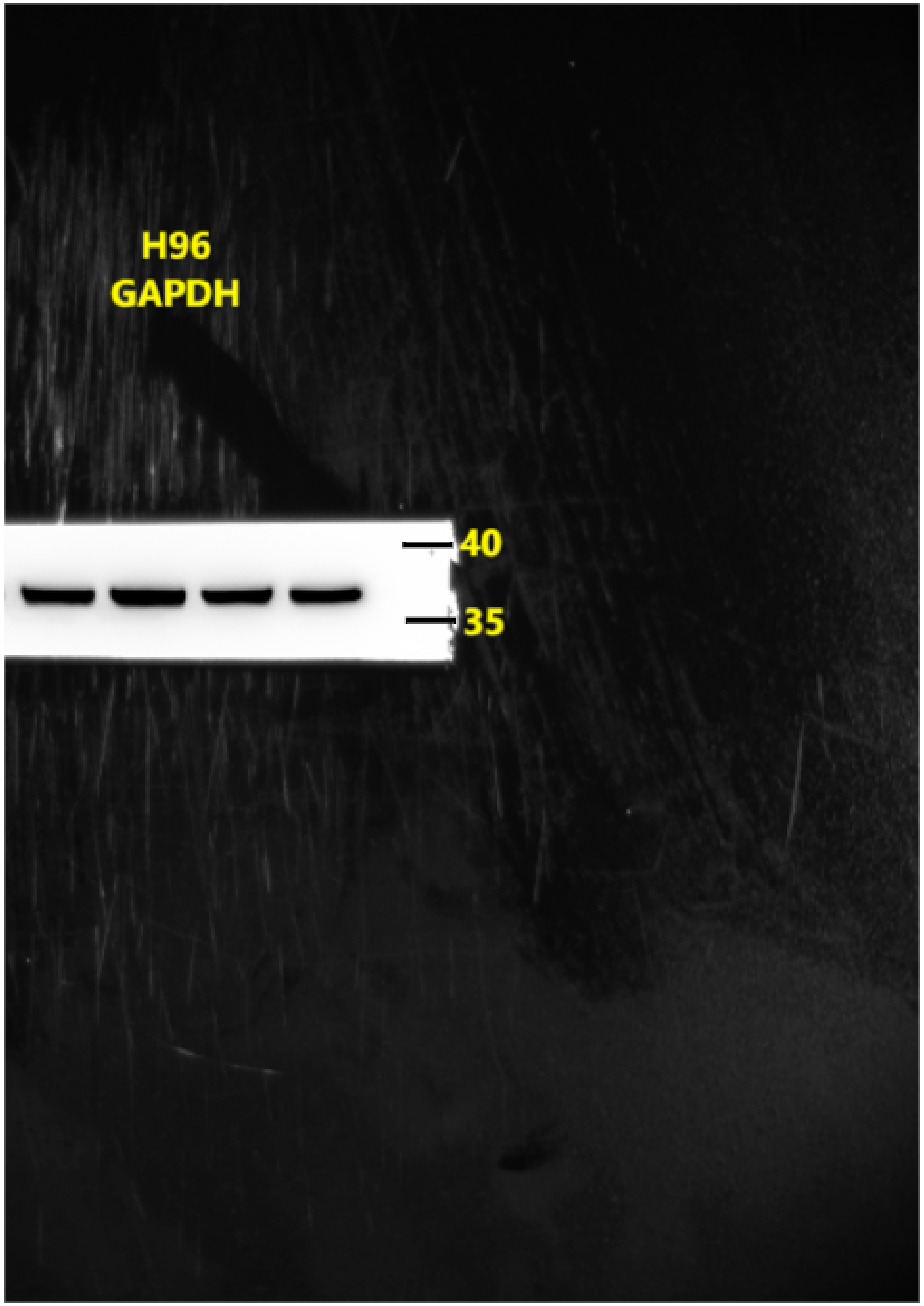
H96
GAPDH
40
35

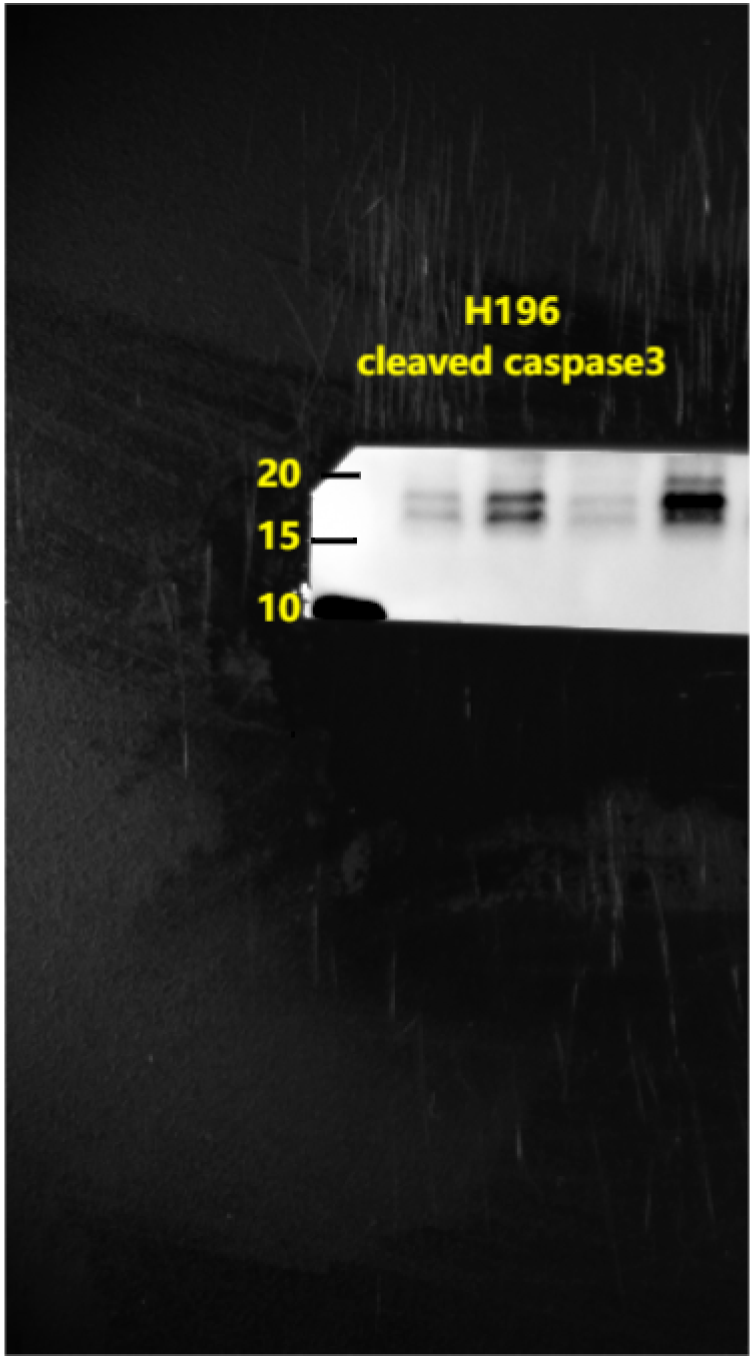
H196
cleaved caspase3
20
15
10

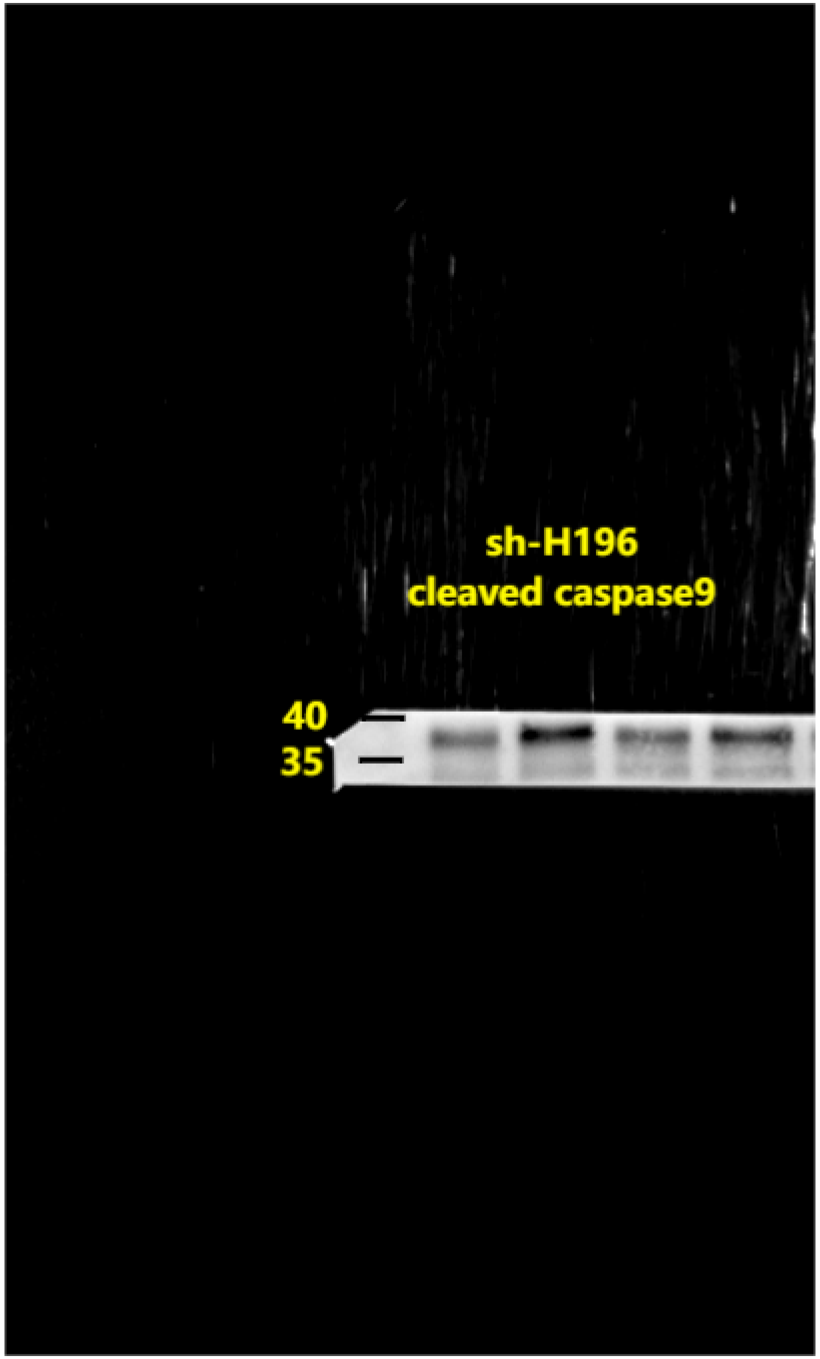
sh-H196
cleaved caspase9
40
35

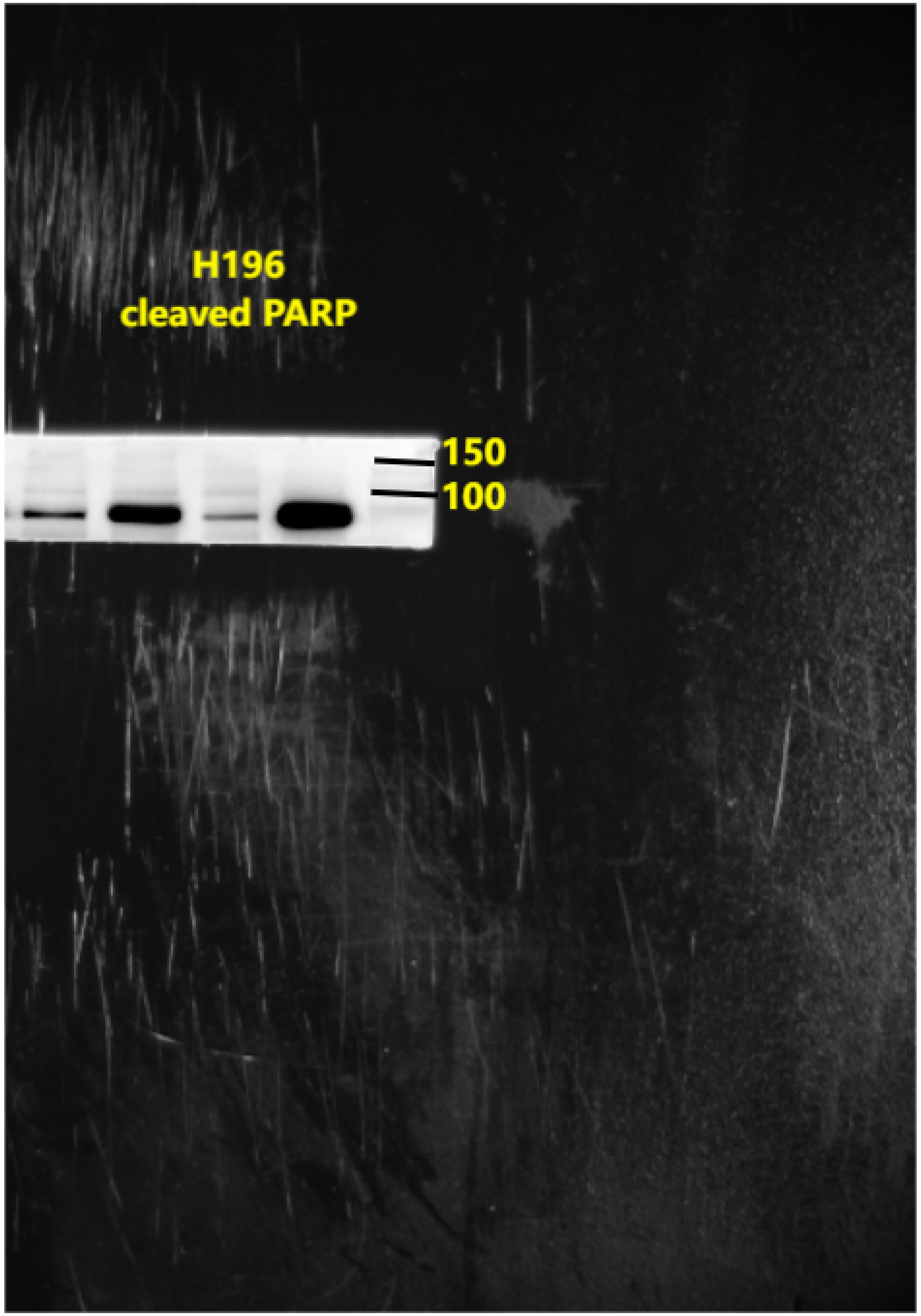
H196
cleaved PARP
150
100

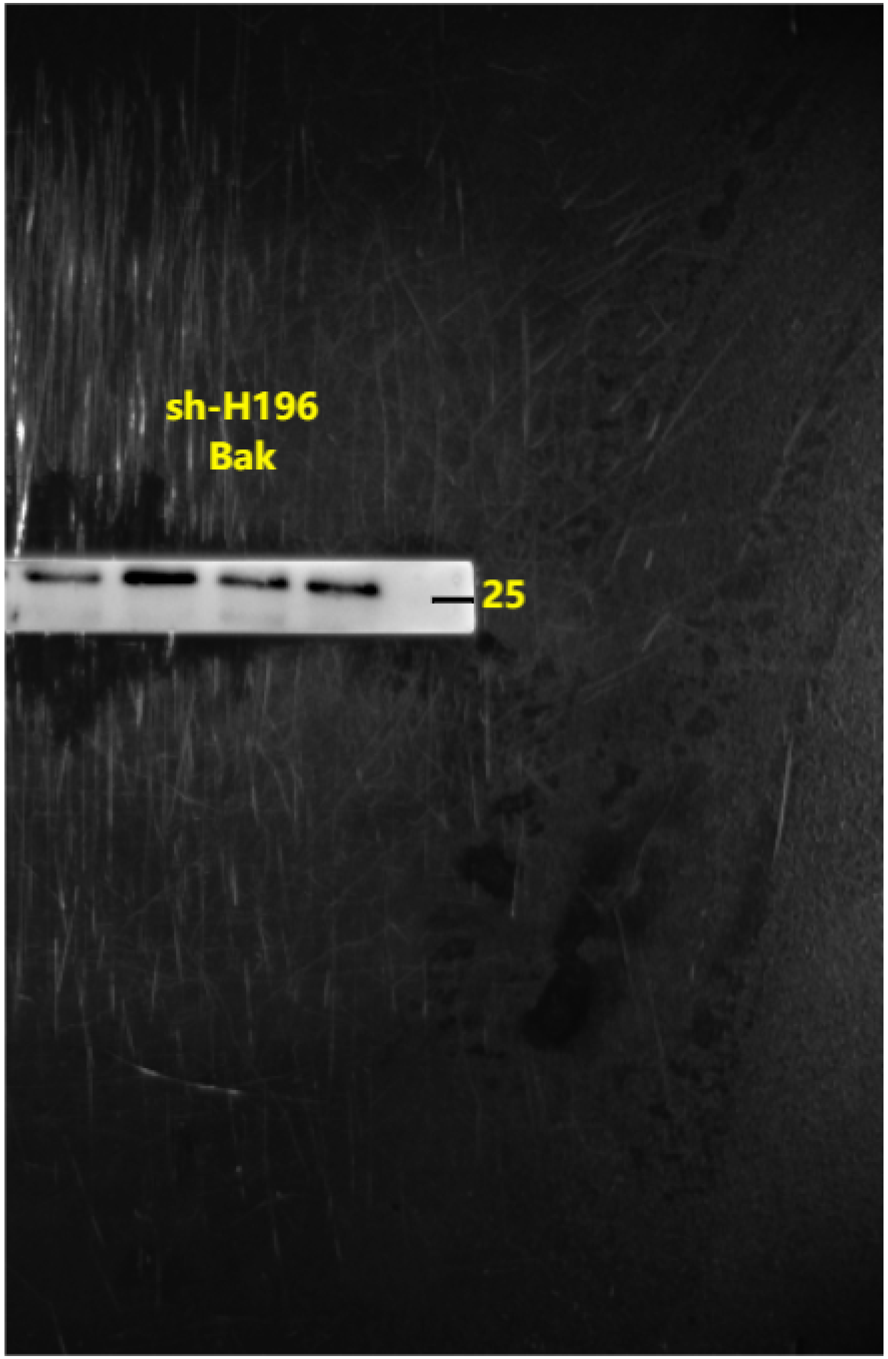
sh-H196
Bak
25

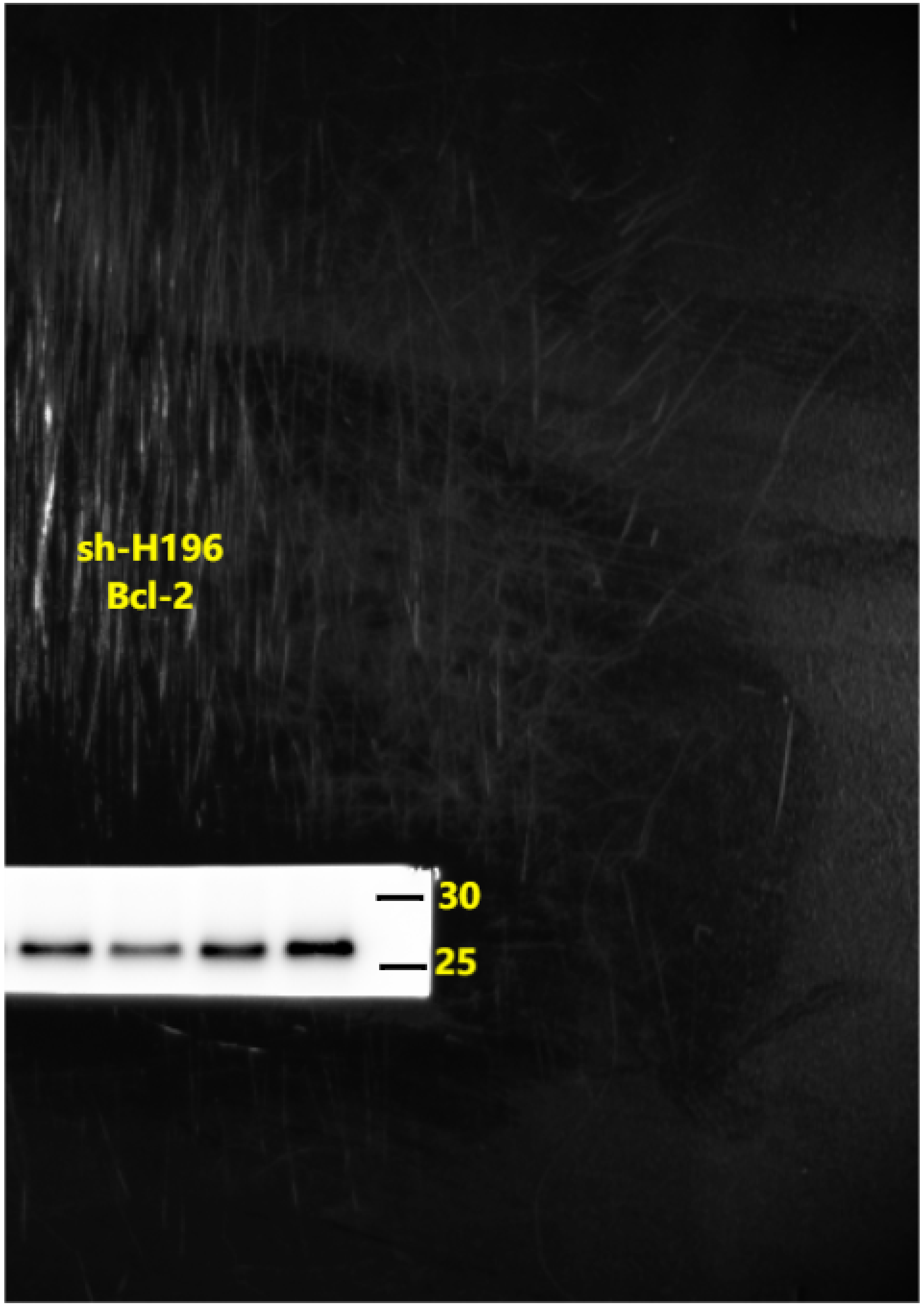
sh-H196
Bcl-2
30
25

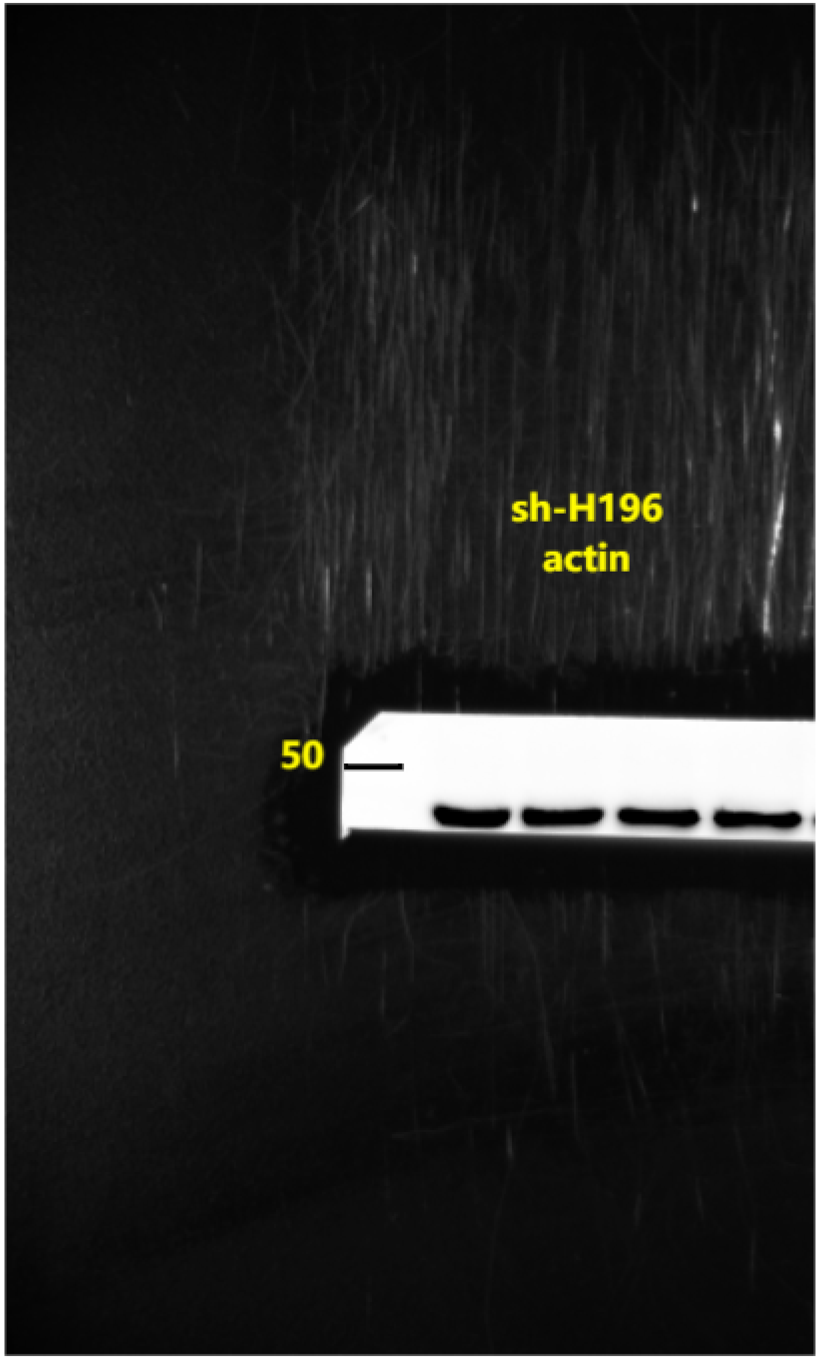
sh-H196
actin
50

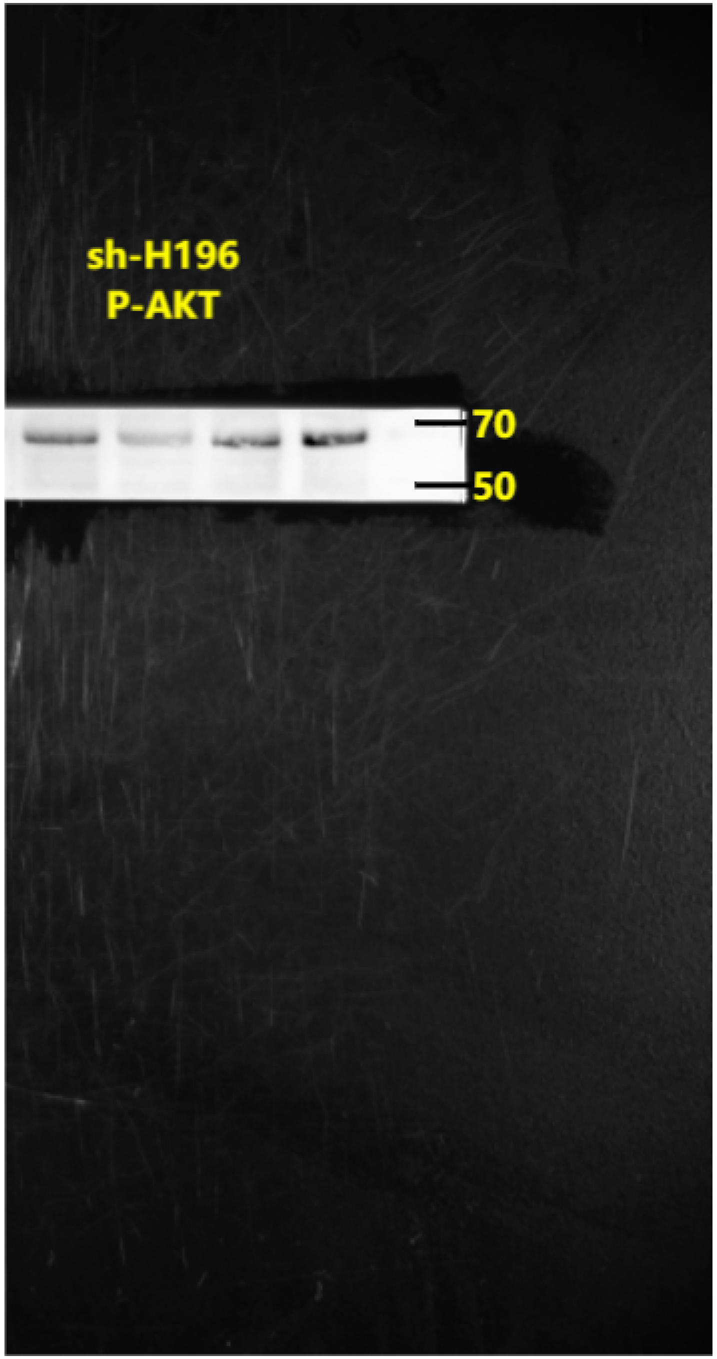
sh-H196
P-AKT
70
50

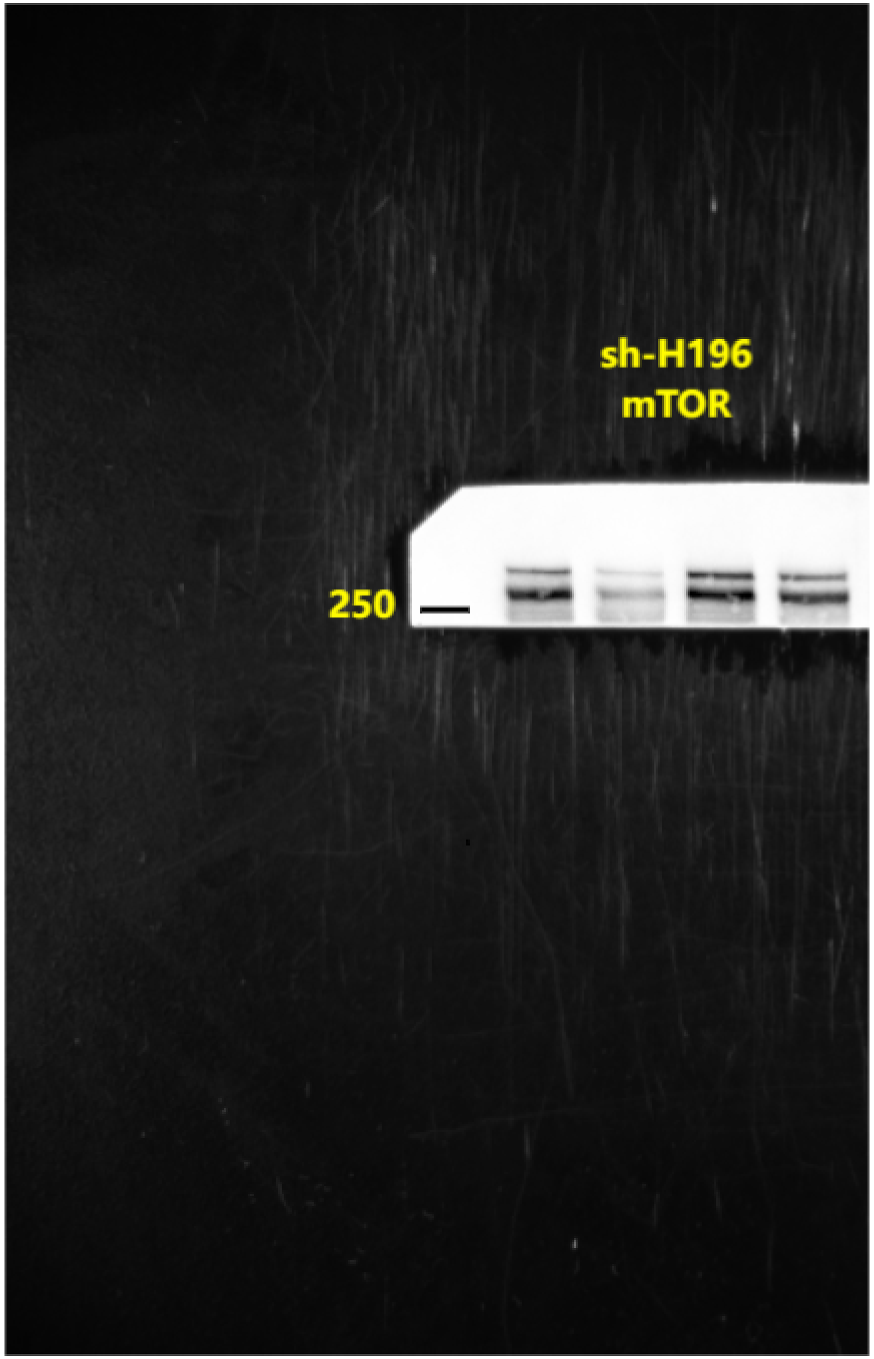
sh-H196
mTOR
250

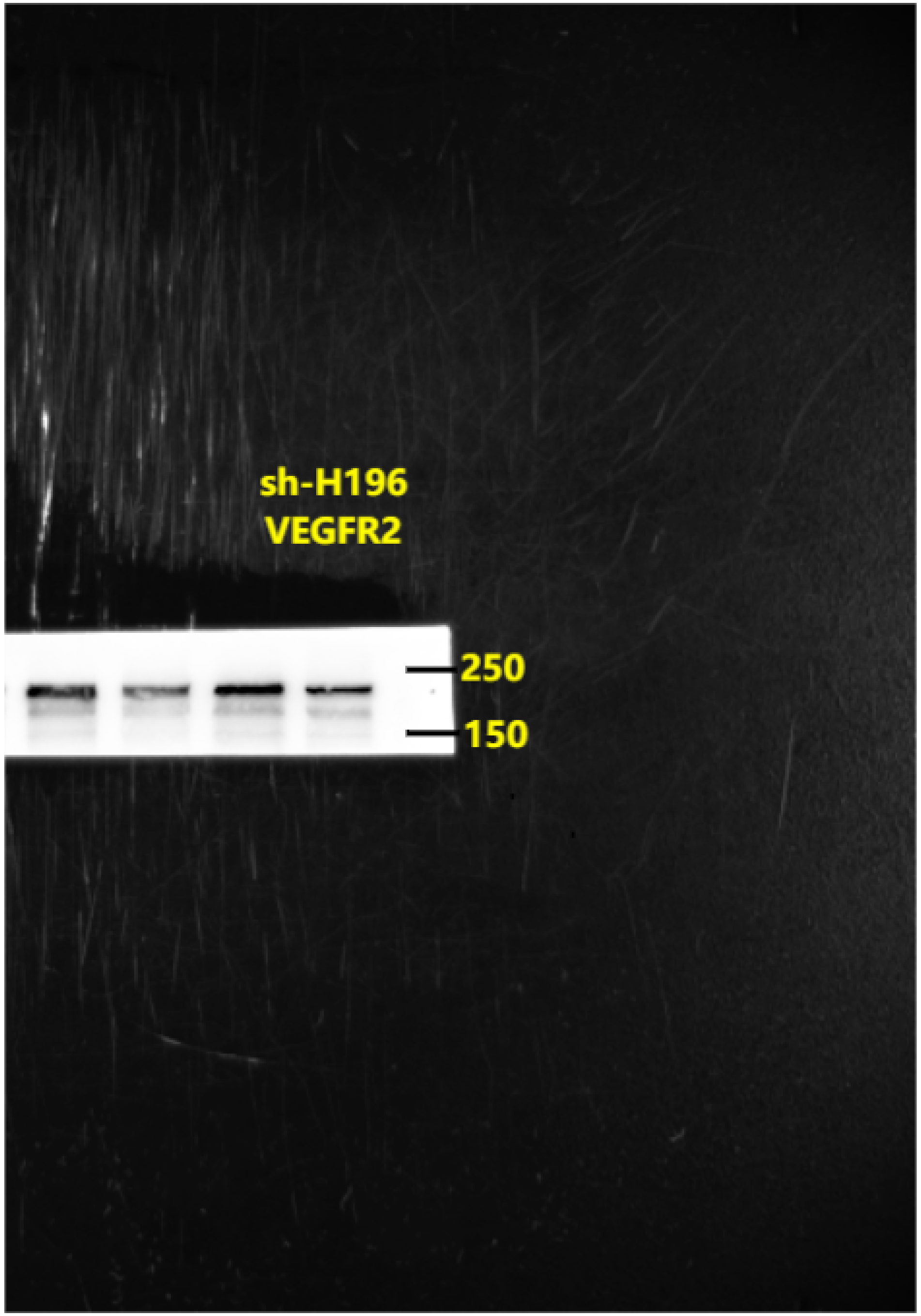
sh-H196
VEGFR2
250
150

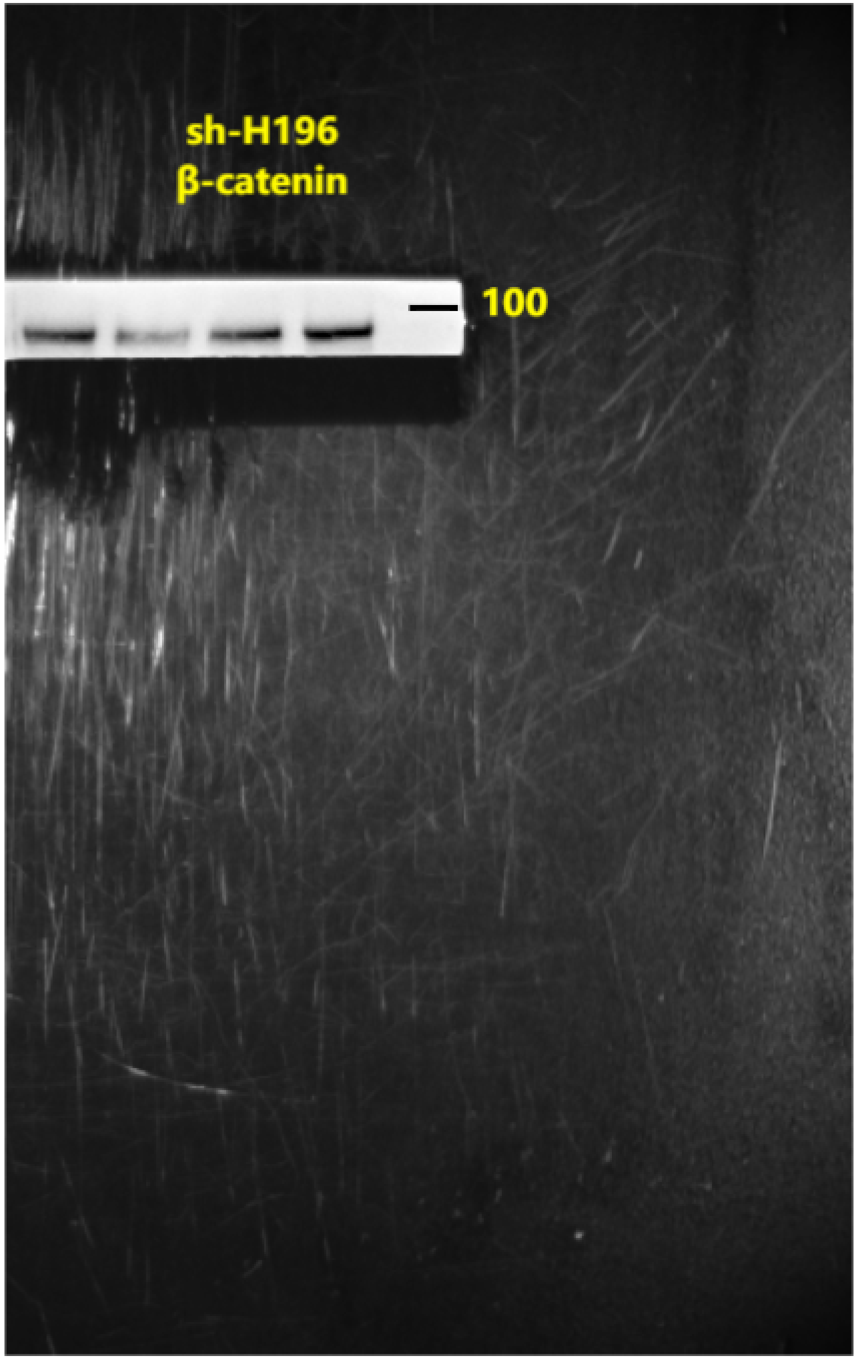
sh-H196
β-catenin
100

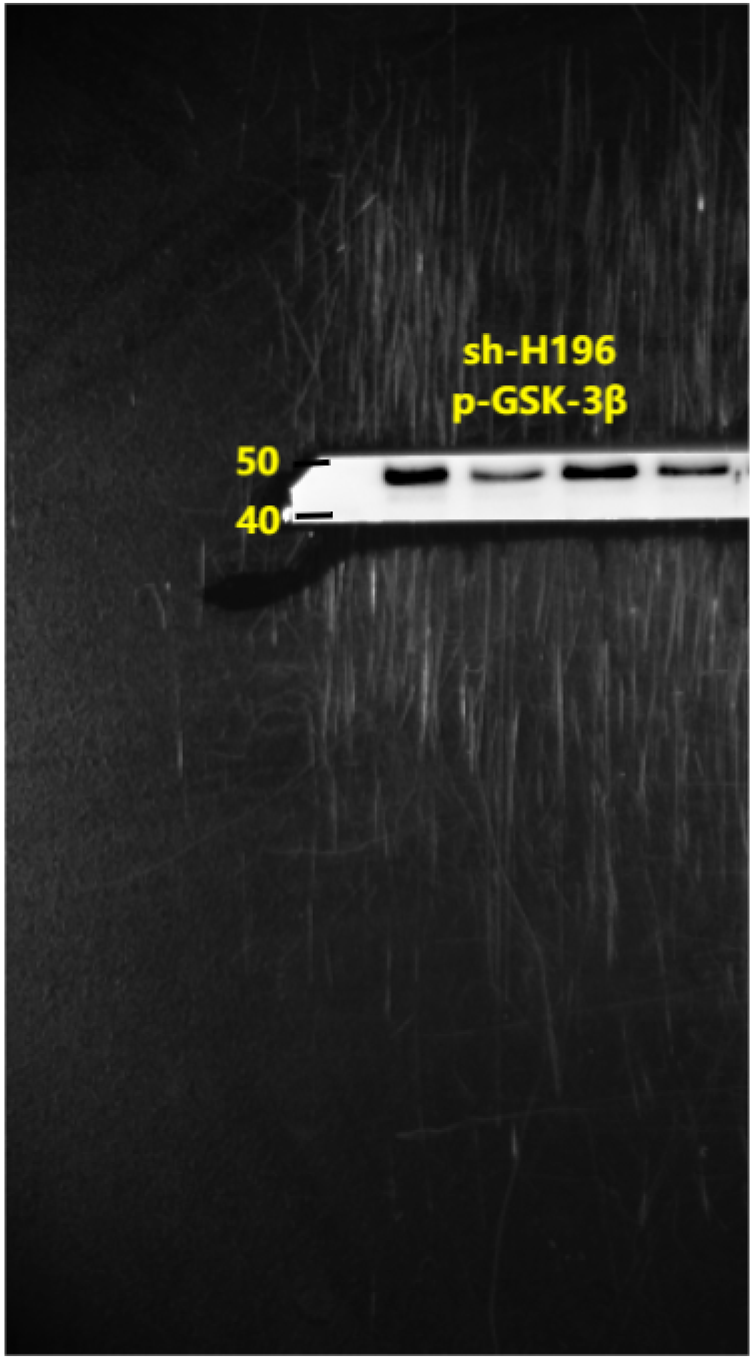
sh-H196
p-GSK-3β
50
40

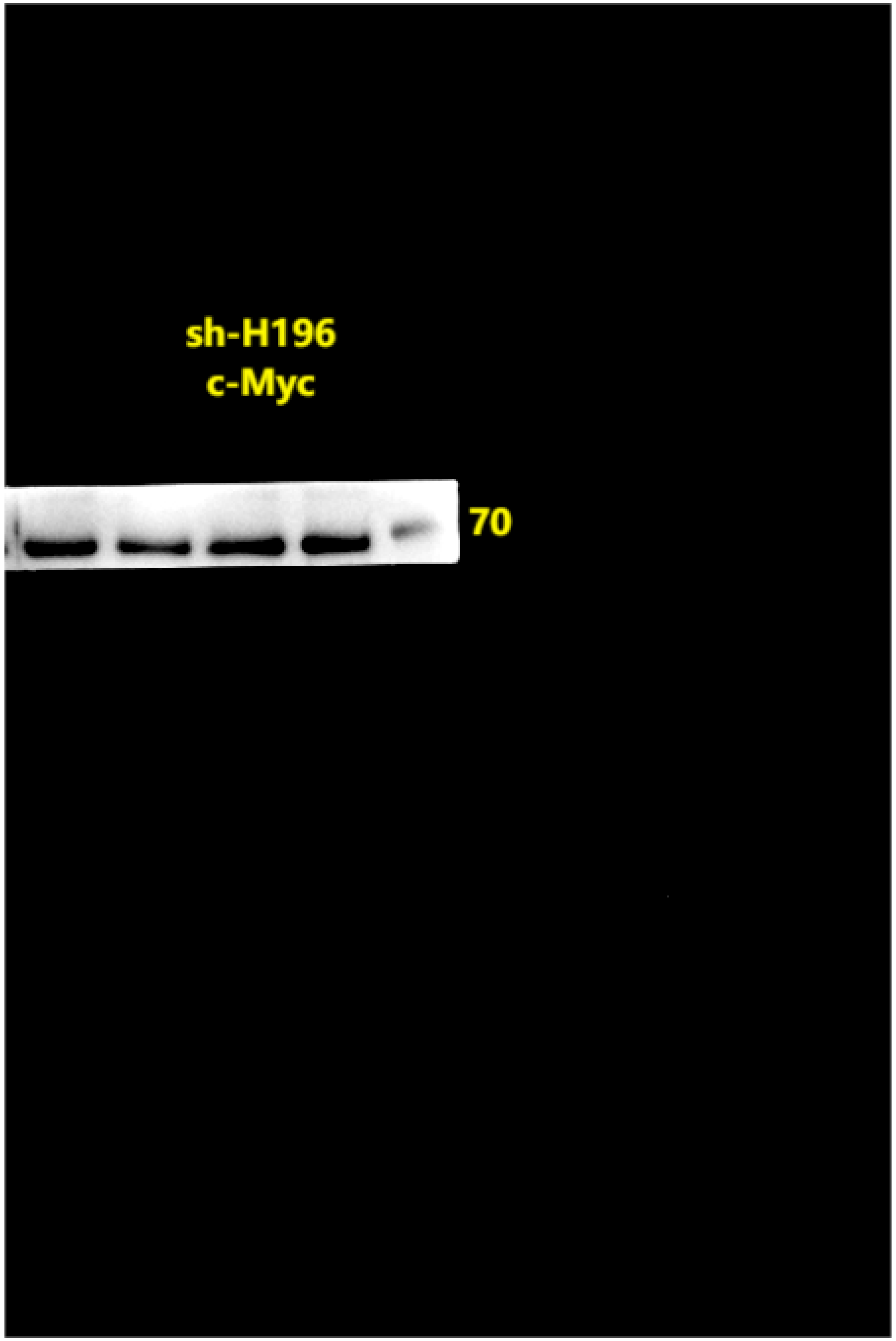
sh-H196
c-Myc
70

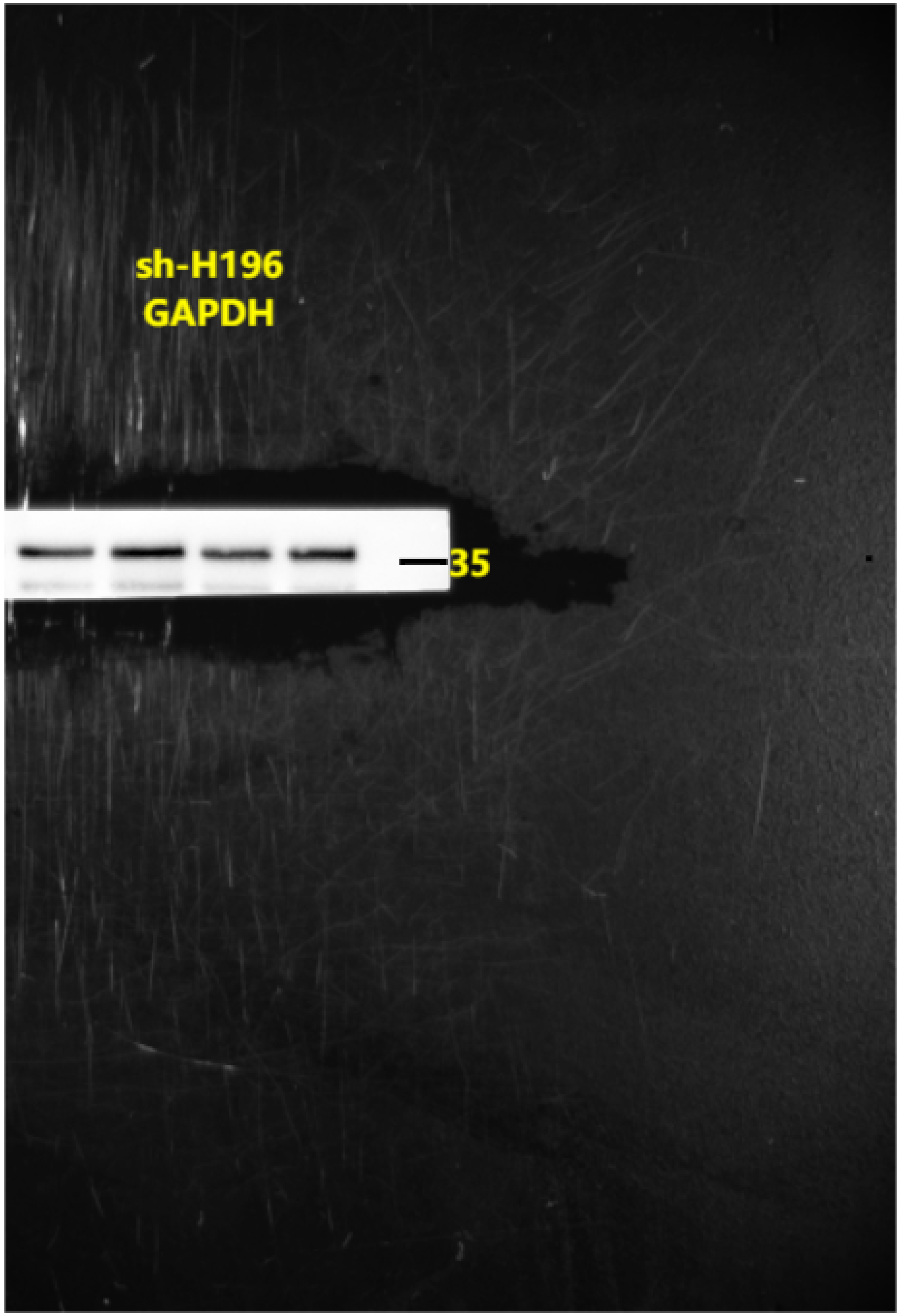
sh-H196
GAPDH
35
