# Supplementary material for: Combination of arsenic trioxide and apatinib synergistically inhibits small cell lung cancer by down-regulating VEGFR2/mTOR and Akt/c-Myc signaling pathway via GRB10
Source: Hereditas. 2024 Sep 2;161:29. doi: 10.1186/s41065-024-00330-2 (PMC11367874; doi:10.1186/s41065-024-00330-2)
Supplement: Supplementary file 2 — Supplementary Material 2 [file 41065_2024_330_MOESM2_ESM.pdf]

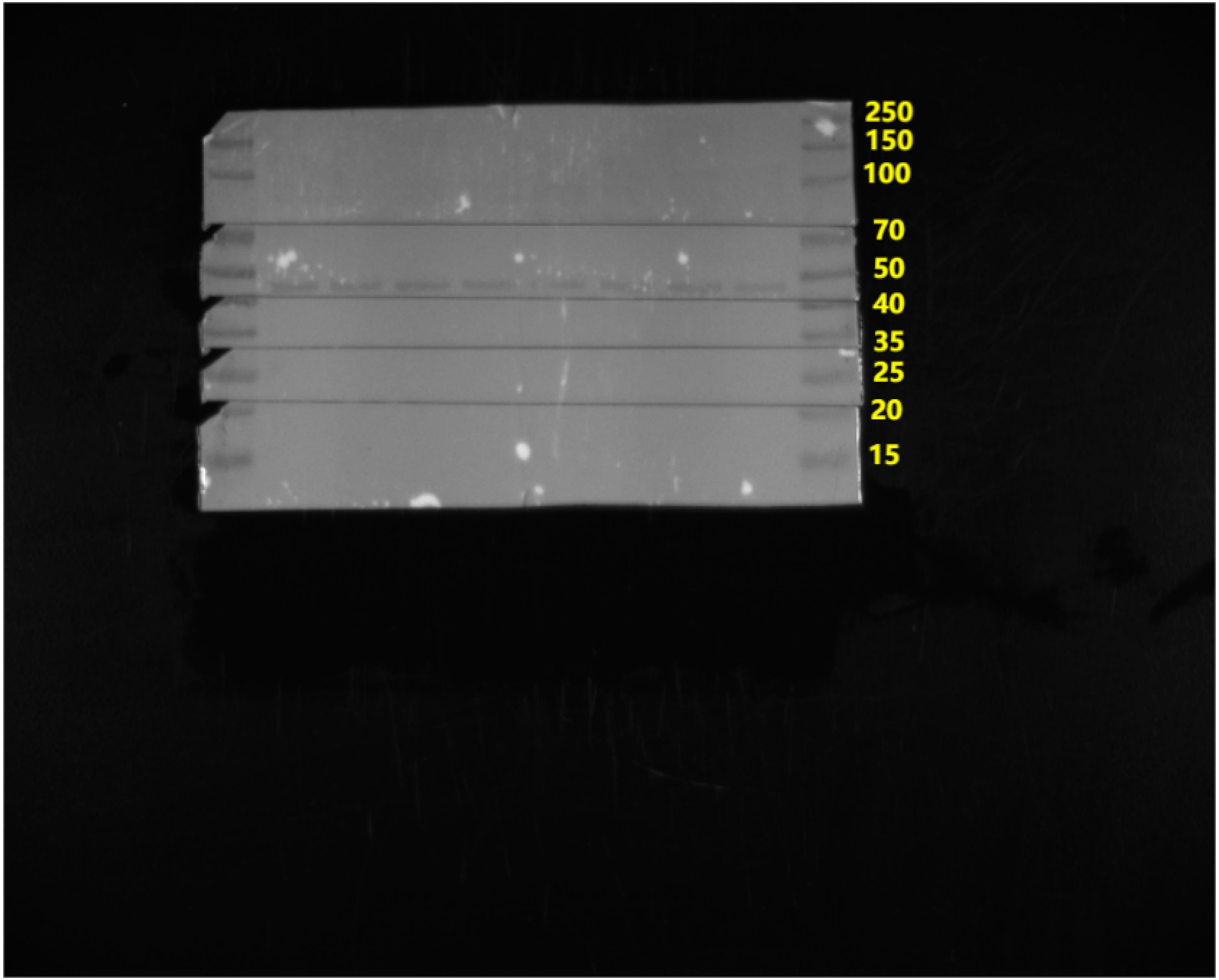
250
150
100
70
50
40
35
25
20
15

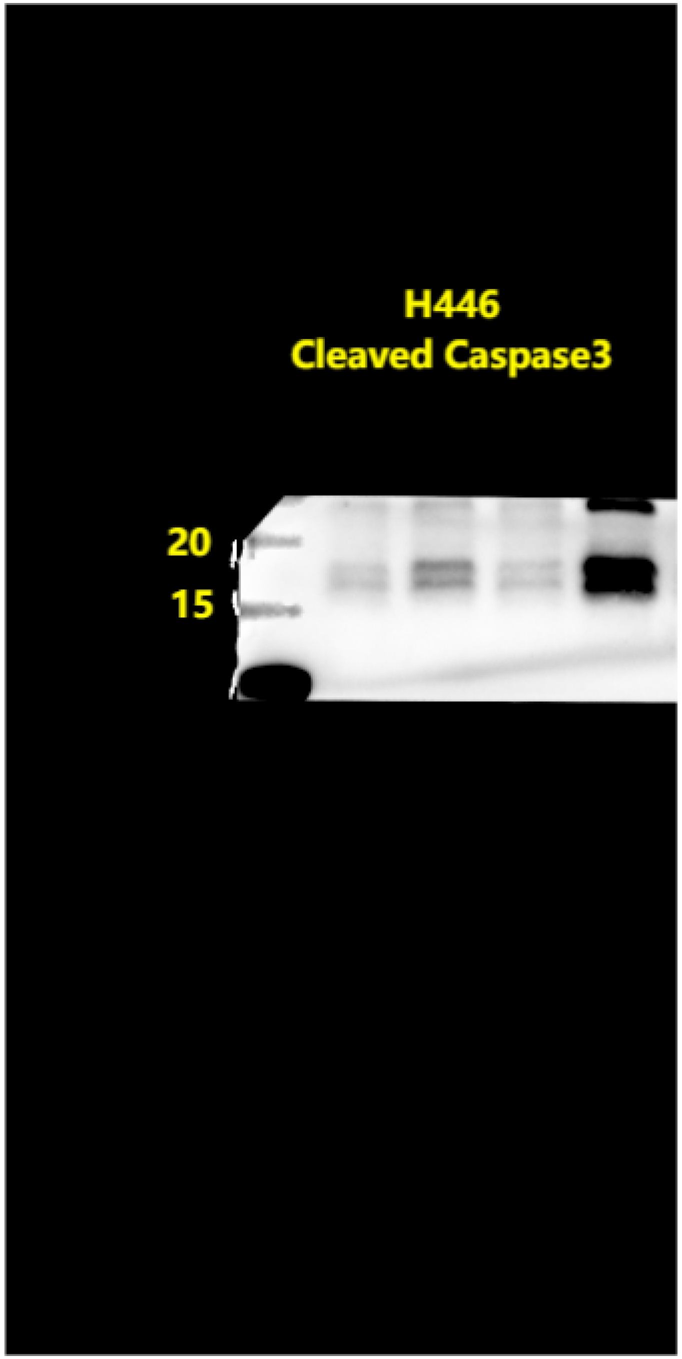
H446
Cleaved Caspase3
20
15

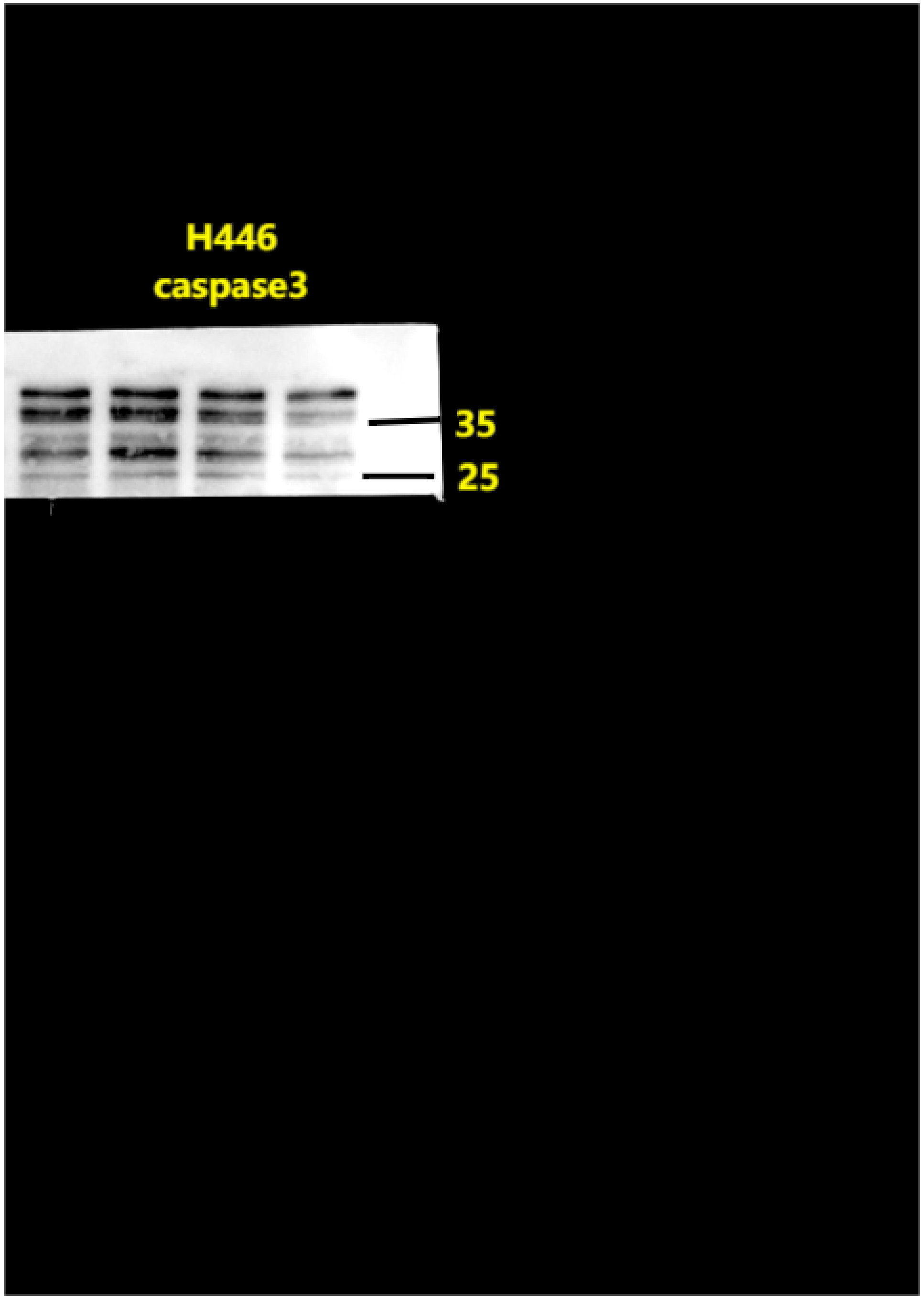
H446
caspase3
35
25

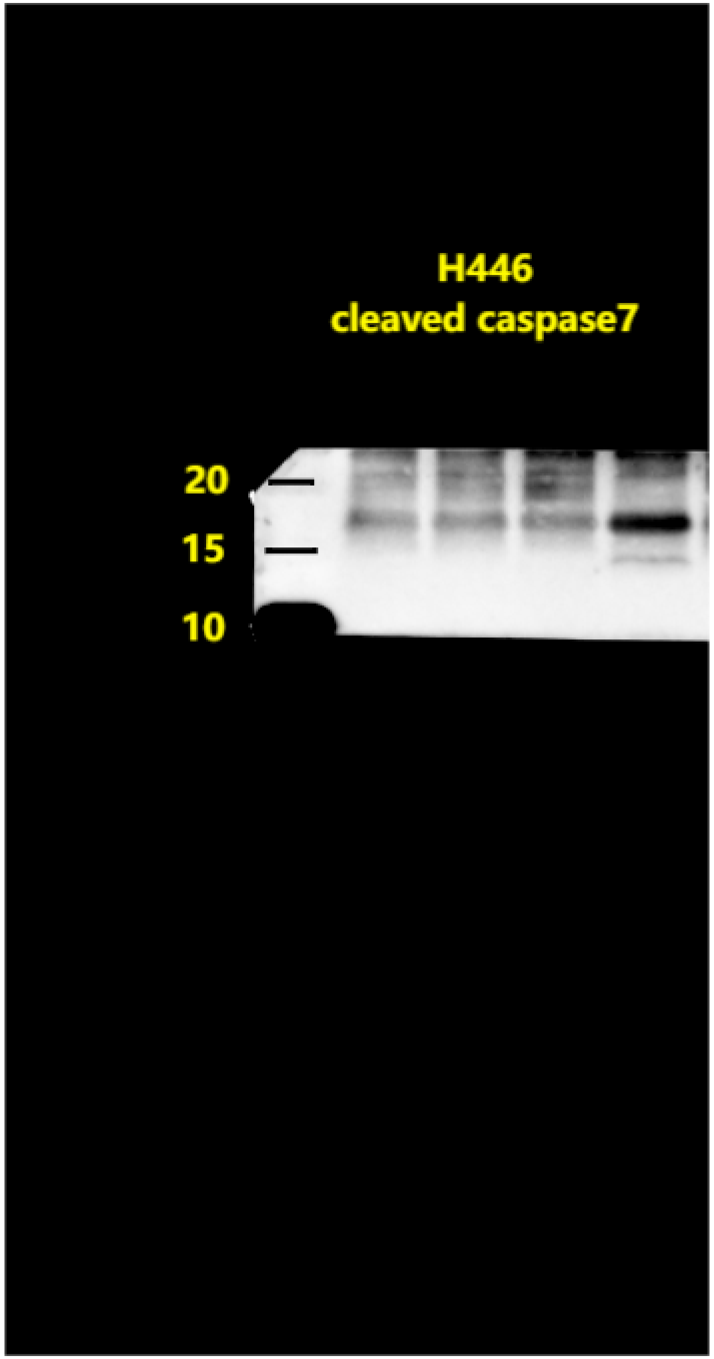

H446
cleaved caspase7
20
15
10

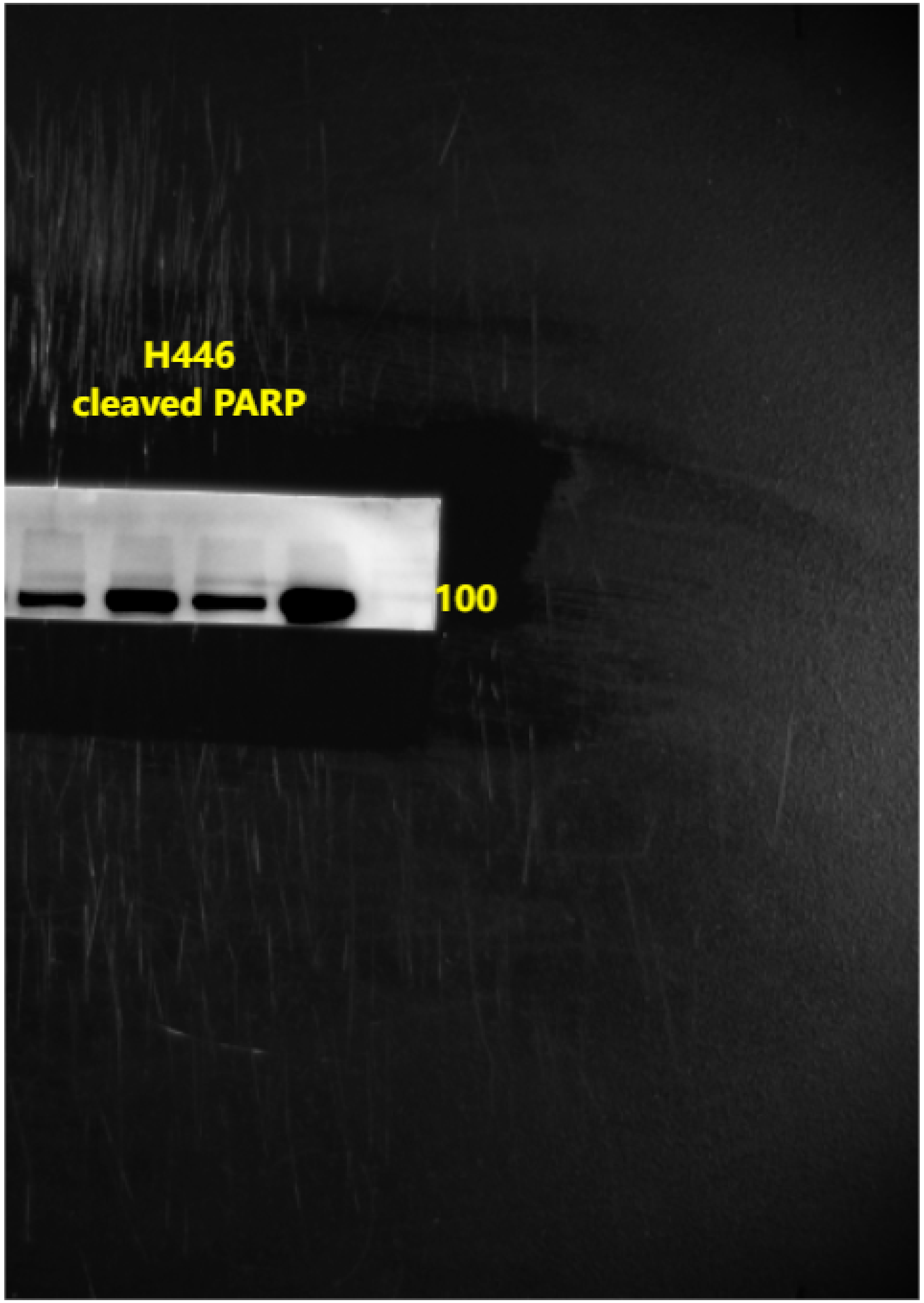
H446
cleaved PARP
100

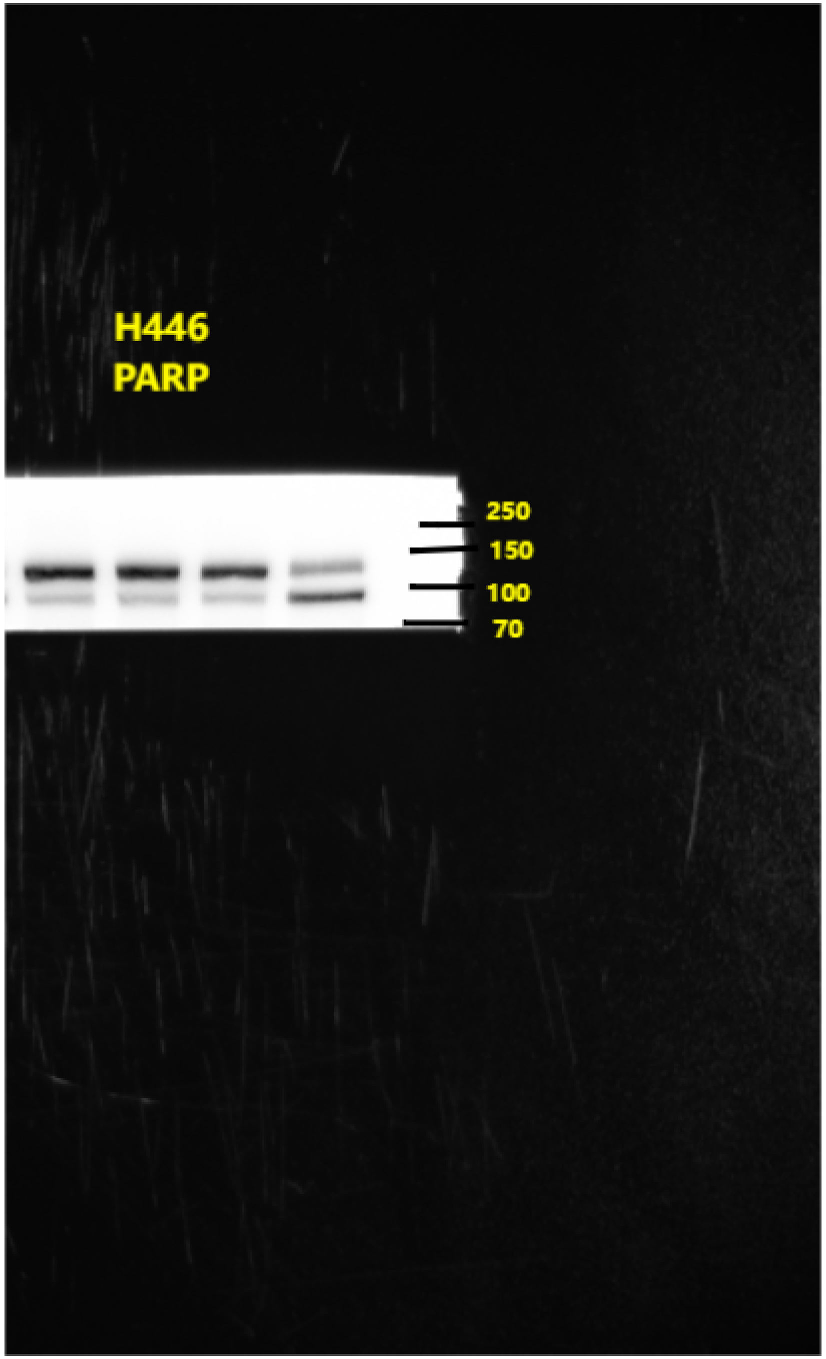
H446
PARP
250
150
100
70

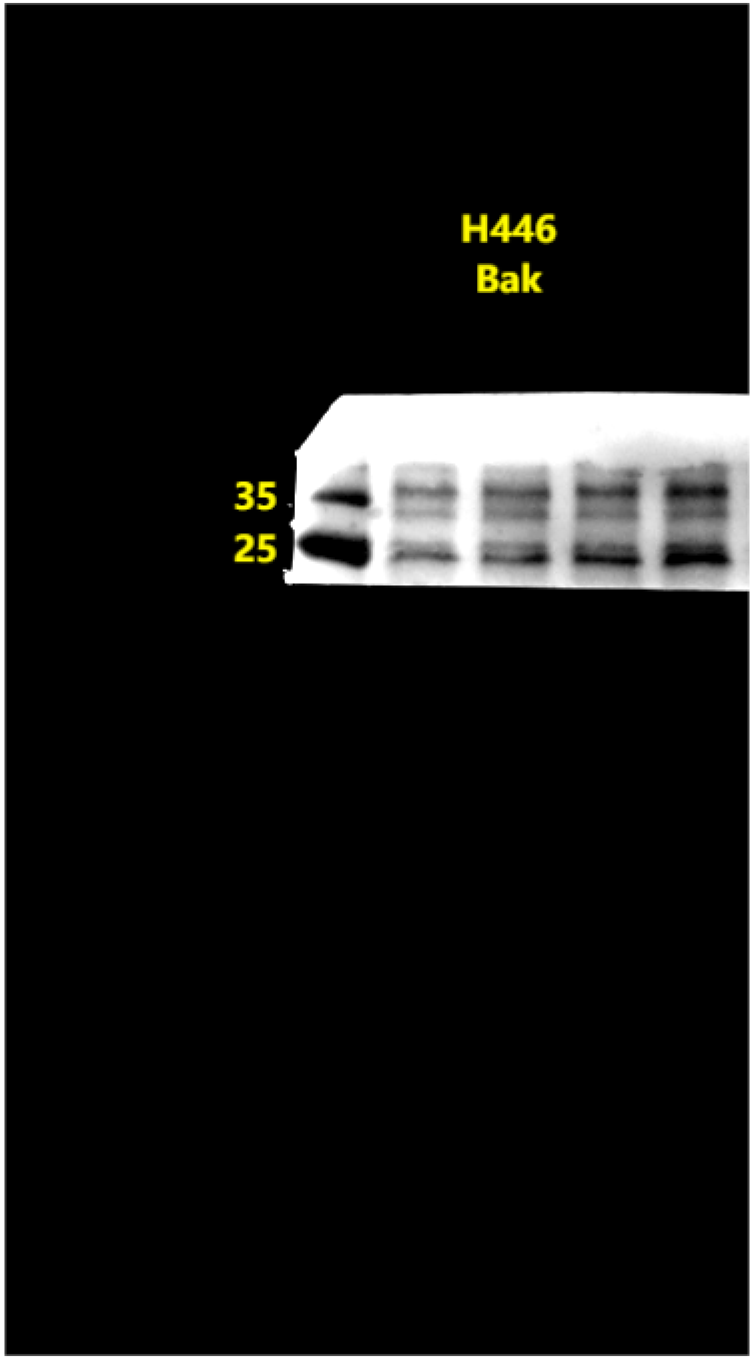
H446
Bak
35
25

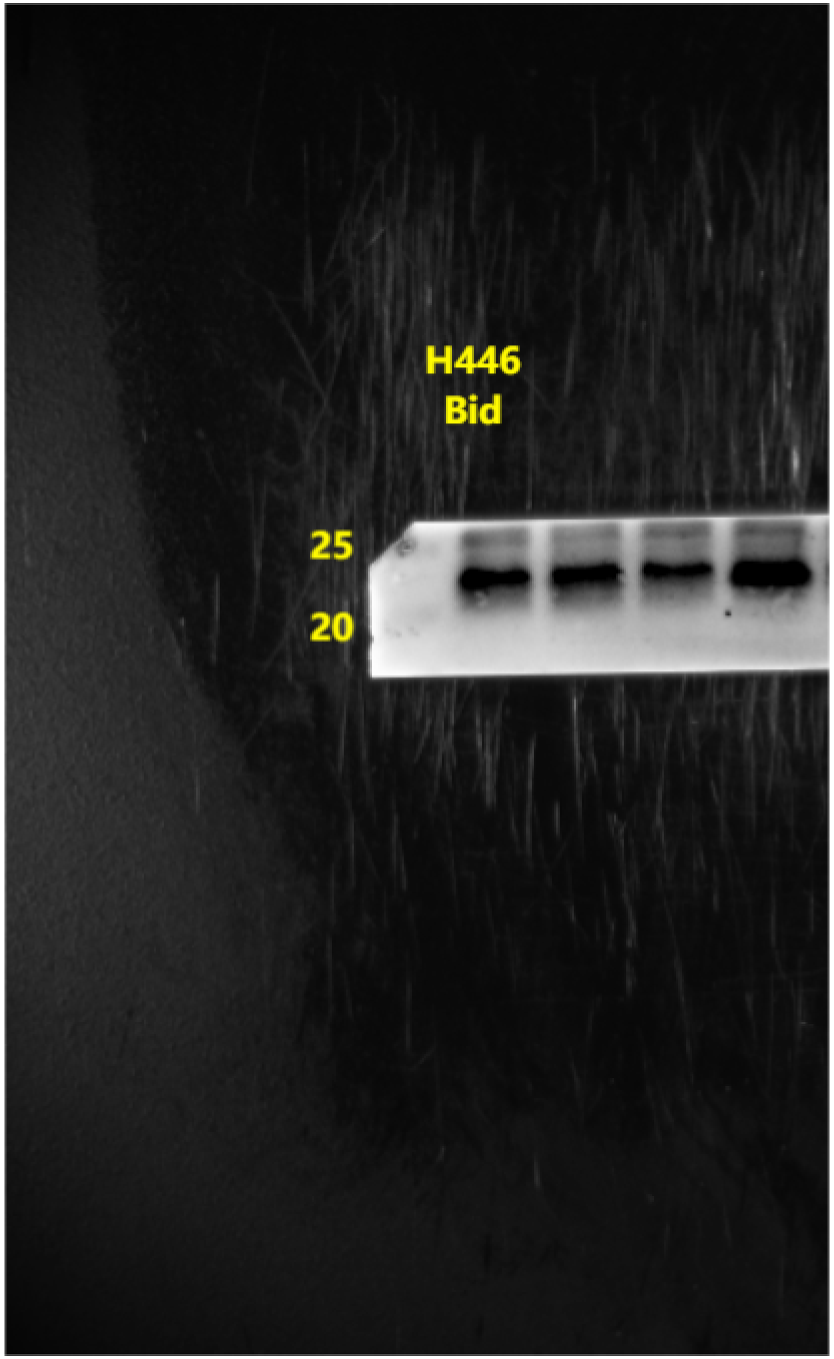
H446
Bid
25
20

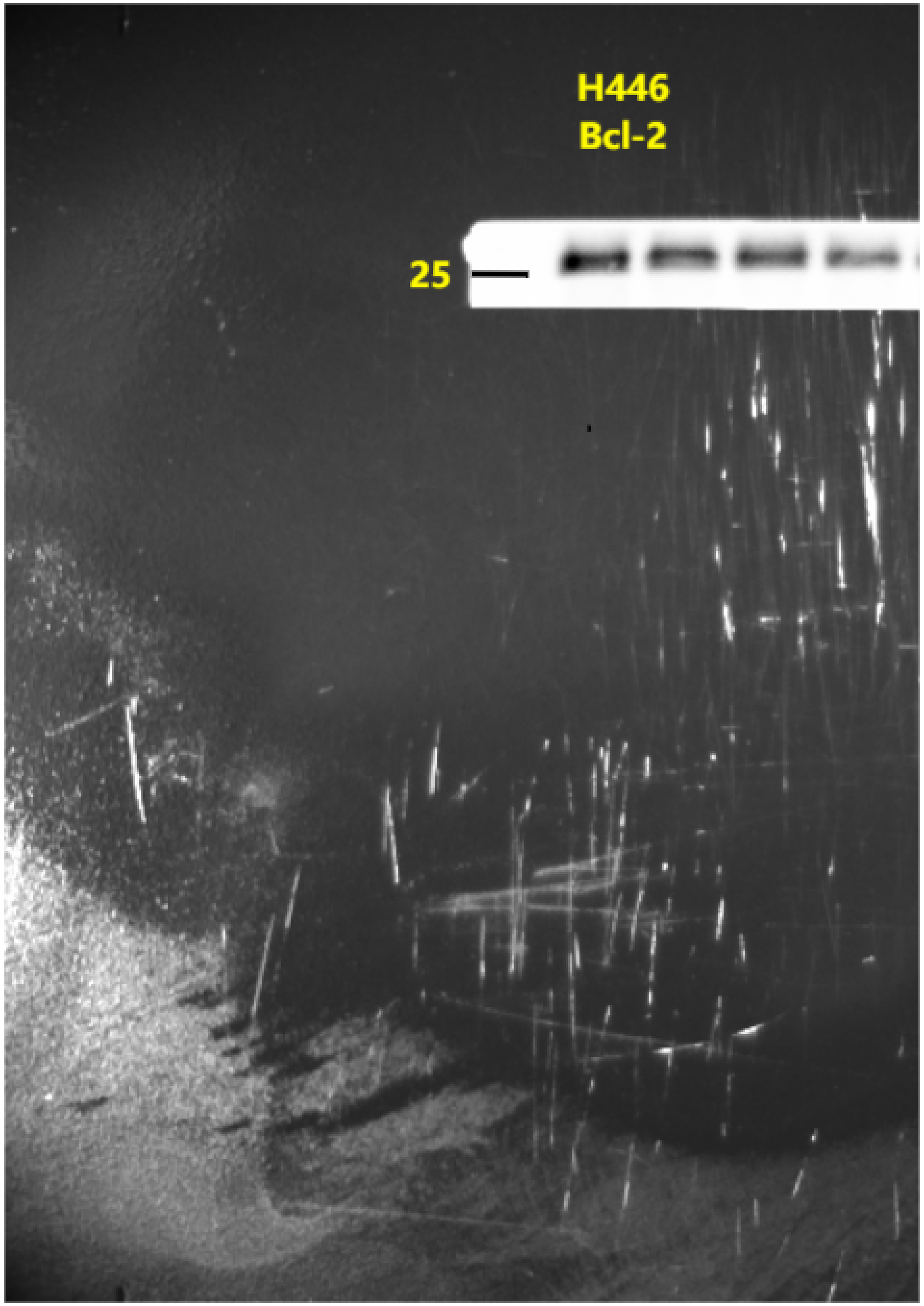
H446
Bcl-2
25

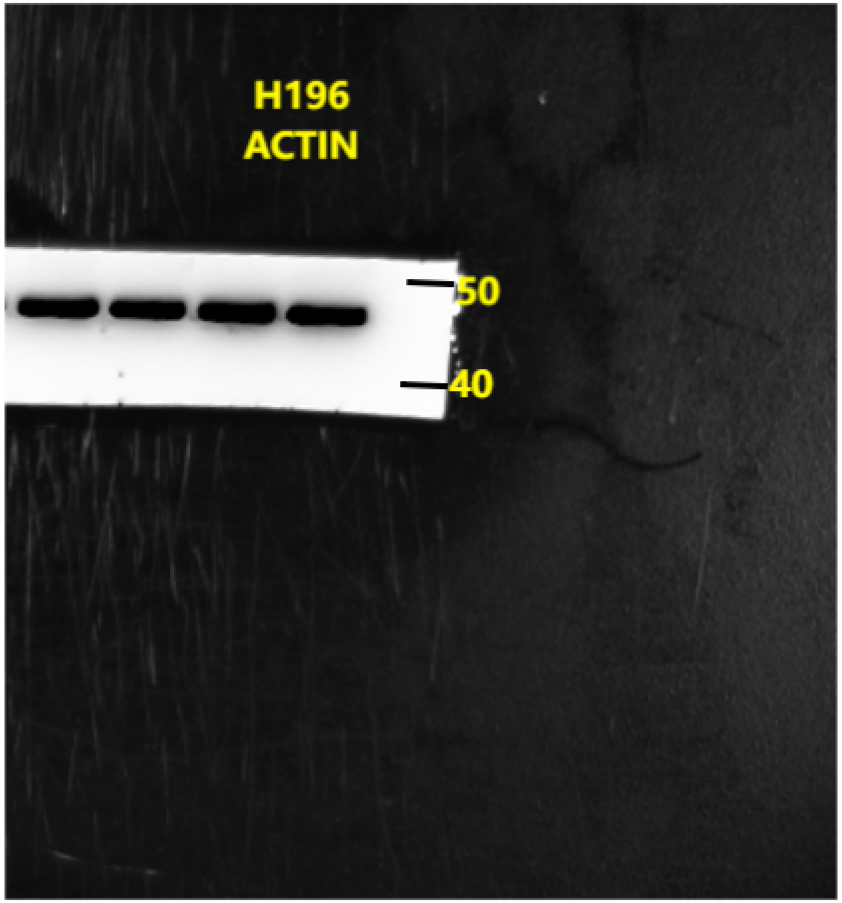
H196
ACTIN
50
40

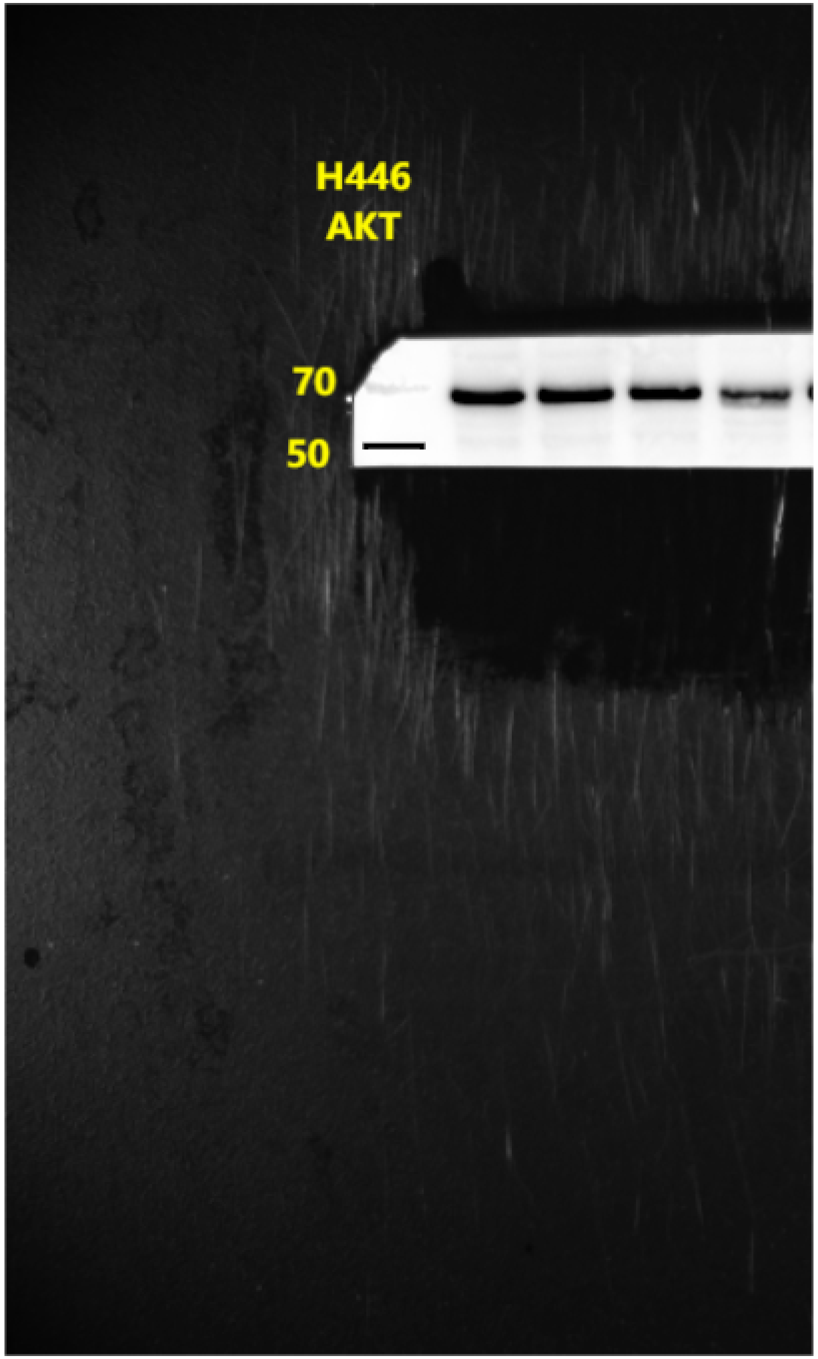
H446
AKT
70
50

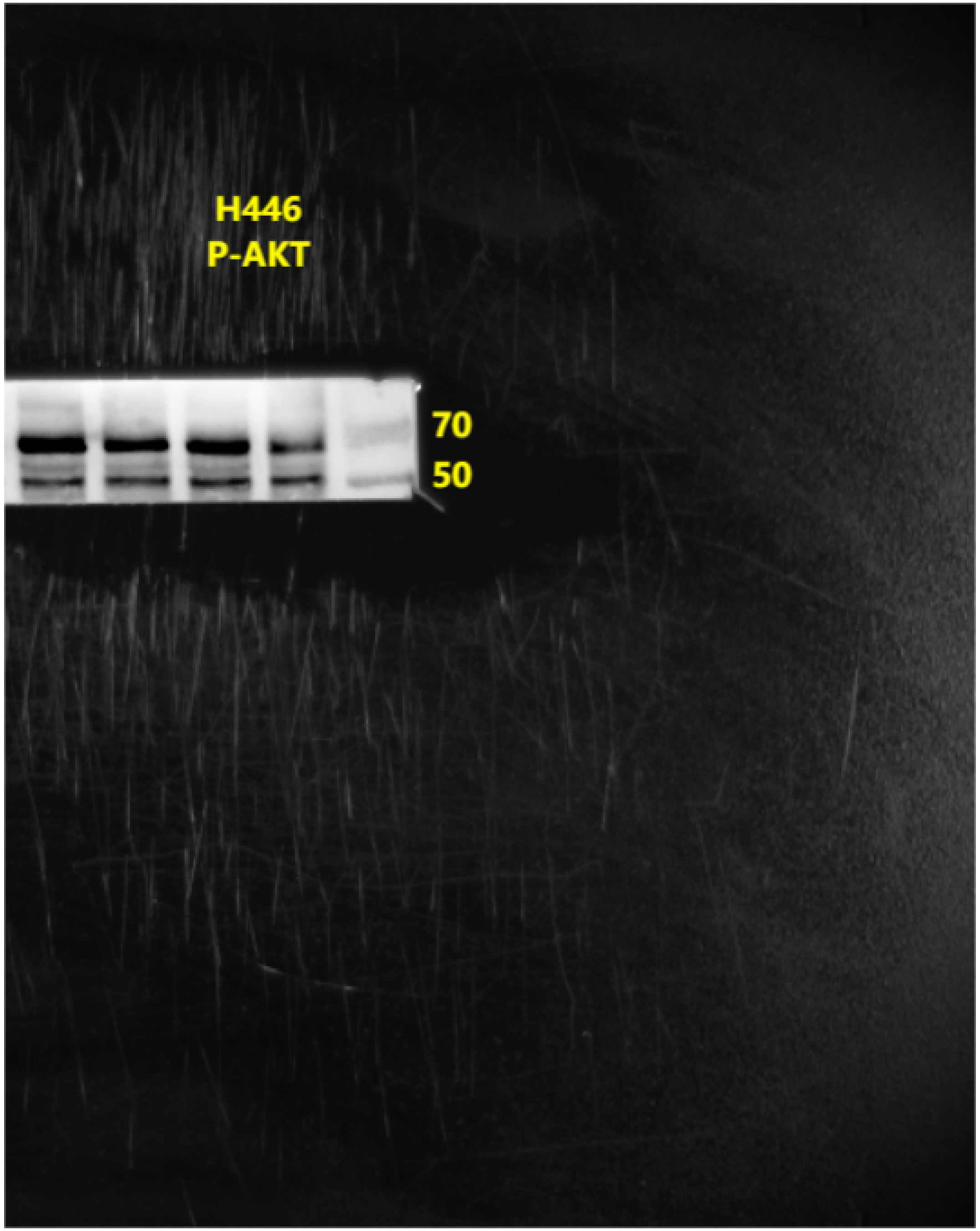
H446
P-AKT
70
50

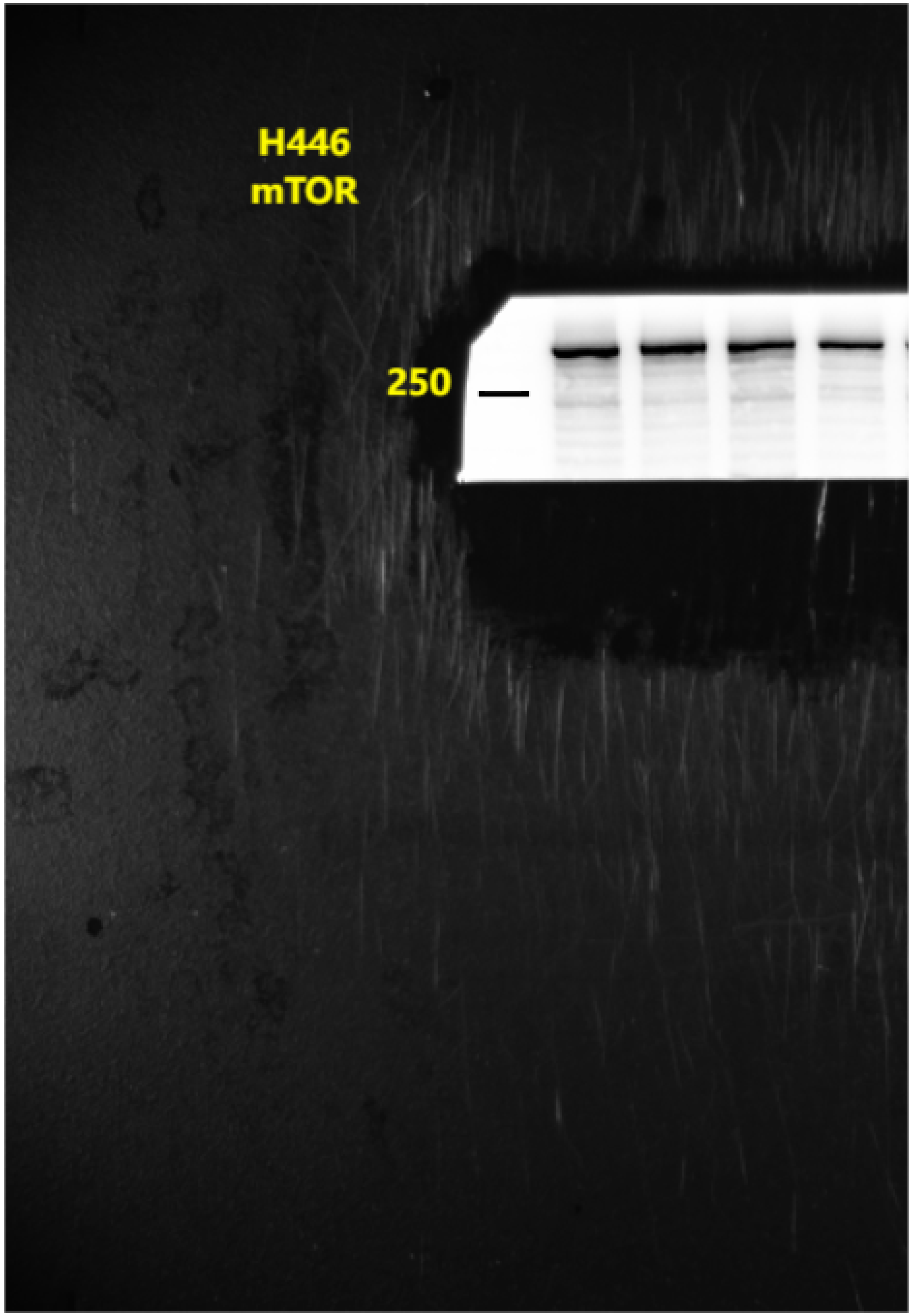
H446
mTOR
250

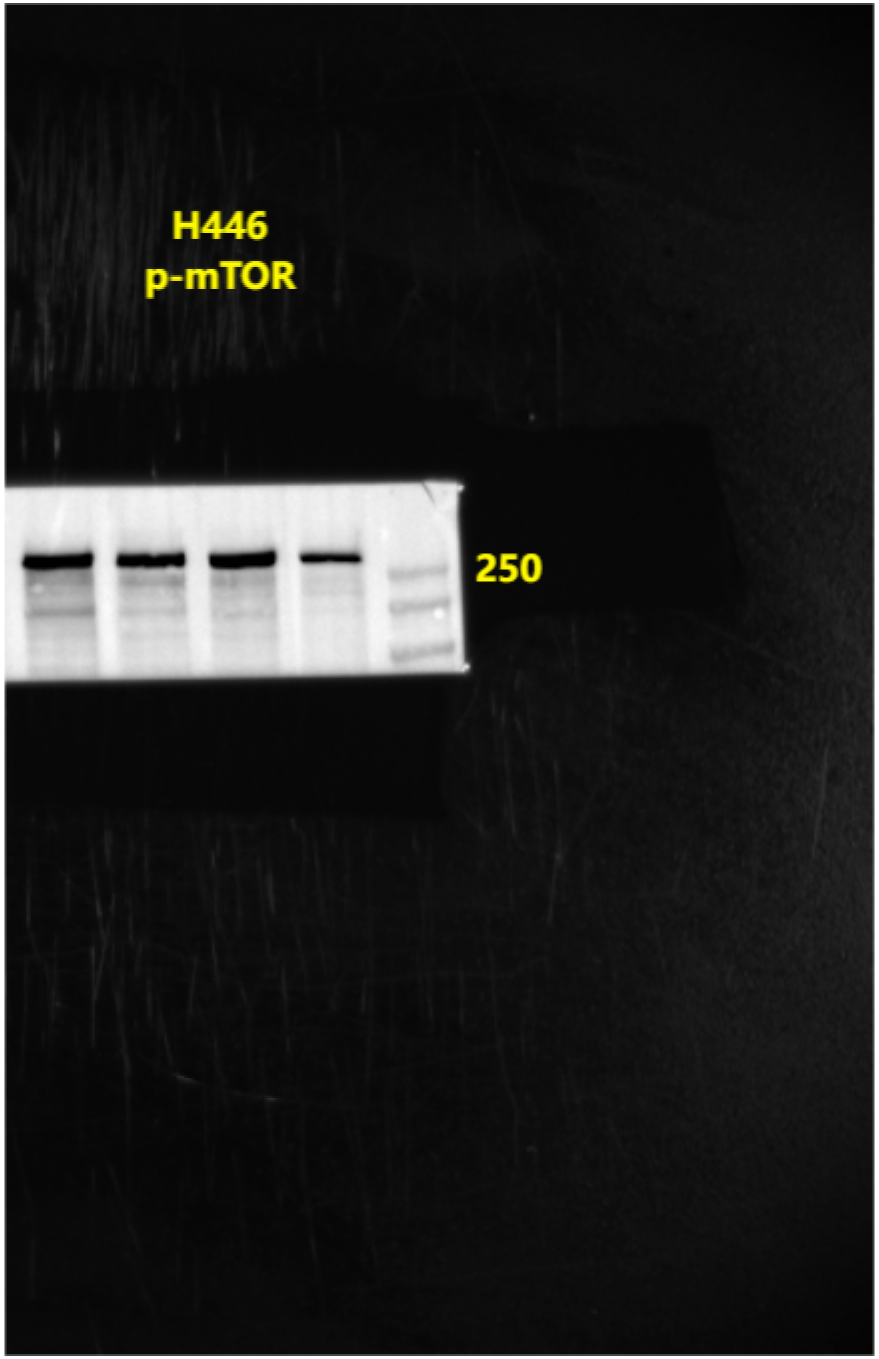
H446
p-mTOR
250

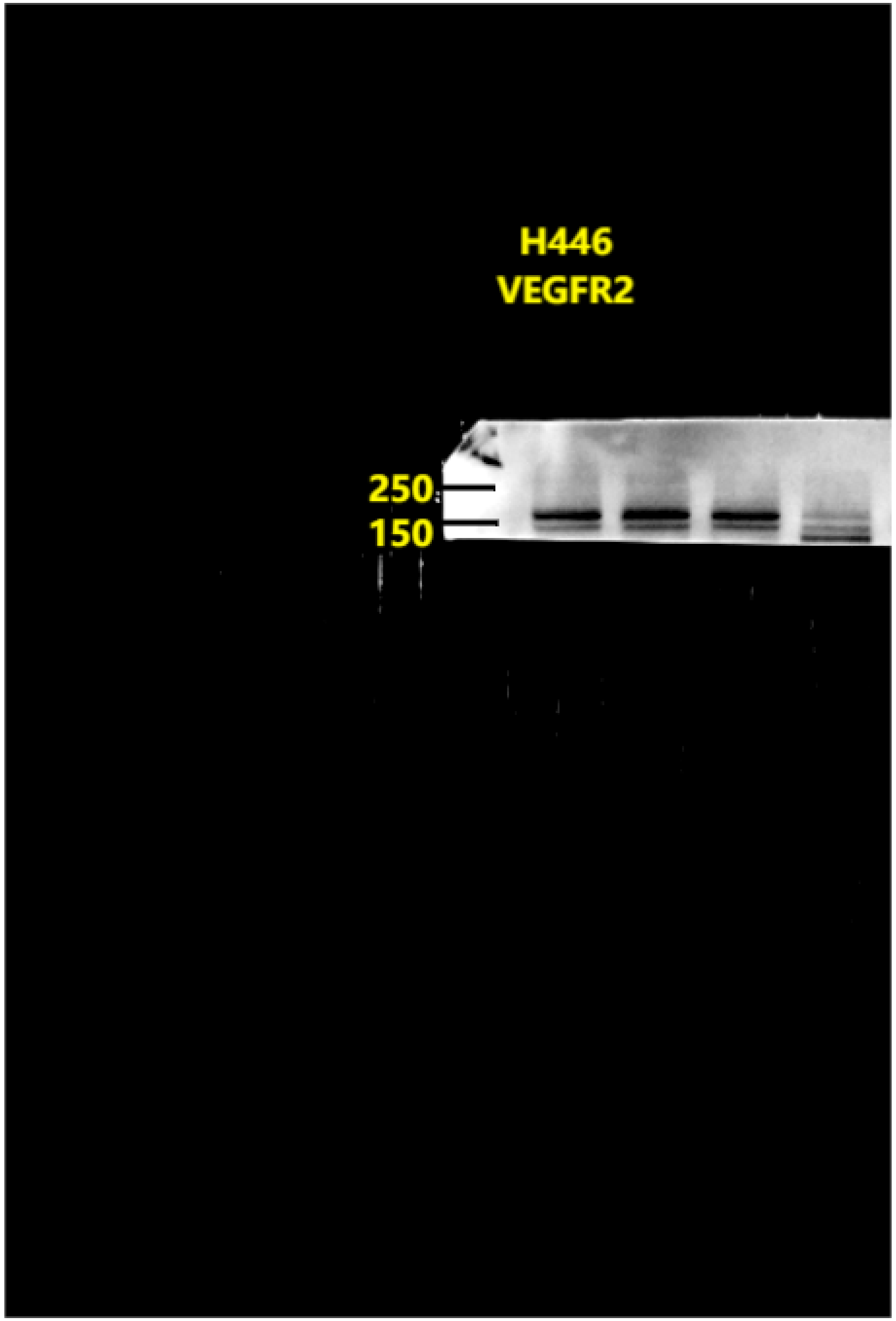
H446
VEGFR2
250
150

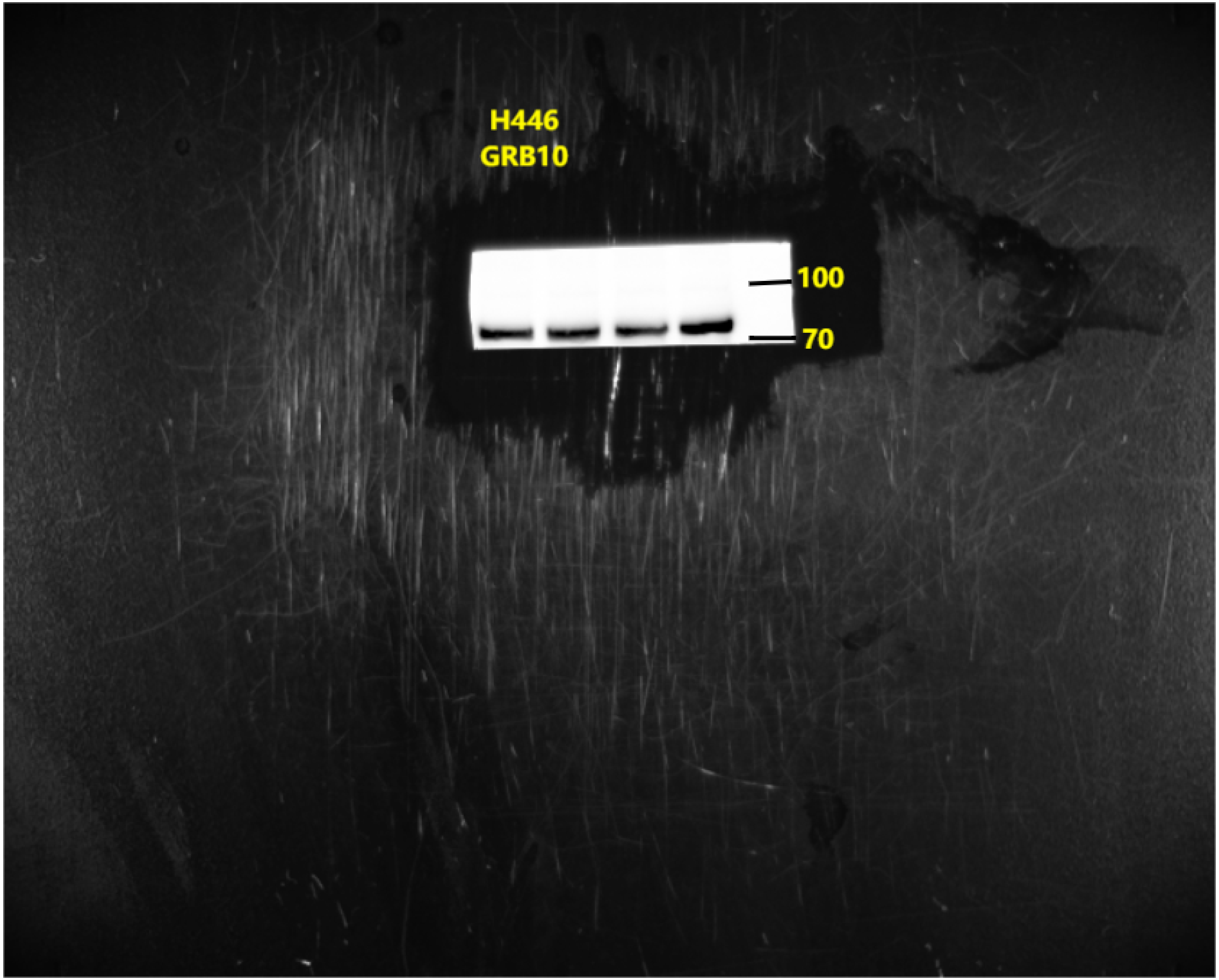
H446
GRB10
100
70

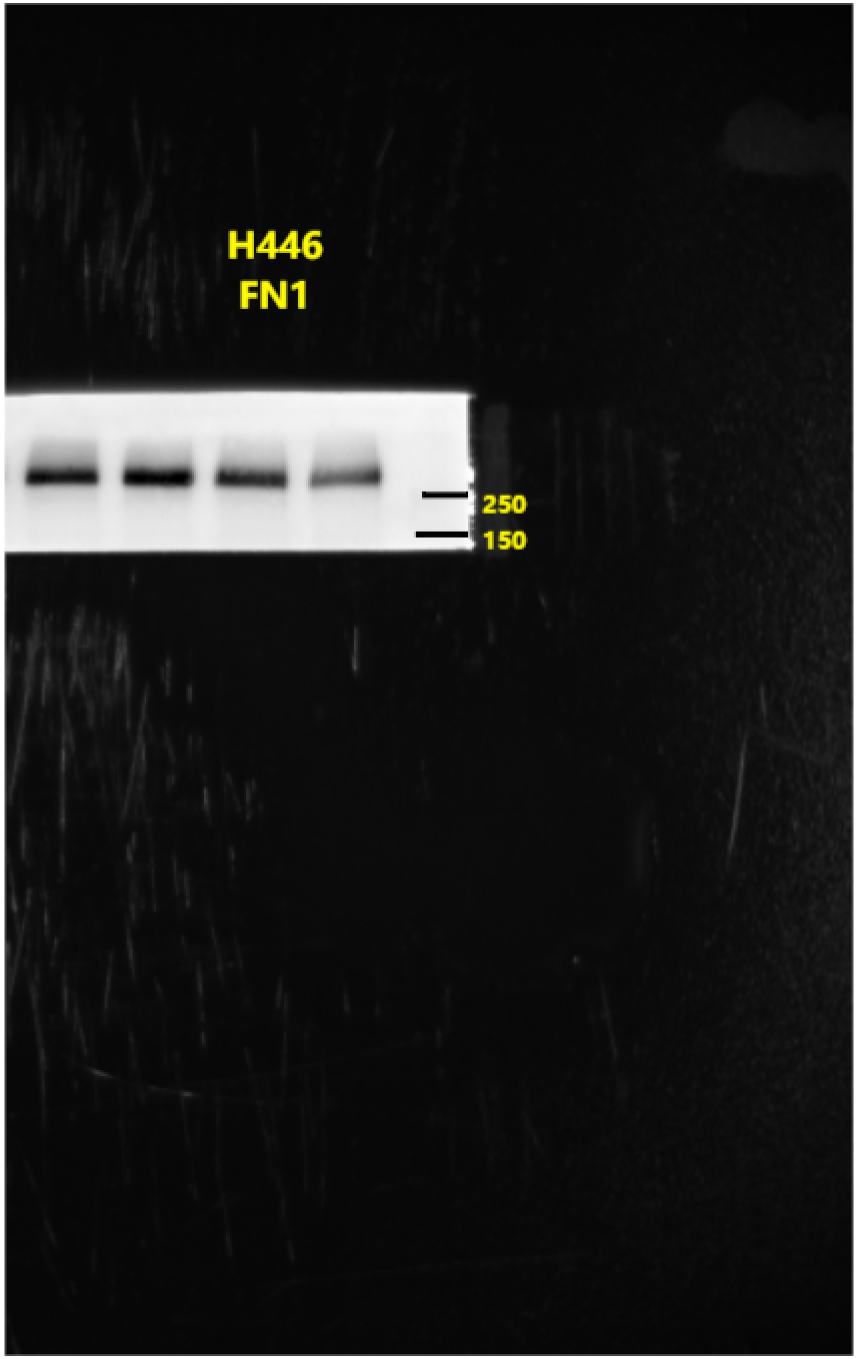
H446
FN1
250
150

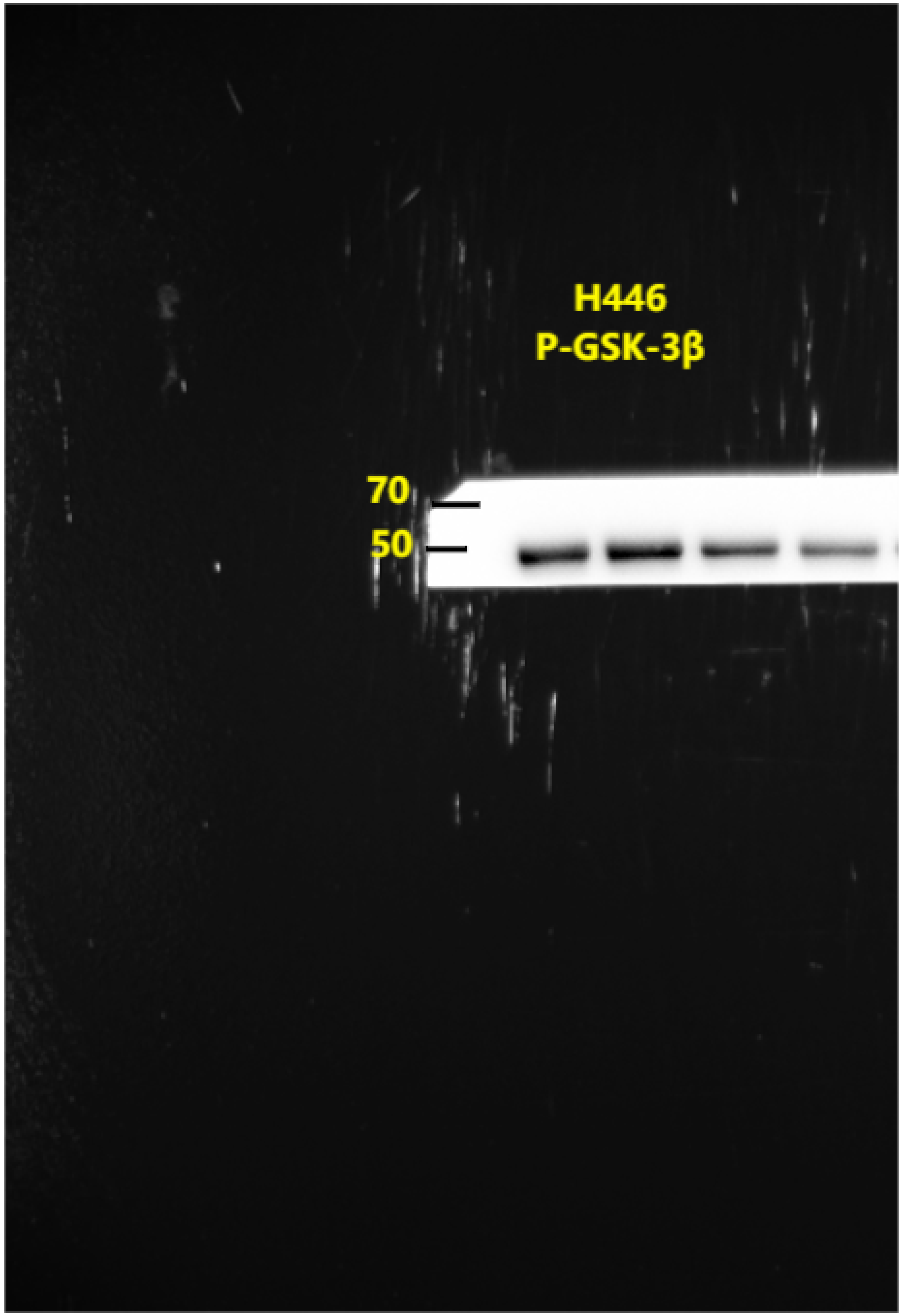
H446
P-GSK-3β
70
50

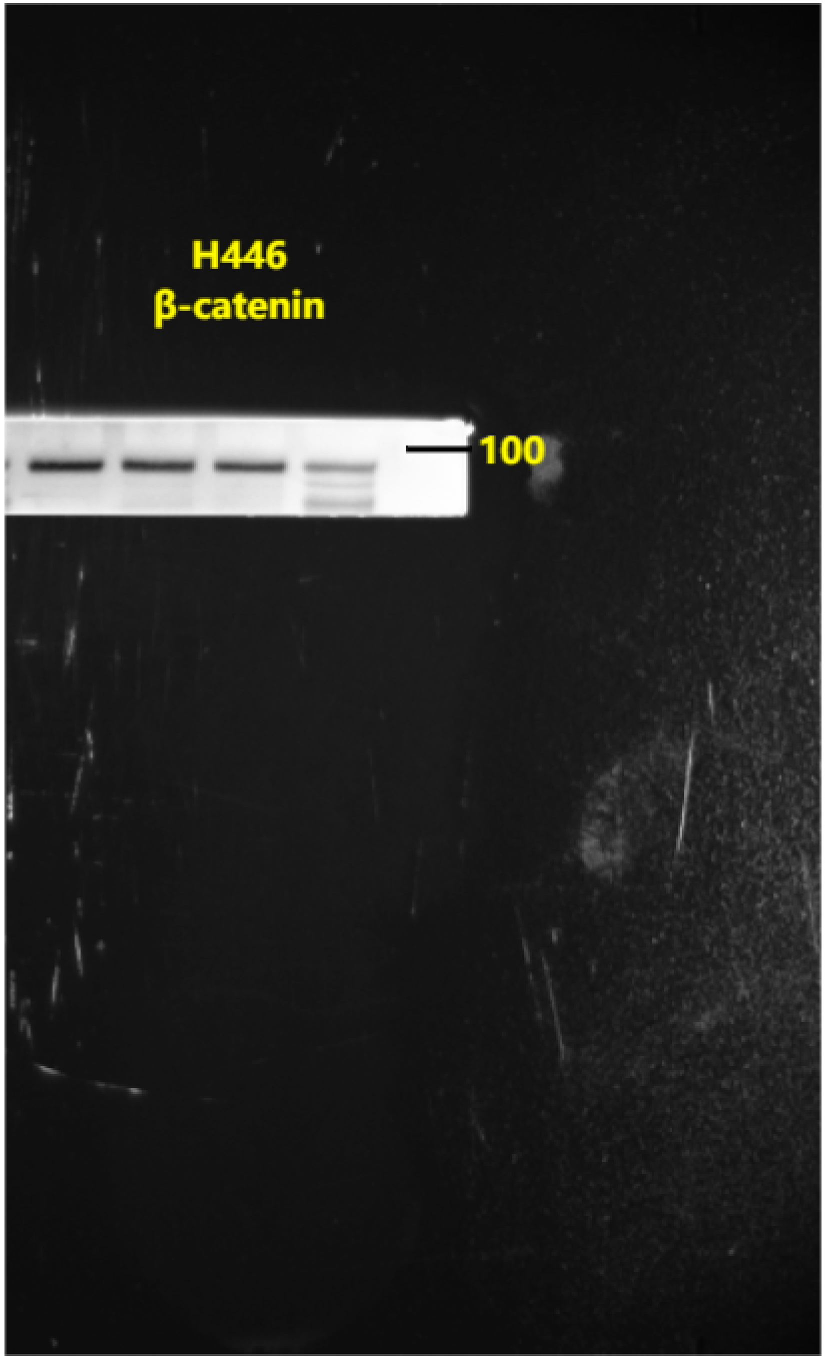
H446
β-catenin
100

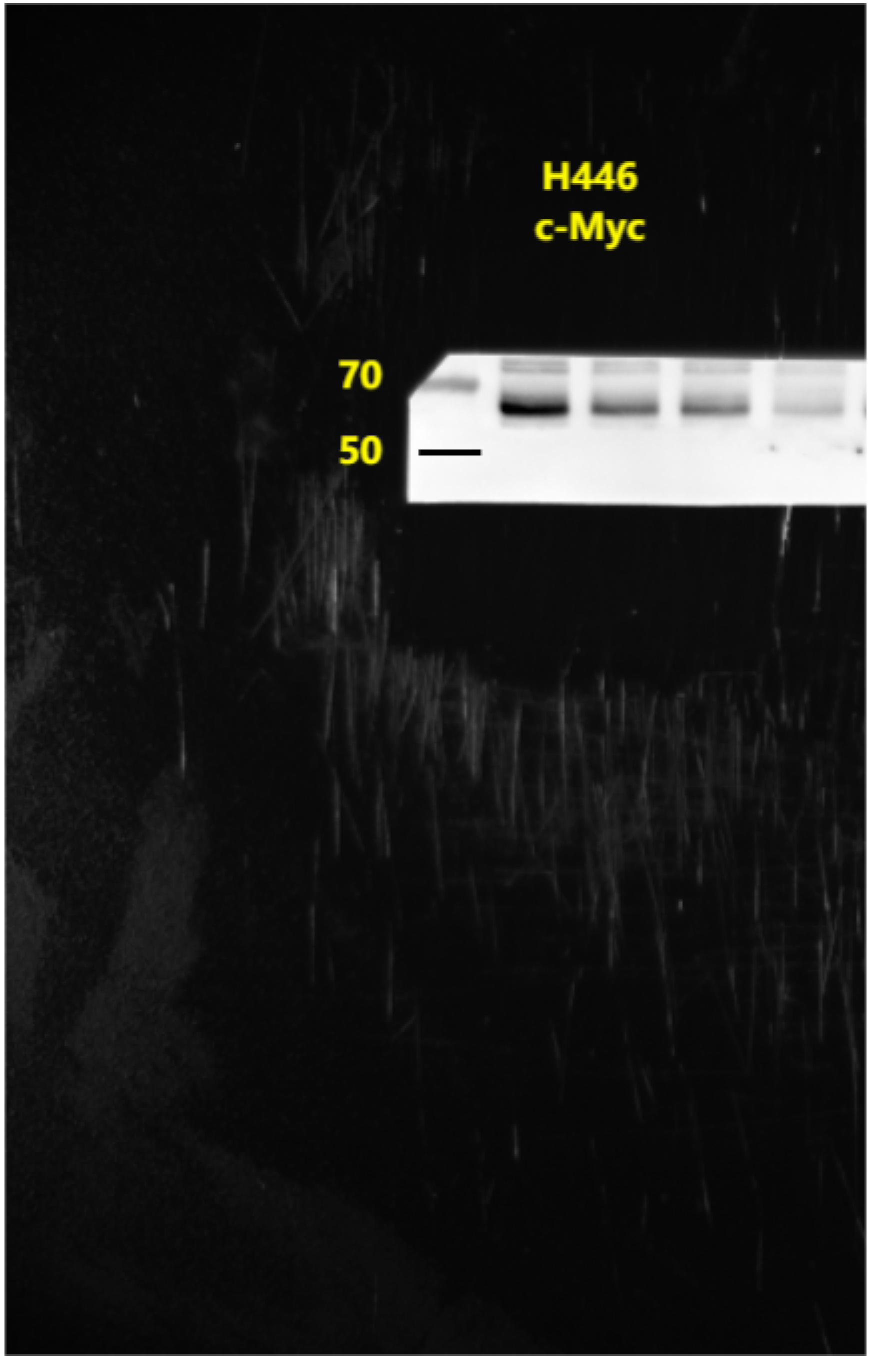
H446
c-Myc
70
50

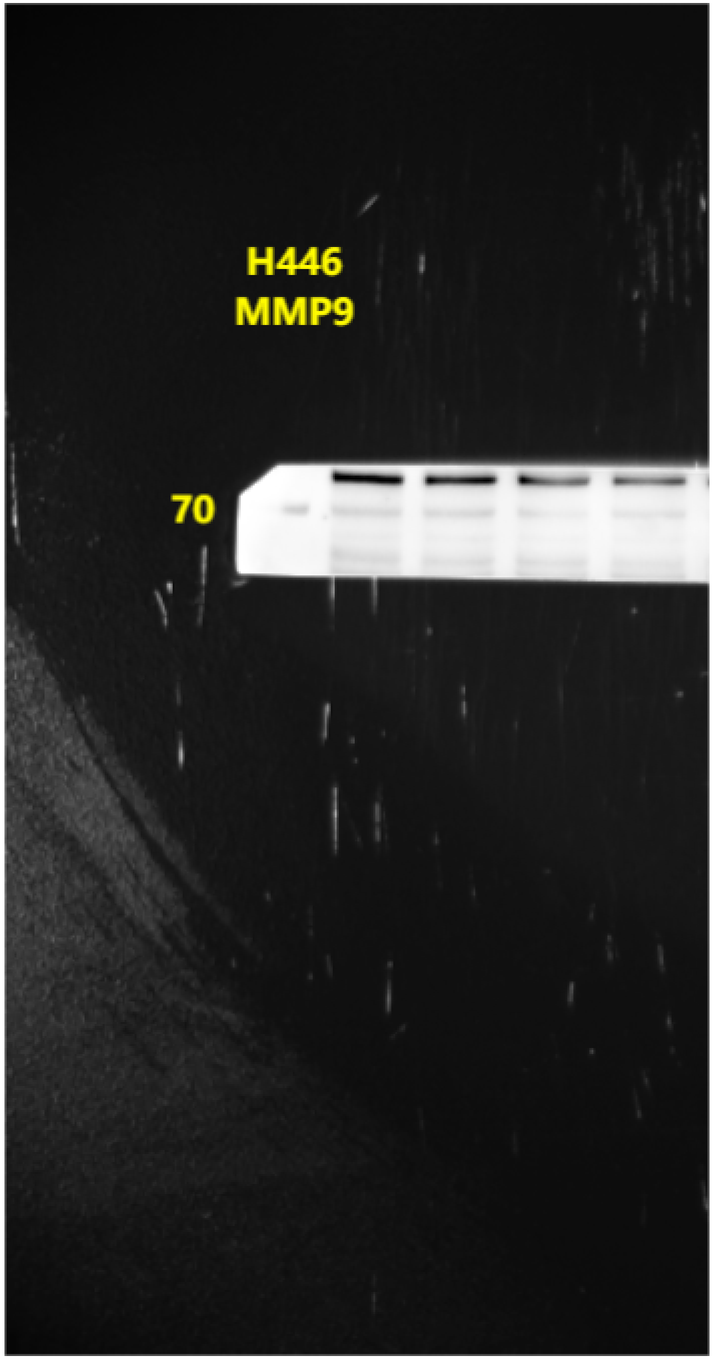
H446
MMP9
70

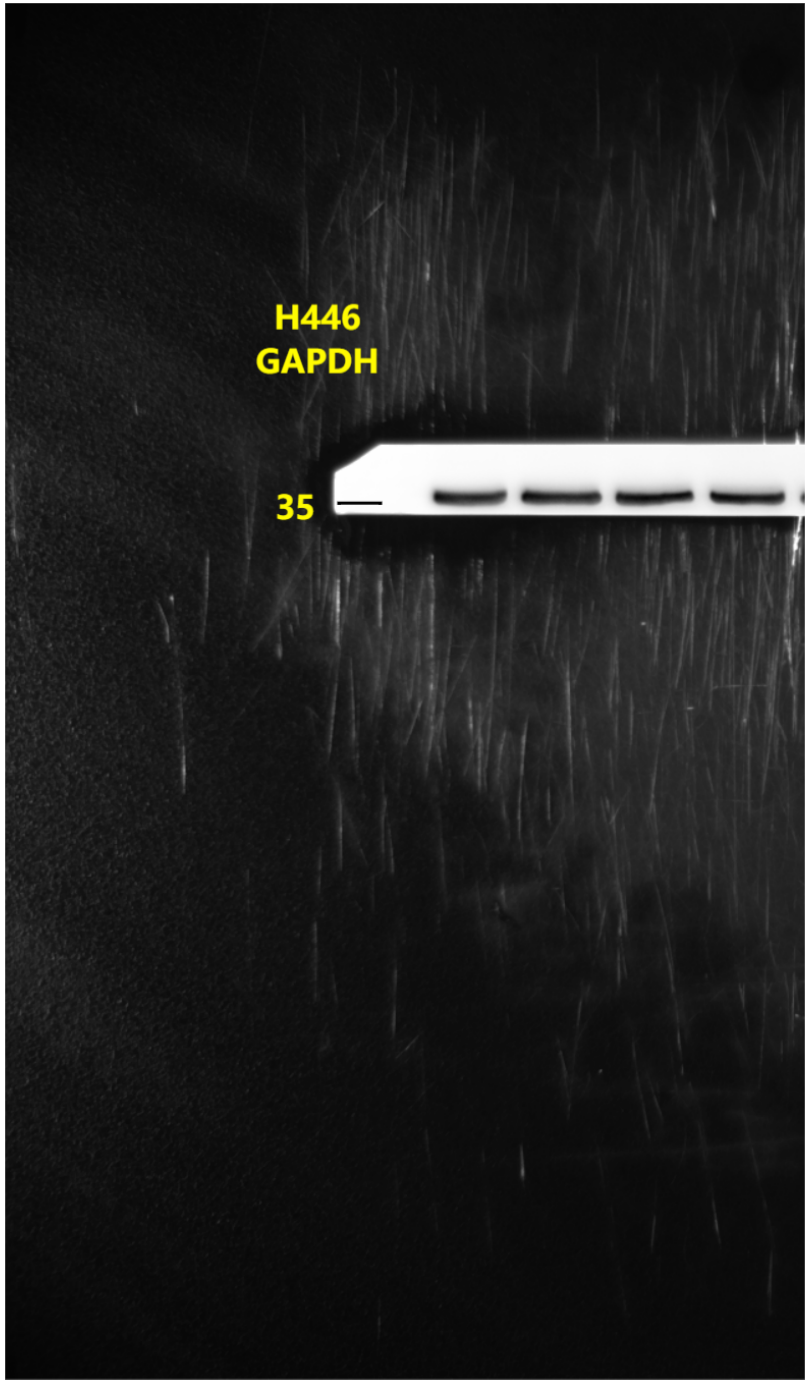
H446
GAPDH
35

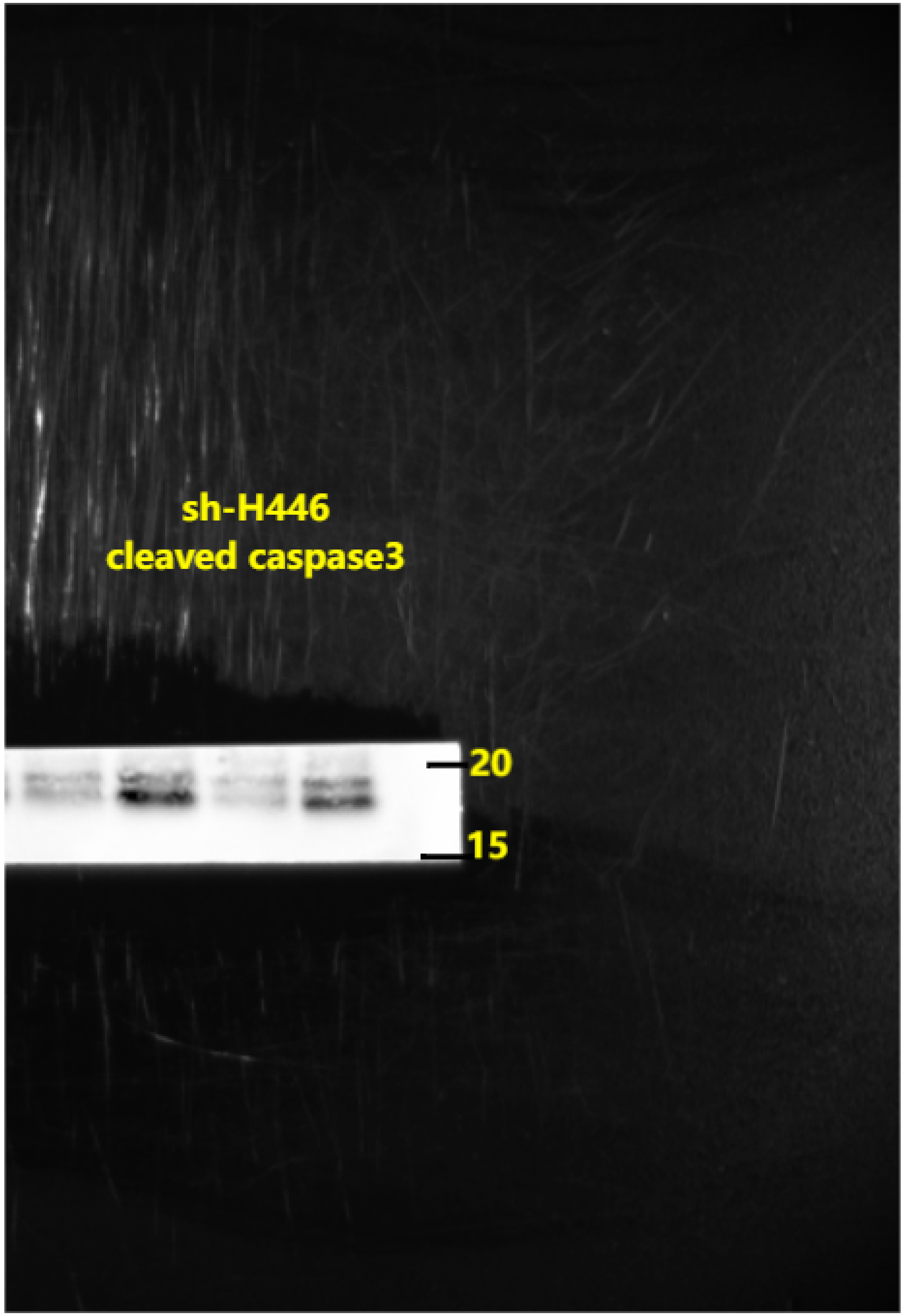
sh-H446
cleaved caspase3
20
15

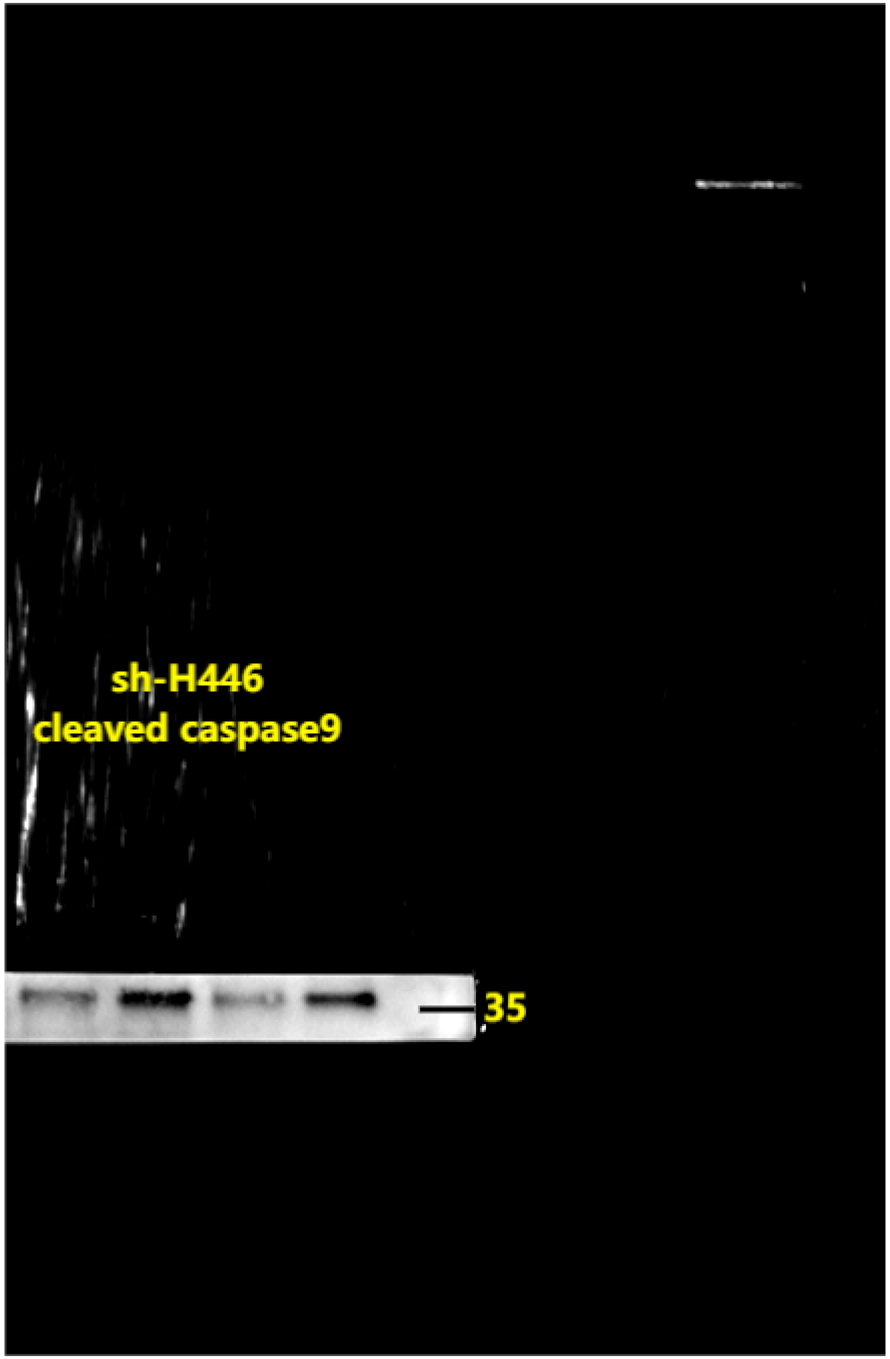
sh-H446
cleaved caspase9
35

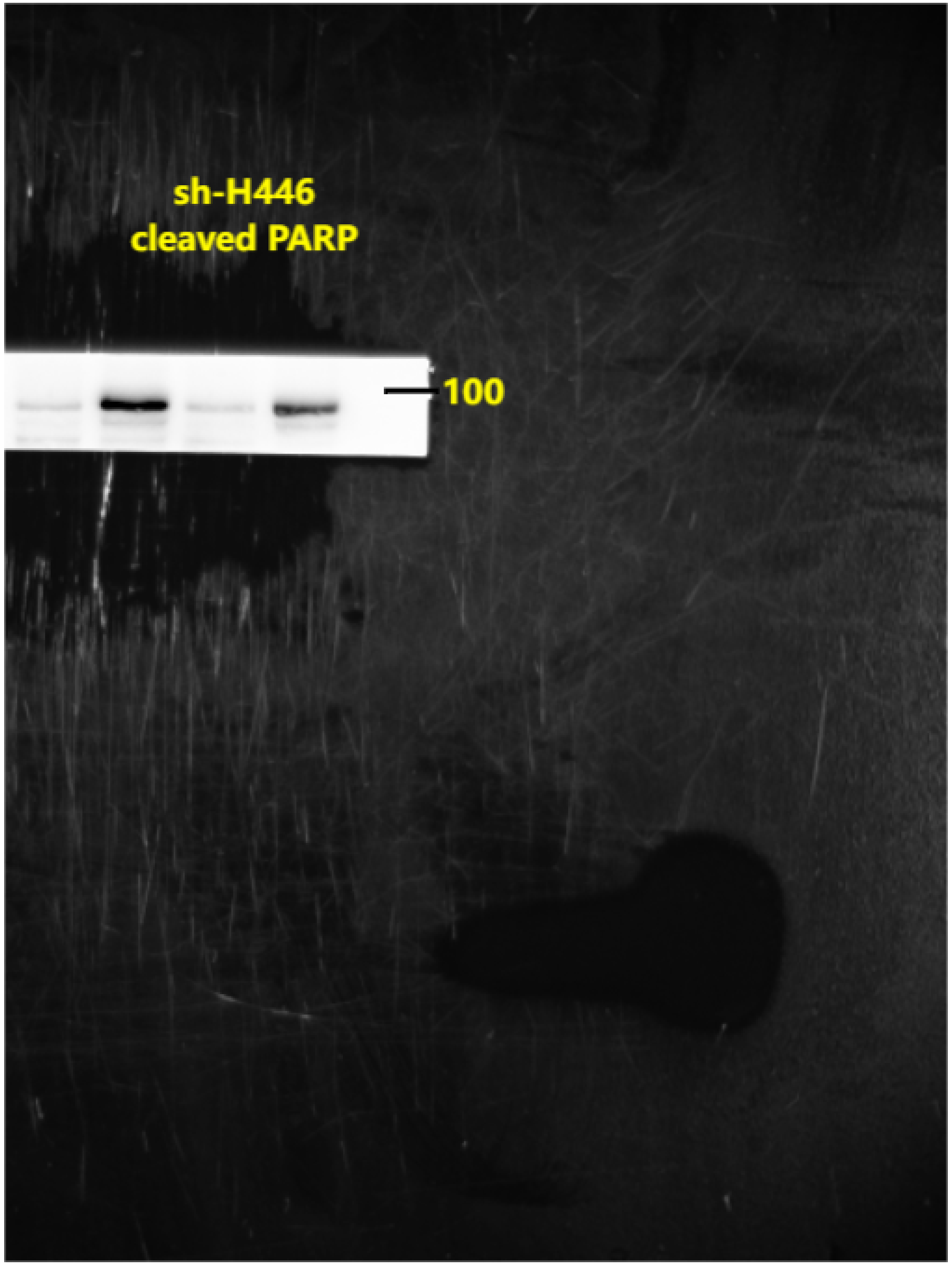
sh-H446
cleaved PARP
100

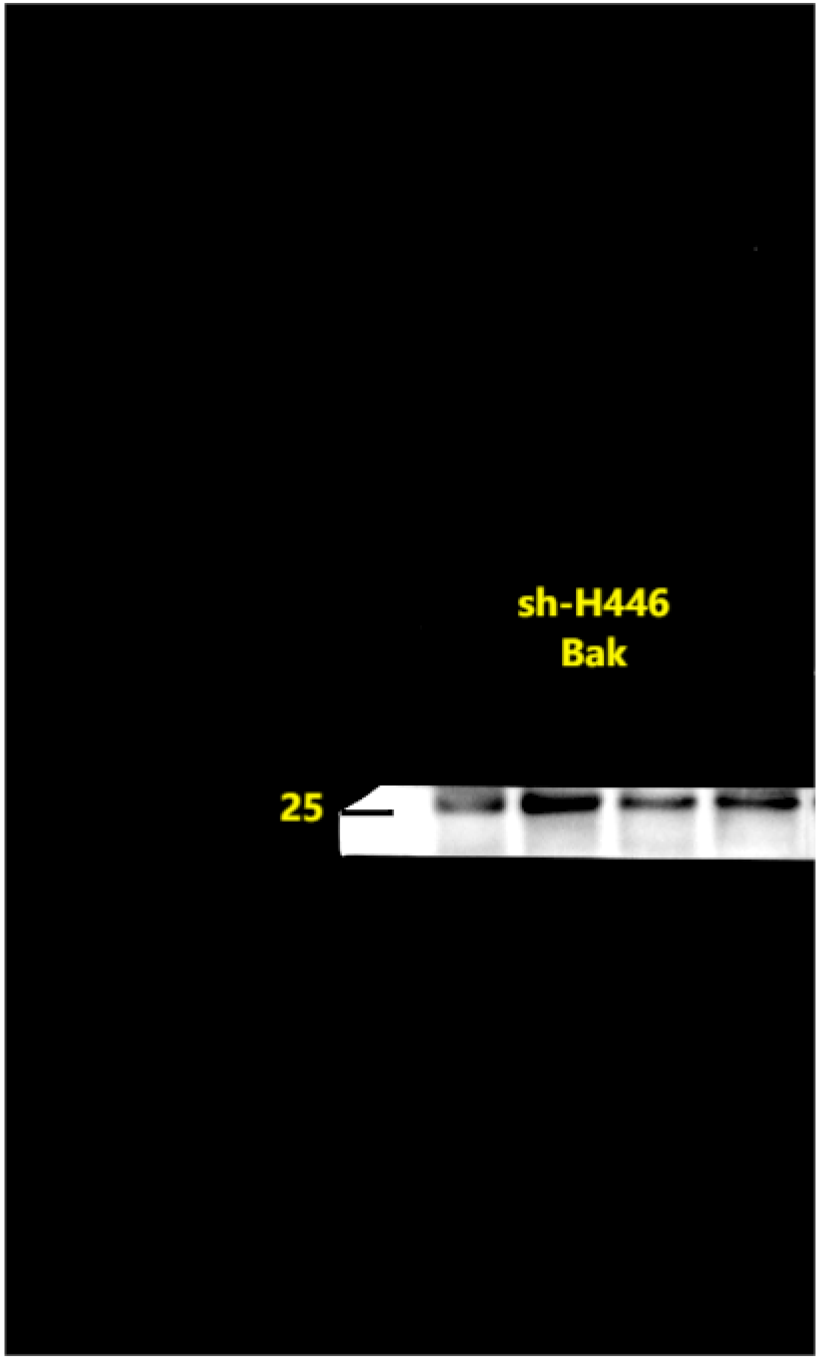
sh-H446
Bak
25

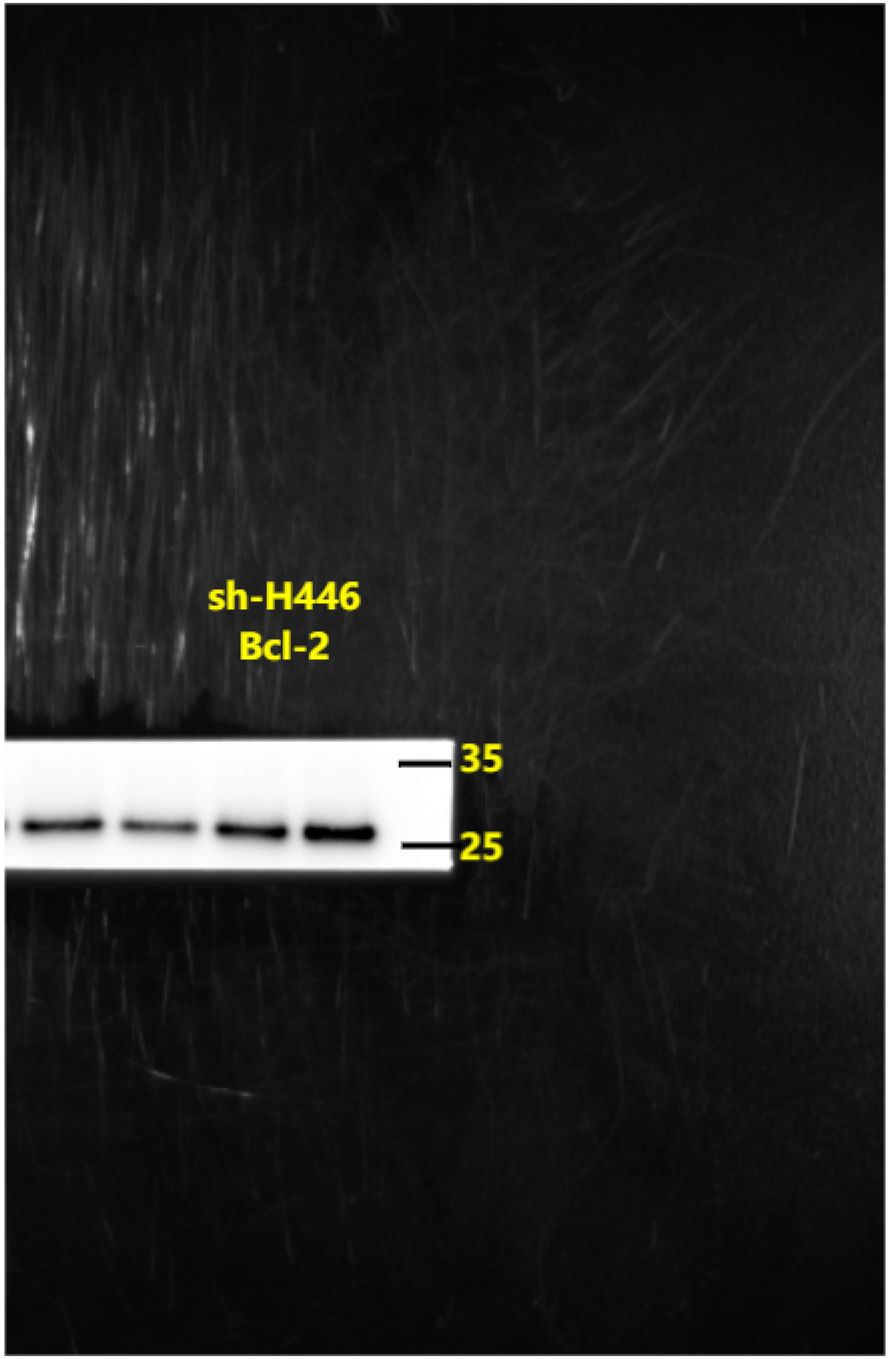

sh-H446
Bcl-2
35
25

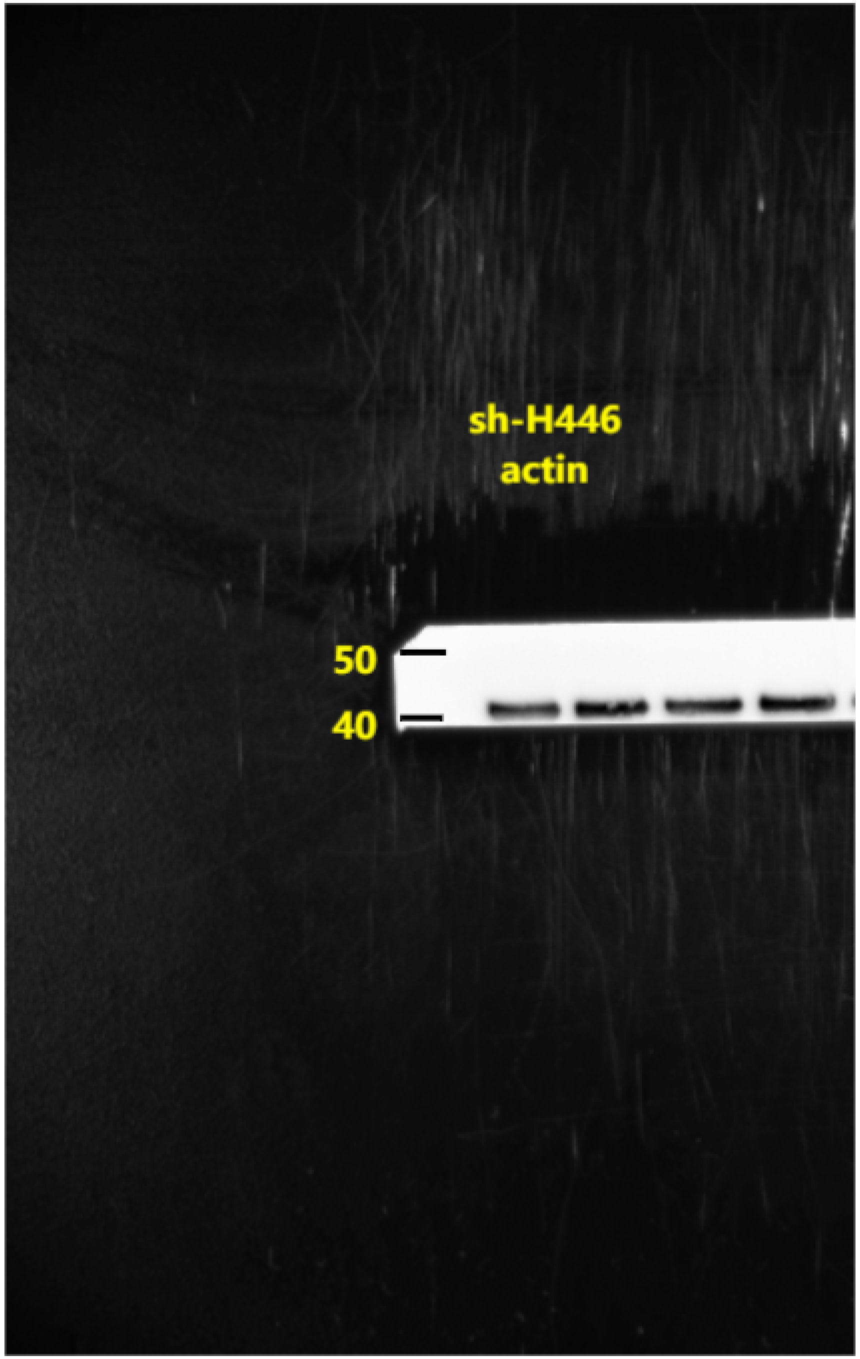
sh-H446
actin
50
40

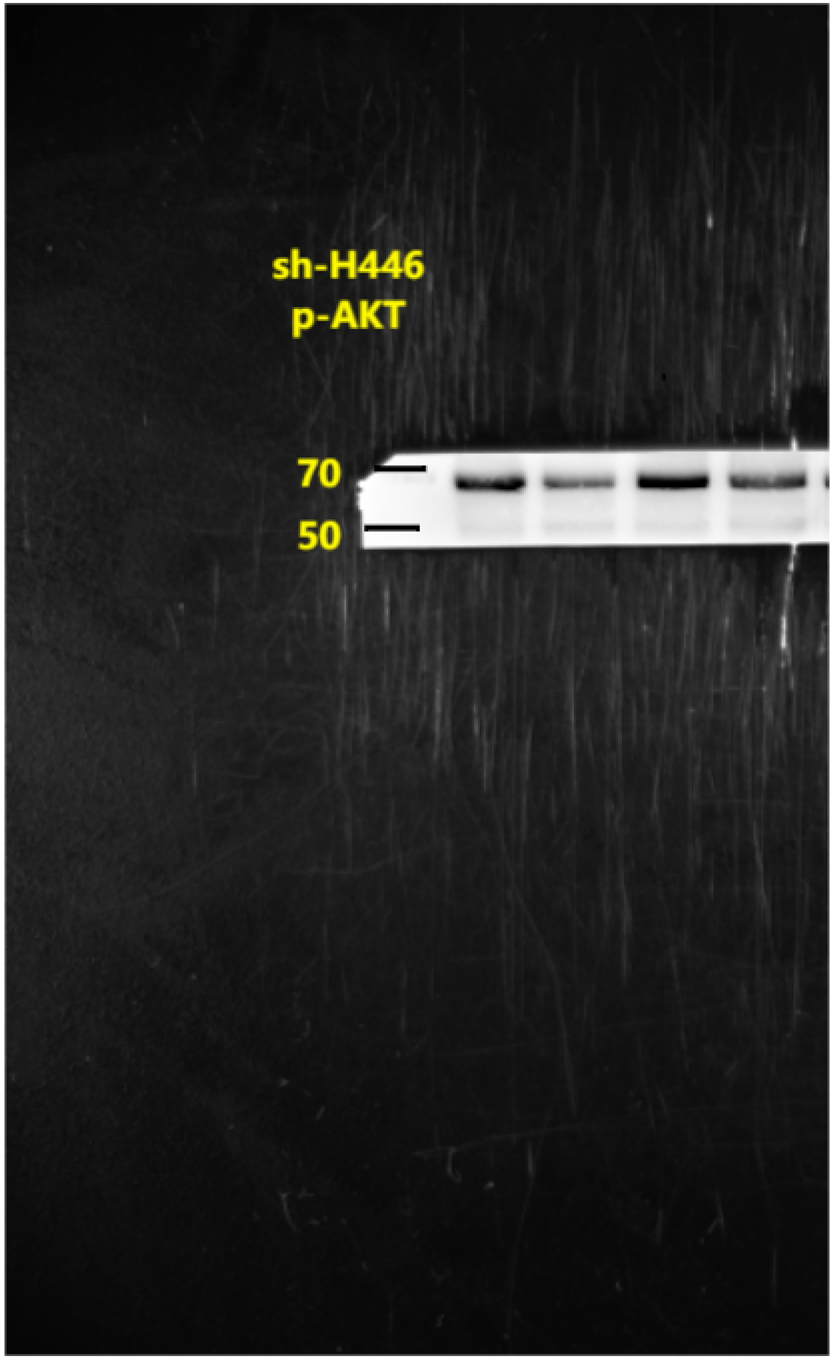
sh-H446
p-AKT
70
50

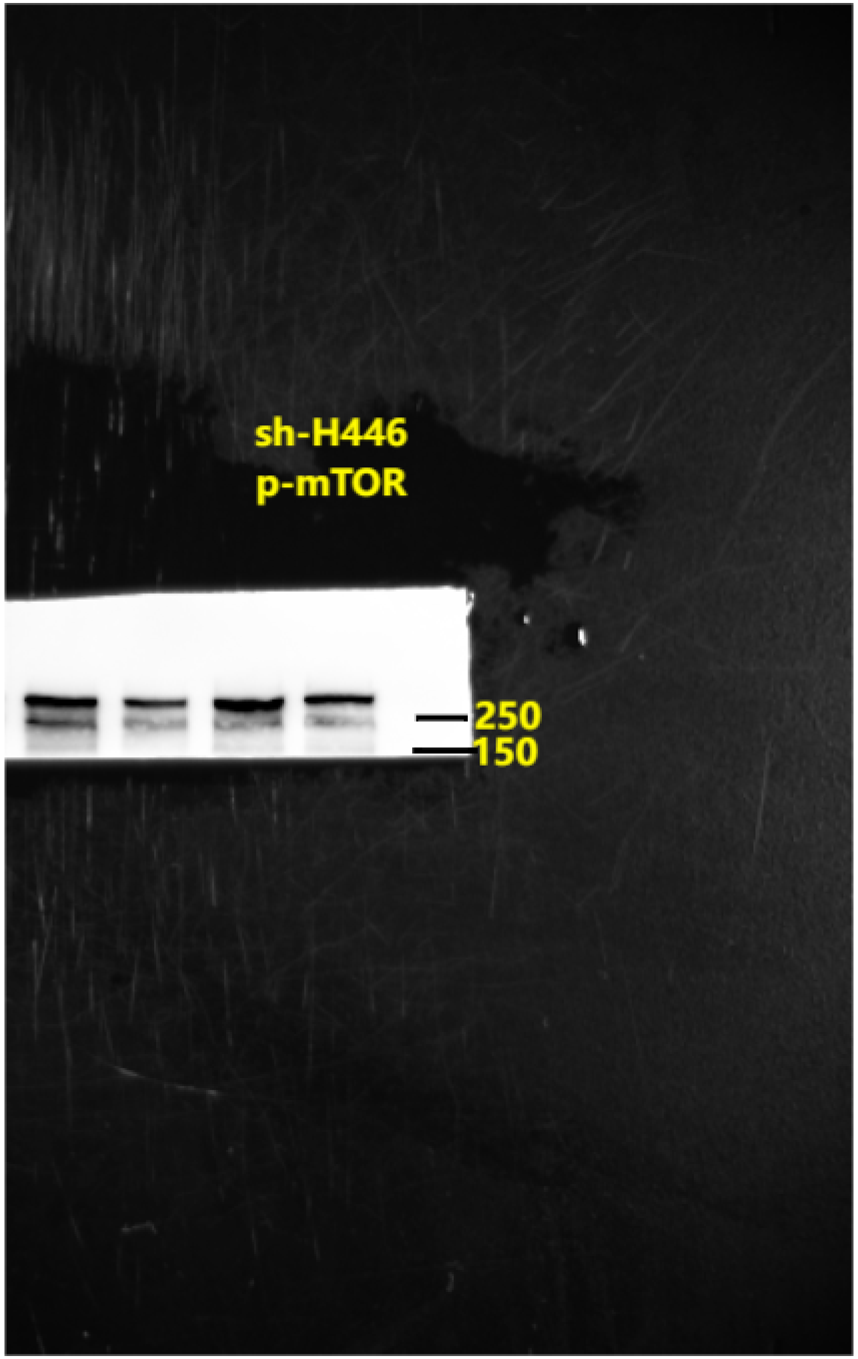
sh-H446
p-mTOR
250
150

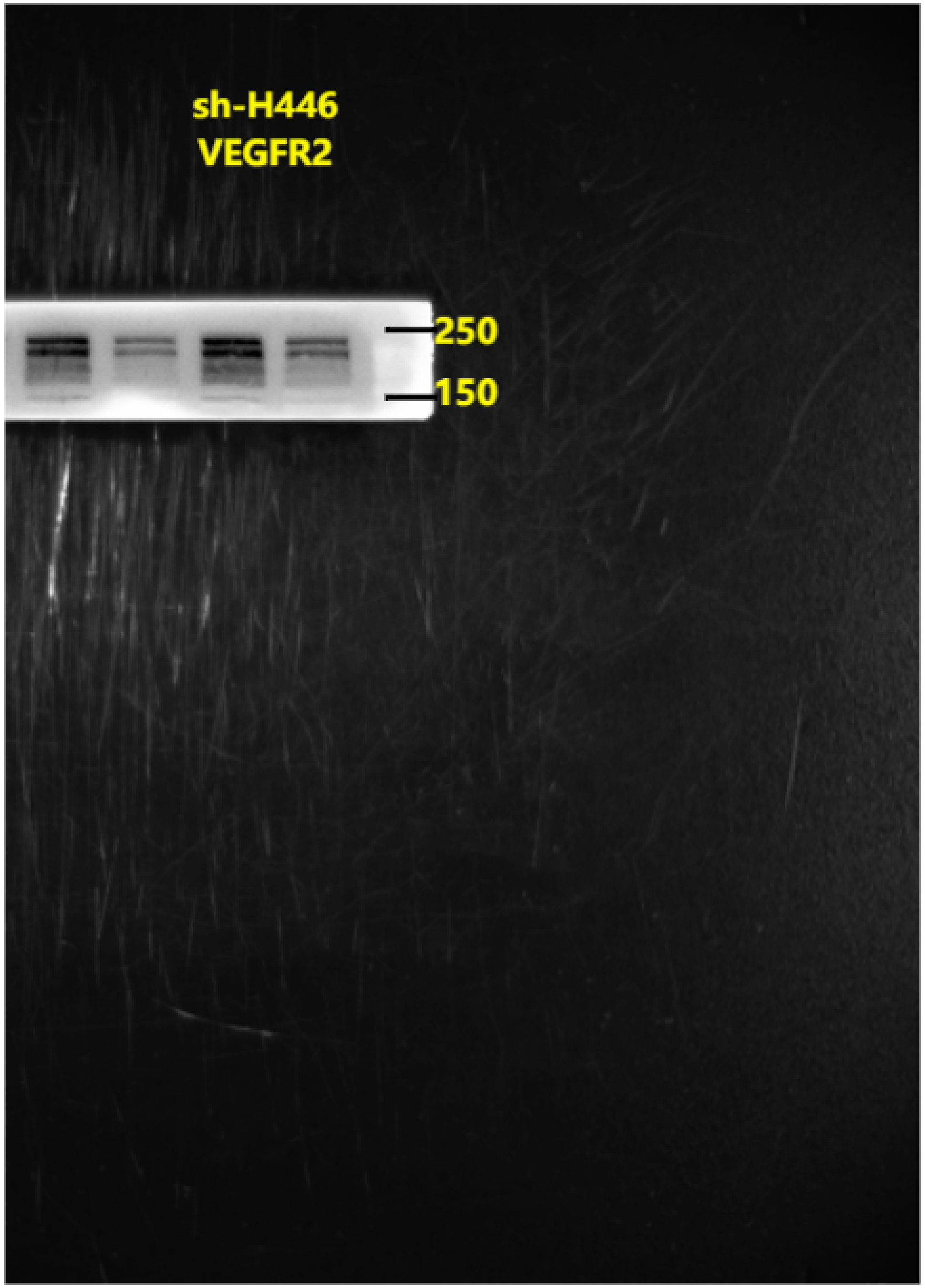
sh-H446
VEGFR2
250
150

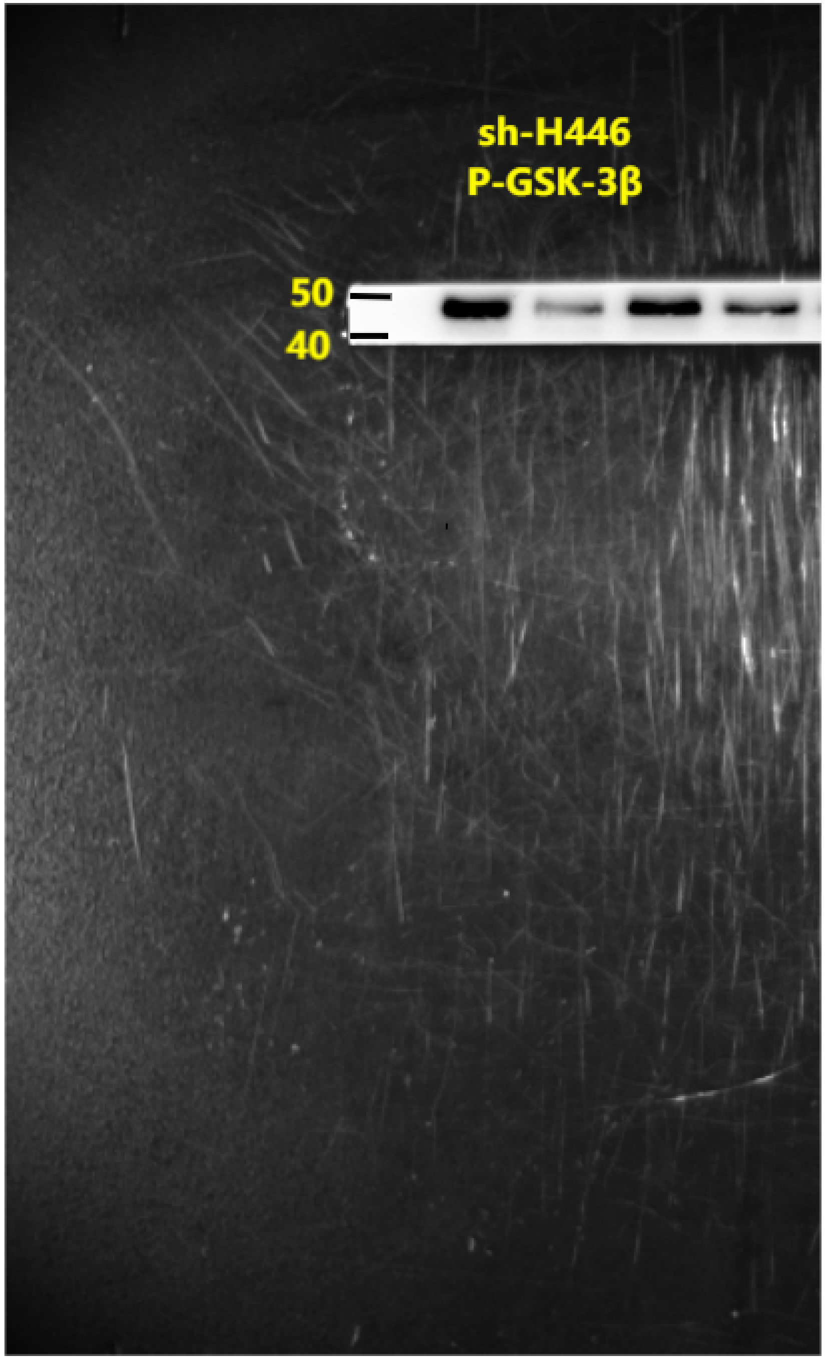
sh-H446
P-GSK-3β
50
40

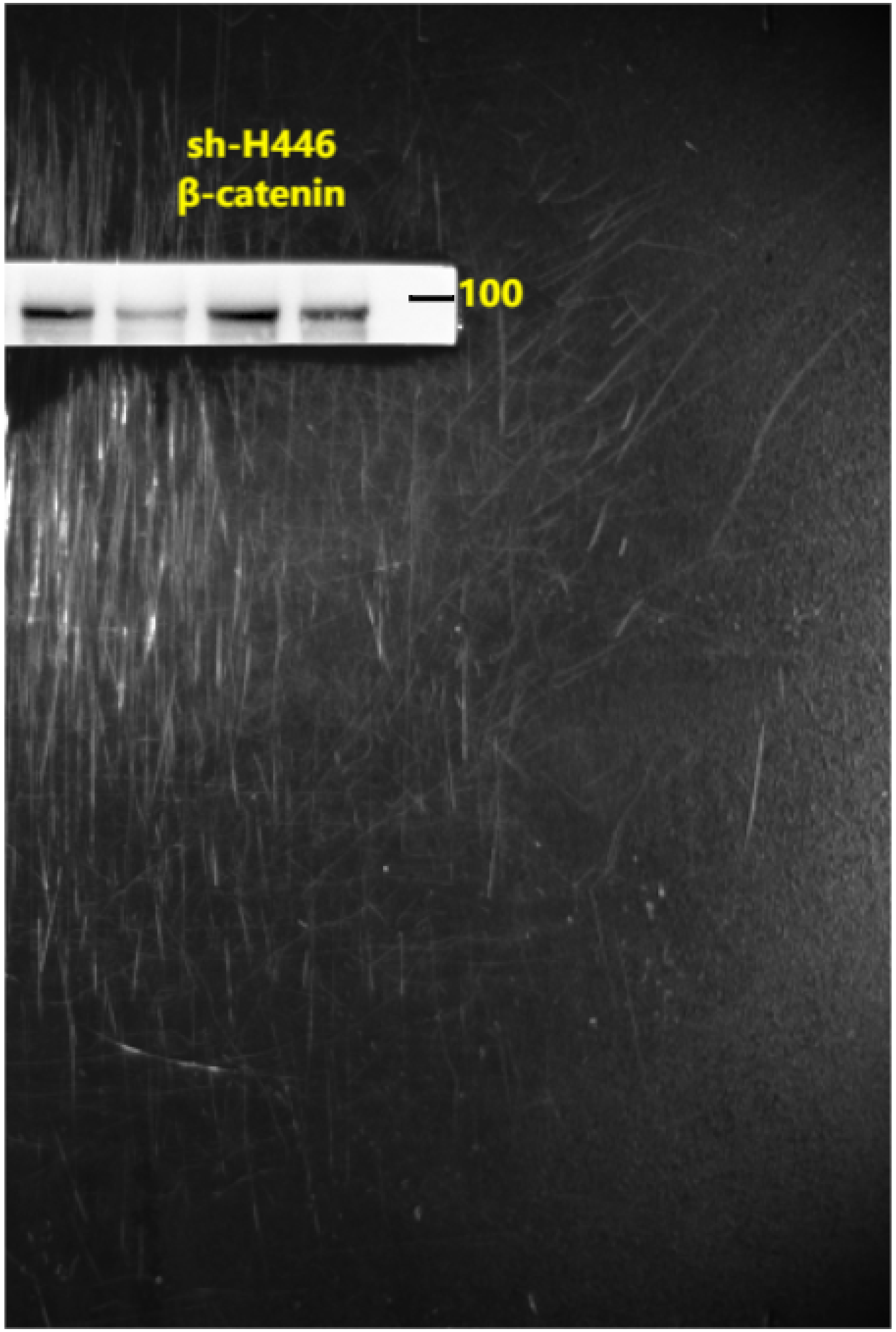
sh-H446
β-catenin
100

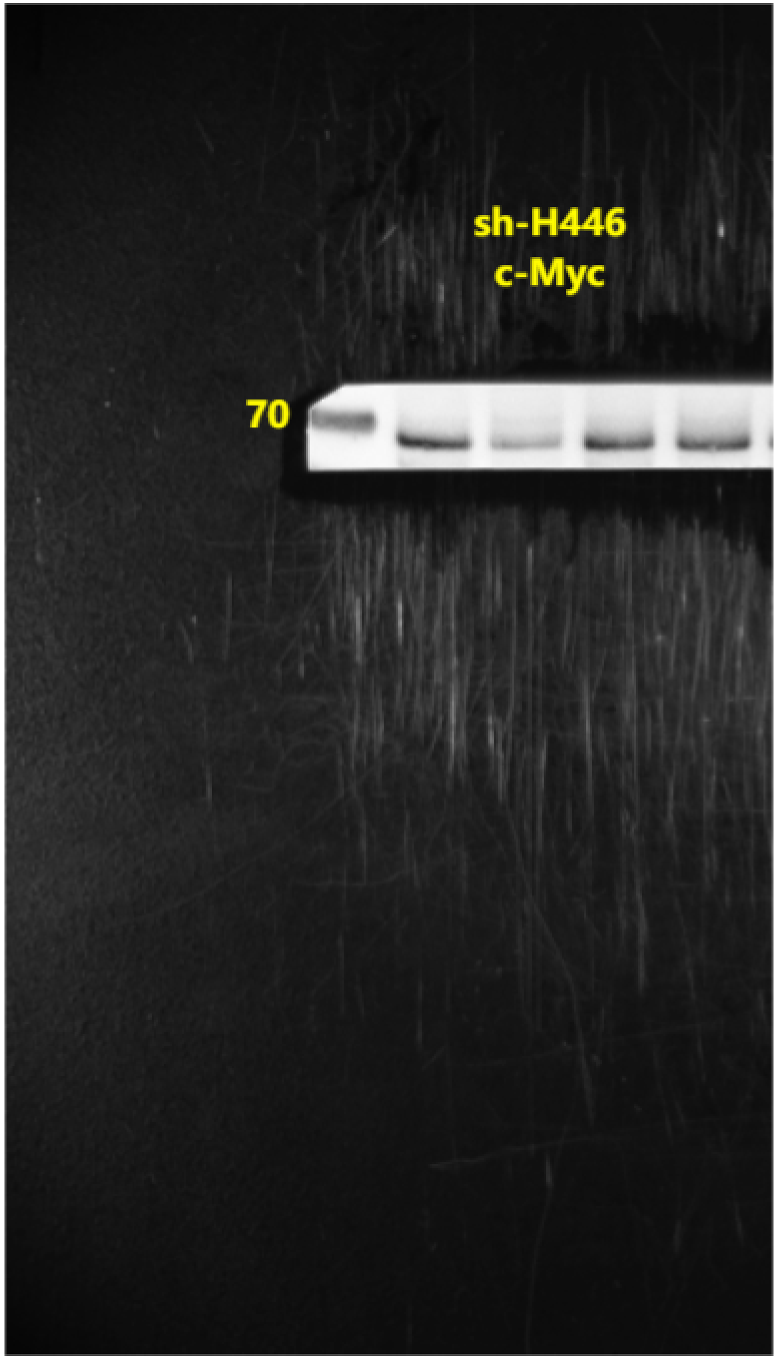
sh-H446
c-Myc
70

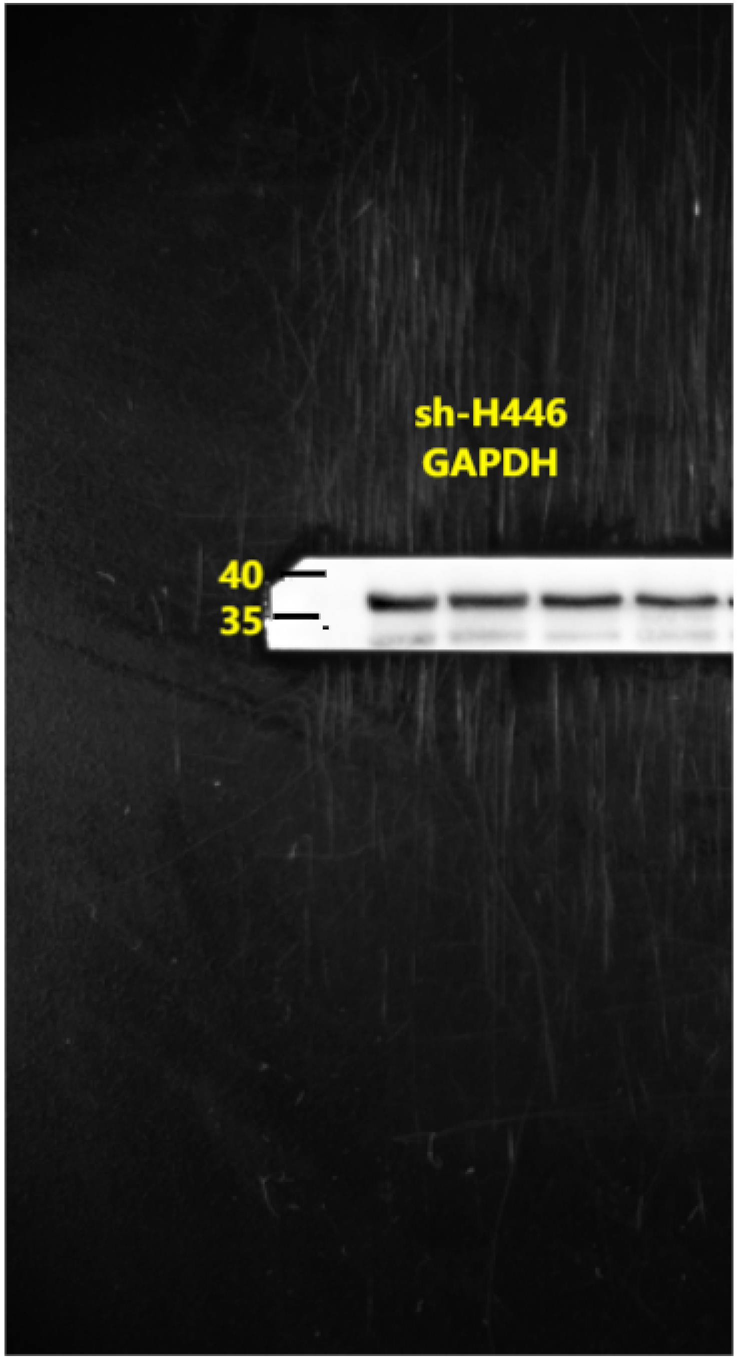
sh-H446
GAPDH
40
35
