# Supplementary material for: Combination of arsenic trioxide and apatinib synergistically inhibits small cell lung cancer by down-regulating VEGFR2/mTOR and Akt/c-Myc signaling pathway via GRB10
Source: Hereditas. 2024 Sep 2;161:29. doi: 10.1186/s41065-024-00330-2 (PMC11367874; doi:10.1186/s41065-024-00330-2)
Supplement: Supplementary file 4 — Supplementary Material 4 [file 41065_2024_330_MOESM4_ESM.docx]

**Supplementary 4**


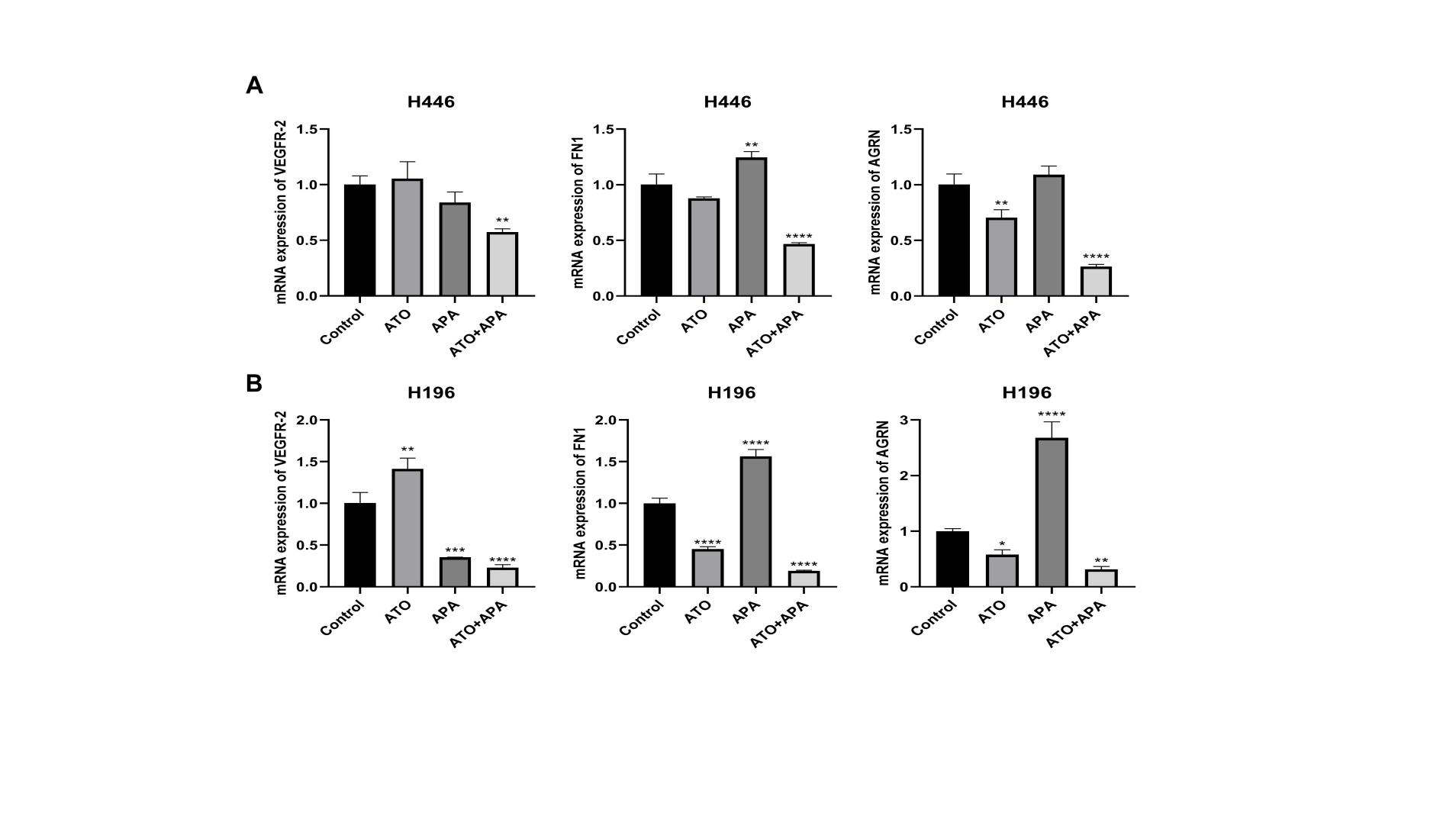


Histograms represent the mRNA level of VEGFR2, FN1, AGRN after the single drug or combination of ATO and apatinib in H446 **(a)** and H196 **(b)** cells for 48 h (****, *P* < 0.0001, ***, *P* < 0.0005, **, *P* < 0.01, *, *P* < 0.05 versus the control group).
